# Low-density lipoprotein balances T cell metabolism and enhances response to anti-PD-1 blockade in a HCT116 spheroid model

**DOI:** 10.3389/fonc.2023.1107484

**Published:** 2023-01-27

**Authors:** Nathalie Babl, Joshua Hofbauer, Carina Matos, Florian Voll, Ayse Nur Menevse, Michael Rechenmacher, Ruth Mair, Philipp Beckhove, Wolfgang Herr, Peter J. Siska, Kathrin Renner, Marina Kreutz, Annette Schnell

**Affiliations:** ^1^ Department of Internal Medicine III, University Hospital Regensburg, Regensburg, Germany; ^2^ Division of Interventional Immunology, Leibniz Institute for Immunotherapy (LIT), Regensburg, Germany

**Keywords:** cholesterol, LDL (low-density lipoprotein), immunotherapy, PD-1, reactive oxygen species, CD154 (CD40L), central memory (T) CM, spheroid model

## Abstract

**Introduction:**

The discovery of immune checkpoints and the development of their specific inhibitors was acclaimed as a major breakthrough in cancer therapy. However, only a limited patient cohort shows sufficient response to therapy. Hence, there is a need for identifying new checkpoints and predictive biomarkers with the objective of overcoming immune escape and resistance to treatment. Having been associated with both, treatment response and failure, LDL seems to be a double-edged sword in anti-PD1 immunotherapy. Being embedded into complex metabolic conditions, the impact of LDL on distinct immune cells has not been sufficiently addressed. Revealing the effects of LDL on T cell performance in tumor immunity may enable individual treatment adjustments in order to enhance the response to routinely administered immunotherapies in different patient populations. The object of this work was to investigate the effect of LDL on T cell activation and tumor immunity *in-vitro*.

**Methods:**

Experiments were performed with different LDL dosages (LDL^low^ = 50 μg/ml and LDL^high^ = 200 μg/ml) referring to medium control. T cell phenotype, cytokines and metabolism were analyzed. The functional relevance of our findings was studied in a HCT116 spheroid model in the context of anti-PD-1 blockade.

**Results:**

The key points of our findings showed that LDL^high^ skewed the CD4^+^ T cell subset into a central memory-like phenotype, enhanced the expression of the co-stimulatory marker CD154 (CD40L) and significantly reduced secretion of IL-10. The exhaustion markers PD-1 and LAG-3 were downregulated on both T cell subsets and phenotypical changes were associated with a balanced T cell metabolism, in particular with a significant decrease of reactive oxygen species (ROS). T cell transfer into a HCT116 spheroid model resulted in a significant reduction of the spheroid viability in presence of an anti-PD-1 antibody combined with LDL^high^.

**Discussion:**

Further research needs to be conducted to fully understand the impact of LDL on T cells in tumor immunity and moreover, to also unravel LDL effects on other lymphocytes and myeloid cells for improving anti-PD-1 immunotherapy. The reason for improved response might be a resilient, less exhausted phenotype with balanced ROS levels.

## 1 Introduction

The discovery of immune checkpoints and development of their specific inhibitors was acclaimed as a major breakthrough in cancer therapy. Especially blocking the inhibitory receptor PD-1 on immune cells and its ligand PD-L1 on immune and tumor cells has been shown to be associated with an enhanced overall survival in metastatic disease of various tumor entities. However, only a limited patient cohort shows sufficient response to therapy (1). Hitherto, numerous biomarkers have been described, predicting response to checkpoint inhibition (2). Recently, cholesterol has been newly identified as a biomarker for the efficacy of PD-1 inhibition (3–5). Consistent with our own results, Perrone et al., Galli et al. and Tong et al. retrospectively showed, that baseline hypercholesterolemia was associated with better outcomes in patients treated with anti-PD-1 checkpoint therapy. In our preliminary exploratory approach (6), we also prospectively demonstrated a positive association.

However, cholesterol seems to be a double-edged sword in tumor immunity, and it`s role in the tumor environment is not fully understood, as other authors have discussed opposing effects. Ma et al. reported a cholesterol induced exhaustion of CD8^+^ T Cells in the tumor microenvironment and furthermore, Khojandi et al. observed a promoted resistance to cancer immunotherapy by oxidized lipoproteins, amongst others mediated by suppression of T cell immunity (7, 8). The reason for these seemingly paradox findings may be due to the embedment of cholesterol in different complex metabolic conditions. Cholesterol has been identified as a biomarker in cachexia and the metabolic syndrome (9, 10). Furthermore, hypercholesterolemia has been associated with atherosclerosis, Alzheimer`s disease, cancer and may exacerbate autoimmune diseases by inducing hyper-activated T cells (11–15).

Up to date, mainly macrophages have been perceived as a link between cholesterol and different diseases, however there is growing evidence for T cells also playing a crucial role (16). Although the details of cholesterol homeostasis have been investigated mainly in hepatocytes and macrophages, the mechanisms of cholesterol biosynthesis, uptake, esterification, and efflux also apply to T cells (11, 12). Furthermore, it has been acknowledged that T cells express the LDL receptor, however it is not clear, if cholesterol uptake is conducted exclusively *via* the LDL receptor (17). Cholesterol maintains quiescence in naïve T cells and also paradoxically regulates exit from quiescence by modulating TCR nanocluster formation besides effecting signaling molecules (18–20). T cell activation induces an increase of intracellular cholesterol for proliferation, however self-regulation is secured by negative feedback pathways (21–24). Moreover, cholesterol is also involved in the differentiation and stabilization of the different T cell subsets. While Th1, Th17, γδT, and cytotoxic T cells require high cholesterol levels, Th2 cells do not (25–28). Paradoxical effects have also been observed in Tregs and memory T cells (29, 30). There are indications that CD8 memory T cells might require suppression of the cholesterol pathway, while contrarily CD4 memory T cells depend upon enhanced cholesterol levels (11, 31, 32).

So, being confronted with very paradox findings in complex environments, we aimed to straightforwardly investigate the effects of cholesterol on T cell subsets. We focused on LDL, since LDL emerged as the most significant serum lipid associated with response to immunotherapy in our and another preceding study (6, 33).

The LDL dosages for treatment referring to medium control were chosen according to LDL serum levels and their estimated tissue levels in responders (LDL^high^) and non-responders (LDL^low^) to anti-PD-1 checkpoint therapy (6, 34).

We analyzed the T cell phenotype, considering checkpoint markers, activation markers, co-stimulatory markers und effector versus memory markers. Furthermore, we investigated T cell metabolism including mitochondrial metabolism, cholesterol uptake, ROS accumulation, cell respiration and acidification. In order to further explore the functional relevance of our findings in the context of tumor immunity and PD-1 blockade, we established a co-culture model with T cells migrating into colorectal cancer HCT116 tumor spheroids.

## 2 Material and methods

### 2.1 Cell culture

Buffy coats from healthy donors were obtained from the Department of Transfusion Medicine (University Hospital Regensburg) in form of remnants from routine platelet donations. The donations were approved by the Institutional Ethics Committee of the University of Regensburg (vote number 13-101-0240; 13-101-0238) and are in accordance with the Declaration of Helsinki.

CD4^+^ and CD8^+^ T-cells were isolated using MACS cell separation kits (Miltenyi Biotec, 130-096-533 (CD4), 130-096-495 (CD8)). After isolation, cells were stored over night at 37°C at a concentration of 10^7^ cells per ml in RPMI 1640 medium supplemented with 2% human AB Serum, 1% stabilized glutamine and 0,5% Penicillin/Streptomycin. Cells were washed, counted (CASY System) and the cell concentration was adjusted to 5x10^5^ cells per ml using the aforementioned medium plus 100 IU recombinant human (rh) IL-2 (PeproTech, 200-02) per ml. Either PBS (control) or LDL (Kalen Biomedical LLC, 770200-1) was added at the indicated concentrations and cells were seeded in 96 well U-Bottom plates. For some of the experiments as indicated, an anti-human PD-1 blocking antibody (InVivoMAb anti-human PD-1, Bio X Cell, BE0188) was added at a concentration of 10 µg/ml. T cells were stimulated using CD3/28 Dynabeads (ThermoFisher Scientific, cell to bead ratio 1:1, 25 µl bead suspension per 10^6^ cells), incubating at 37°C for 96h. The T cells were harvested after 48h and 96h, Dynabeads were removed and the cells were counted using the CASY System.

### 2.2 Co-culture of T cells withtumor spheroids

Spheroids were generated for 4 days on ultra-low attaching plates, which were coated with a solution of 12 mg/ml Poly-(methacrylsäure-2-hydroxyethylester) (poly-HEMA; Sigma Aldrich) dissolved in 95% ethanol. 50 µl of sterile poly-HEMA solution per well was allowed to evaporate in a sterile biosafety cabinet and dried plates were stored at 4°C before use. Sub-confluent cultures of the highly microsatellite instable colon carcinoma cell line HCT116 (35) were dissociated into single-cell suspensions and subsequently, 10,000 cells in 100 µl RPMI 1640 (GIBCO, 31870-025) with 10% fetal calf serum (Sigma, F7524) and 2 mM glutamine (PAN Biotech, P04-80100)) were seeded per well and incubated in a humidified atmosphere (5% CO2, 95% air) at 37°C (Heraeus Incubator). For co-culture, peripheral blood mononuclear cells were isolated by density gradient centrifugation over Ficoll/Hypaque as described before (Andreesen et al., 1990 (36)). T cells were isolated by magnetic bead separation (human Pan T Cell Isolation Kit, Miltenyi Biotec, 130-096-535). T cells were cultured in RPMI 1640 (GIBCO, 31870-025), supplemented with 10% human AB serum (BRK, Bavarian Red Cross), 2 mM L-glutamine (PAN-Biotech, P04-80100), essential vitamins (GIBCO, 1112037) and non-essential amino acids (GIBCO, 11140035), 1 mM pyruvate (GIBCO, 11360039), b-mercapthoethanol (GIBCO, 31350010), 0.5% penicillin and streptomycin (both GIBCO, 15140122) and 25 IU/mL rhIL-2 (PeproTech, 200-02) in a humidified atmosphere (5% CO2, 95% air) at 37°C in a Heraeus incubator. 1x10^6^ cells T cells were seeded in 24-well plates with indicated treatments and stimulated with anti-CD3/CD28 Dynabeads (Thermo Fisher Scientific, 11132D) at a cell to bead ratio of 1:1. For co-culture with tumor spheroids, beads were removed after 48h of stimulation, T cells were washed and 0.1x10^6^ T cells in 100 µl tumor medium supplemented with 25 IU/mL Il-2 were added to each spheroid with indicated treatments. After 24h co-culture, they were washed and seeded with fresh medium for live cell imaging.

### 2.3 Flow cytometry

After 48h and 96h, T cells subsets were harvested, Dynabeads were removed and the cell suspensions were partitioned into FACS tubes. Cells were stained with Zombie Aqua Fixable Viability Kit (BioLegend, 423102) or Zombie NIR™ Fixable Viability Kit (BioLegend, 423106) and different surface antibodies PE anti-CD45RO (BD, 555493), PE anti-CD154 (BD 555700), PE anti-CD226 (BioLegend, 337106), PE anti-CD154 (BioLegend, 310806), PE anti-BTLA (BioLegend, 344505), APC anti-TIGIT (BioLegend, 372706), APC anti-CD25 (BD, 340907), APC anti-CD69 (BD, 555533), APC anti-CD62L (BD, 559772), APC anti-CD279 (PD-1) Antibody (BioLegend, 329908), APC anti-GITR (BioLegend, 371206), V450 anti-CD27 (BD, 561408), FITC anti-CD28 (BD, 555728), PE anti-CD39 (BD, 555464), FITC anti-CD44 (BioLegend, 397518), FITC anti-CD95 (BD, 561975), FITC anti-CD134 (OX40) (BioLegend, 350006), BV421 anti-CD137 (BioLegend, 309820), PE-Cy7 anti-CD223 (LAG-3) (eBioscience, 25-2239-42), PerCP-Cy5.5 anti-CD244 (BioLegend, 329515), PE-Cy7 anti-PD-L1 (BioLegend, 329717), PE-Cy7 anti-ICOS (BioLegend, 313519), PE-Cy7 anti-CD366 (Tim-3) (BioLegend, 345013), PE-Cy7 anti-Granzyme B (BioLegend, 396410), BV421 anti-CTLA-4 (Biolegend, 369606), BV421 anti-Perforin (BioLegend, 353307), APC anti-LDLR (R&D Systems, FAB2148A), APC anti-FOXP3 (eBioscience, 17-4776-42), Pacific Blue anti-CD8 (BD, 558207), BV510 anti-PD-1 (BioLegend, 329932), BV510 anti-CD3 (BioLegend, 317332), BV605 anti-CD3 (BioLegend, 317322), BV711 anti-CD4 (BD, 563028) following the manufacturer instructions. Flow cytometry data were acquired using the Fortessa System (BD) or Celesta System (BD). Data were analyzed using FlowJo (v10.8.1).

### 2.4 Single-cell metabolic assays

Cytosolic reactive oxygen species (ROS) were determined after surface marker staining by applying 10 µM 2′,7′-dichlorofluorescin diacetate (Sigma Aldrich, D6883) for 20 minutes in a cell culture incubator at 38°C in FACS wash buffer in air tight tubes. Cells were washed with 3 ml cold PBS, resuspended in FACS wash buffer and measured immediately.

Mitochondrial content was assessed by staining with MitoTracker Green FM (Thermo Fisher Scientific, M7514). Cells were incubated with 15 nM MitoTracker and 1.3 µM cyclosporine A in RPMI1640 supplemented with 2 mM L-Glutamine for 1 hour at 37°C in a cell culture incubator. Surface staining was performed afterwards.

Tetramethylrhodamine methyl ester (TMRM) (Thermo Fisher Scientific, T668) is a membrane-permeable, cationic, red-orange fluorescent dye that is enriched in active mitochondria. Cells were incubated with 10 µM TMRM and 1.3 µM cyclosporine A in RPMI1640 supplemented with 2 mM L-Glutamine for 30 minutes at 37°C in a cell culture incubator. Surface staining was performed afterwards.

Cholesterol was determined by Filipin staining after surface marker staining by adding 50 µg/ml Filipin in 500 µl PBS for 45 minutes at room temperature. Cells were washed with FACS wash buffer, resuspended, and measured immediately.

### 2.5 ELISA

After 48 hours and 96 hours, cell cultures were harvested, centrifuged (7 min, 1300 PRM (300g) at 4°C) and the supernatants were stored at -20°C. Supernatants were thawed and subsequently TNFα, INFg, IL-4, IL-7, IL-10, IL-15 and IL-17 concentrations were analyzed using the equivalent ELISA-kits (Human DuoSet ELISA R&D Systems, IFNg DY285B, TNF-alpha DY210, IL-4 DY204, IL-7 DY207, IL-10 DY217B, IL-15 DY247, IL-17 DY317). ELISAs were performed following the manufacturer instructions.

### 2.6 Monitoring of oxygen consumption and pH *in-vitro*

Cellular oxygen consumption and pH levels in culture medium were determined non-invasively by the PreSens technology (PreSens Precision Sensing GmbH). 0.8x10^6^ T cells with anti-CD3/CD28 Dynabeads (with a cell to bead ratio of 1:1, Thermo Fisher Scientific) were seeded in 24-well OxoDish^®^ OD24 plates without fixation in 1 mL medium under cell culture conditions for the indicated period of time. Data were analyzed using PreSens SDR_v38 software.

### 2.7 Real-time live cell imaging

After 24h co-culture with pre-activated T cells, spheroids were washed and transferred to a fresh 96-Well Poly-HEMA plate with 200 µl RPMI 1640 (GIBCO, 31870-025), 10% fetal calf serum (Sigma, F7524), 2 mM glutamine (PAN Biotech, P04-80100)) and 25 IU/ml IL-2, and labelled with 20 µl/ml Cyto3D™ Live-Dead Assay Kit dye (TheWell Bioscience, BM01). Plates were incubated in the Incucyte ZOOM live-cell imager (Essen Bioscience, Welwyn Garden City, UK) at 37°C and 5% CO2 and images were acquired (4x or 10x objective) at the indicated time points. Data were analyzed with the Incucyte ZOOM 2020B software (Essen Bioscience) by creating a threshold-based mask for the calculation of the green object total area (GOTA) of viable cells (green = viable, red = dead).

### 2.8 Statistical analysis

Depending on normal or non-normal distribution, RM one-way ANOVA with Geisser-Greenhouse correction and Dunnett`s multiple comparison test or Friedman test with Dunn`s multiple comparison test was performed. Significance was indicated as p < 0.05 *, p < 0.01 **, p < 0.001 *** referring to control. Data were corrected for multiple testing according to Benjamini and Hochberg as indicated (retrieved from https://statistikguru.de/rechner/adjustierung-des-alphaniveaus.html).

## 3 Results

### 3.1 Presence of LDL^high^ balances the metabolic activity in CD4^+^ and CD8^+^ T cells

Kishton et al. acknowledged effector T cells to exhibit an enhanced metabolic activity *in-vitro*, characterized by a high glycolytic activity and reactive oxygen species (ROS) production, resulting in a strong proliferation and cytokine production during expansion. However, upon *in-vivo* transfer, these cells showed a poor persistence and anti-tumor activity.

On the contrary, T cells exhibiting a balanced metabolic activity and a memory phenotype *in-vitro* were associated with a high anti-tumor activity and increased persistence *in-vivo* (37, 38). Furthermore, Gicobi et al. recently demonstrated that resilient T cells, which were resistant in a harsh tumor microenvironment and responsive to immunotherapy, compensated for excessive ROS to maintain metabolic fitness and preserve high cytotoxic capacity (39).

Therefore, we were intrigued to see, if the presence of LDL^high^ was linked to a balanced T cell metabolism.

**Figure 1:**
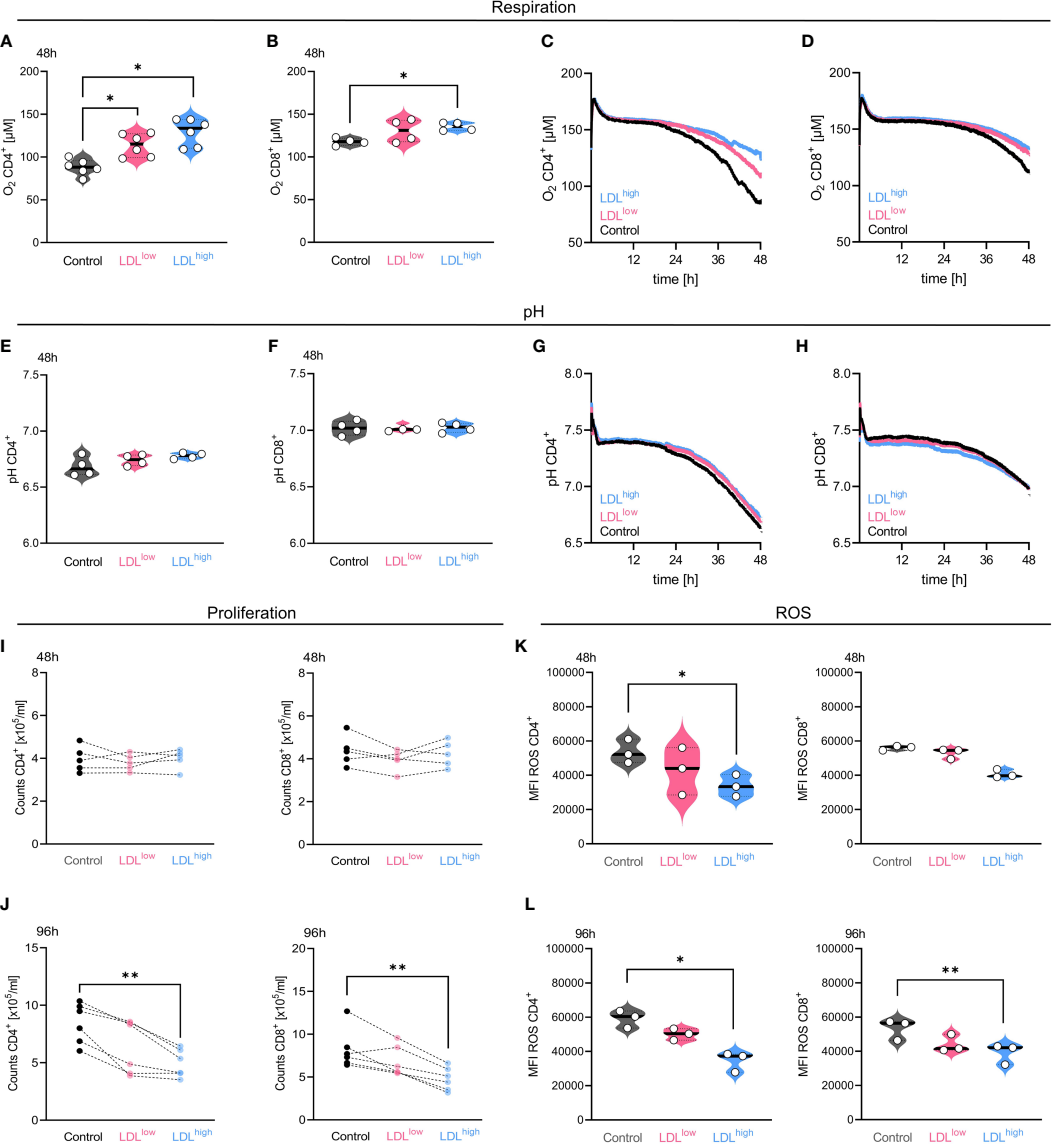
In the presence of LDL^high^, oxygen consumption, cell proliferation and reactive oxygen species (ROS) accumulation were significantly reduced in both T cell subsets. T cells were freshly isolated and stimulated with anti-CD3/CD28 beads, IL-2 and treated with different LDL dosages (LDL^low^ = 50 µg/ml and LDL^high^ = 200 µg/ml) versus medium control. *Respiration:* [**(A)** CD4^+^, **(B)** CD8^+^] LDL significantly down-regulated oxygen consumption after 48 h (CD4^+^ n = 6, CD8^+^ n = 4) in both T cell subsets. [**(C)** CD4^+^, **(D)** CD8^+^] Representative illustration of the detection of oxygen concentration over 48 h in both T cell subsets by PreSens technology. *pH*: [**(E)** CD4^+^, **(F)** CD8^+^] The CD4 subset showed a reduced acidification in the presence of LDL^high^ by trend, however no significance. [**(G)** CD4^+^, **(H)** CD8^+^] Representative illustration of the pH detection over 48 h in both T cell subsets by PreSens technology *Proliferation:* [**(I)** 48 h, **(J)** 96 h] Proliferation, shown as counts x 10^5^/ml, was significantly restrained in both T cell subsets by LDL^high^ after 96 h. *ROS*: [**(K)** 48 h, **(L)** 96 h]. Accumulation of ROS was significantly reduced after 48 h and 96 h by LDL^high^ in both T cell subsets (CD8^+^ 48 h not significant). Depending on normal or non-normal distribution, RM one-way ANOVA with Geisser-Greenhouse correction and Dunnett`s multiple comparison test or Friedman test with Dunn`s multiple comparison test was performed. Significance was indicated as p < 0.05 *, p < 0.01 **, p < 0.001 *** referring to control. Data were corrected for multiple testing according to Benjamini and Hochberg.

T cells were freshly isolated and stimulated with anti-CD3/CD28 beads, IL-2 and treated with different LDL dosages (LDL^low^ = 50 µg/ml and LDL^high^ = 200 µg/ml) versus medium control. We analyzed T cell proliferation, mitochondrial metabolism, intracellular cholesterol, acid production, respiration, and ROS accumulation (**Figure 1**, **Supplemental Data S3**, **6**, **8**, **9** statistics and data points).

T cell oxygen consumption was significantly reduced in the presence of LDL^high^, indicating a reduced turnover by oxidative phosphorylation. Furthermore, the CD4^+^ T cell subset showed less acidification by trend, however glycolytic activity was preserved (**Figures 1A–H**).

T cell proliferation was strongly impaired by LDL^high^ after 96 h, cell counts x 10^5^/ml hardly differed from pre-proliferation cell counts after 48 h (**Figures 1I, J**).

ROS accumulation was significantly reduced at both time points in both subsets (**Figures 1K, L**). However, we did not find any significant differences in the entire CD4^+^ and CD8^+^ T cell subset, respectively, concerning the mitochondrial mass, the mitochondrial membrane potential and intracellular cholesterol after 48 h and 96 h (**Supplemental Data 3** and **6**).

The synopsis of the findings provided strong indications for a balanced metabolism in the presence of LDL^high^ in both T cell subsets. As a balanced metabolic activity has been associated with a central memory phenotype, we investigated for a phenotypical shift in the T cell subsets (37).

### 3.2 LDL^high^ induces a central memory phenotype in the CD4^+^ T cell subset

Central memory T cells (T_CM_) have been shown to exhibit a superior persistence and anti-tumor immunity compared to effector memory T cells (T_EM_) (40). T_CM_ were associated with a favorable prognosis in oral squamous cell carcinoma and gastric cancer. Furthermore, a predominance of T_CM_ predicted response to checkpoint therapy in Merkel cell carcinoma (41–43).

CD4^+^ and CD8^+^ T cells were stimulated with activating beads and IL-2 in the presence of medium control, LDL 50µg/ml (LDL^low^) or LDL 200µg/ml (LDL^high^), respectively. Memory markers were analyzed after 48 h and 96 h.

**Figure 2:**
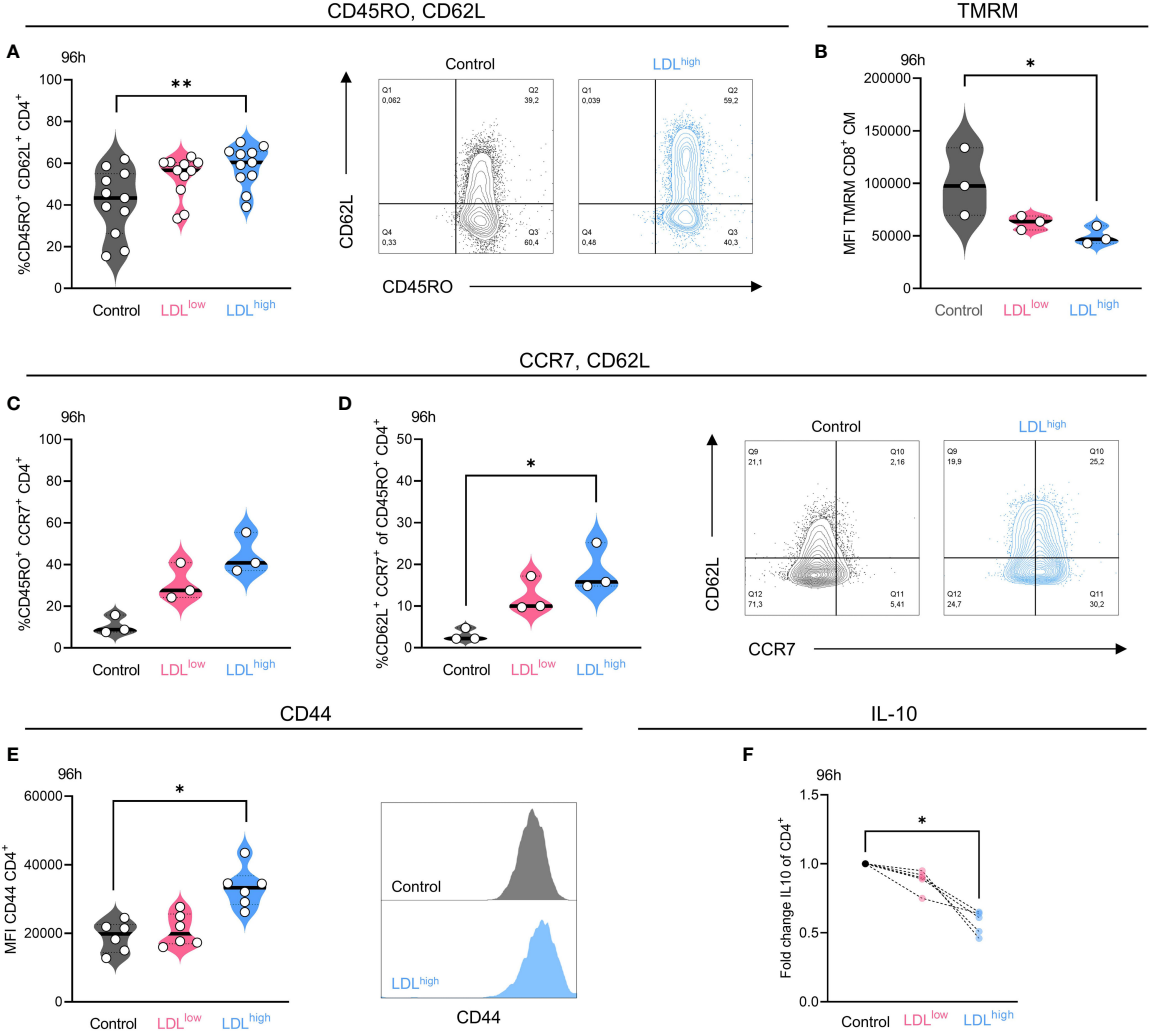
LDL^high^ significantly enhanced the fraction of CD4^+^ T cells with a central memory (T_CM_) phenotype after 96 h. CD4^+^ T cells were freshly isolated and stimulated with anti-CD3/CD28 beads, IL-2 and treated with different LDL dosages (LDL^low^ = 50 µg/ml and LDL^high^ = 200 µg/ml) versus medium control. **(A)** LDL^high^ significantly enhanced the fraction of CD45RO^+^/CD62L^+^ cells in the CD4^+^ T cell subset after 96 h. Representative FACS plots present the percentage of CD45RO^+^ and CD62L^+^ cells in the presence of medium control versus LDL^high^. **(B)** TMRM was reduced in CD8^+^ T cells with a central memory-like phenotype in the presence of LDL^high^. **(C,D)** Additional, preliminary experiment, demonstrating the upregulation of CCR7 **(C)** and the fraction of CCR7/CD62L double positive cells **(D)** in the presence of LDL 150 µg/ml in the CD4^+^/CD45RO^+^ subset. Representative FACS plots present the percentage of CCR7^+^ and CD62L^+^ cells in the presence of medium control versus LDL 150 µg/ml. **(E)** The expression of CD44 was significantly enhanced in the presence of LDL^high^. A representative FACS histogram illustrates the Mean Fluorescence Intensity (MFI) for CD44. **(F)** The secretion of IL-10 was significantly reduced in the presence of LDL^high^. ELISA data (pg/ml) normalized to control. Depending on normal or non-normal distribution, RM on-way ANOVA with Geisser-Greenhouse correction and Dunnett`s multiple comparison test or Friedman test with Dunn`s multiple comparison test was performed. Significance was indicated as p < 0.05 *, p < 0.01 ** referring to control. Data were corrected for multiple testing according to Benjamini and Hochberg if indicated..

CD4^+^ T cells shifted toward CD45RO^+^ CD62L^+^ central memory phenotype in the presence of LDL^high^ after 96 h (**Figure 2A**).

We also observed a trend towards a T_CM_ phenotype in the CD8^+^ subset after 96 h, however the data were not significant. No effects were seen after 48 h.

A preliminary experiment also revealed the up-regulation of CD45RO^+^ CCR7^+^ cells in the CD4^+^ T cell subset by LDL (**Figure 2C**), however there was only a limited fraction of CD62L/CCR7 double positive cells (**Figure 2D**), which may be traced back to a mixed memory phenotype and alternatively shedding of CD62L (44, 45).

Furthermore, completing the phenotype, the expression of CD44 was significantly enhanced by LDL^high^ in the CD4^+^ subset after 96 h (**Figure 2E**). Besides its function as an activation marker and high expression on memory cells, CD44 can promote survival and memory cell development in Th1 cells (46). No effects were seen regarding the expression of CD27, CD28 and FOXP3. T cells were mostly positive for CD27 and CD28, and FOXP3 was barely expressed under all conditions (**Supplemental Data S1**–**5**: FACS gating, statistics and data points).

In a further step, we investigated the impact of LDL on memory cell-modulating cytokines. IL-21 has been associated with the induction of a central memory phenotype (47, 48) and IL-7 and IL-15 have been linked to maintenance of a long-term memory survival (49). Autocrine production of IL-7 and IL-15 has been reported (50, 51). Moreover, IL-10 has been linked to suppression of memory development and memory cell responses (52, 53). We could not find any evidence for memory cell induction and maintenance by IL-21, IL-7 and IL-15. Secretion of IL-21 was downregulated and IL-7 and IL-15 could not be detected in the presence of LDL^high^.

However, secretion of IL-10 was significantly impaired by LDL^high^, possibly thereby enabling memory formation (**Figure 2F**, **Supplemental Data S6**, **7**).

Besides downregulation of ROS and shift towards of a central memory phenotype, the mitochondrial membrane potential has been acknowledged to identify cells with a balanced metabolism and an enhanced stemness for cellular therapy (54). Subtyping for T_CM_ phenotype after 96 h revealed a significant reduction of the mitochondrial membrane potential in the CD8^+^ subset (**Figure 2B**), perhaps indicating resilient T cells with a high cytotoxic capacity (39).

In a further step we also investigated phenotypical changes concerning exhaustion, activation, and co-stimulatory surface markers (**Table 1**).

**Table 1:** Summary of all surface markers analyzed on CD4^+^ and CD8^+^ T cells after stimulation for 48 h and 96 h.

Surface Markers:	Checkpoint	Costimulatory	Activation	Others
	CD39	CD27	CD25	CD45RO/CD62L and CD44
	CD244	CD28	CD69	FOXP3
	CTLA-4	CD137		CD95
	LAG-3	CD154		LDLR
	PD-1	CD226		
	PD-L1	GITR		
	TIGIT	ICOS		
	TIM-3	OX40		
	BTLA			

These markers and their functions were considered potentially relevant in the context of immunotherapy.

### 3.3 Presence of LDL^high^ is associated with a less exhausted phenotype in CD4^+^ and CD8^+^ T cells besides upregulation of the co-stimulatory marker CD154 (CD40L) in the CD4^+^ T cell subset

CD4^+^ and CD8^+^ T cells were stimulated with activating beads and IL-2 in the presence of medium control, LDL 50µg/ml (LDL^low^) or LDL 200 µg/ml (LDL^high^), respectively. Surface checkpoint markers, costimulatory markers and activation markers were analyzed after 48 h and 96 h.

**Figure 3:**
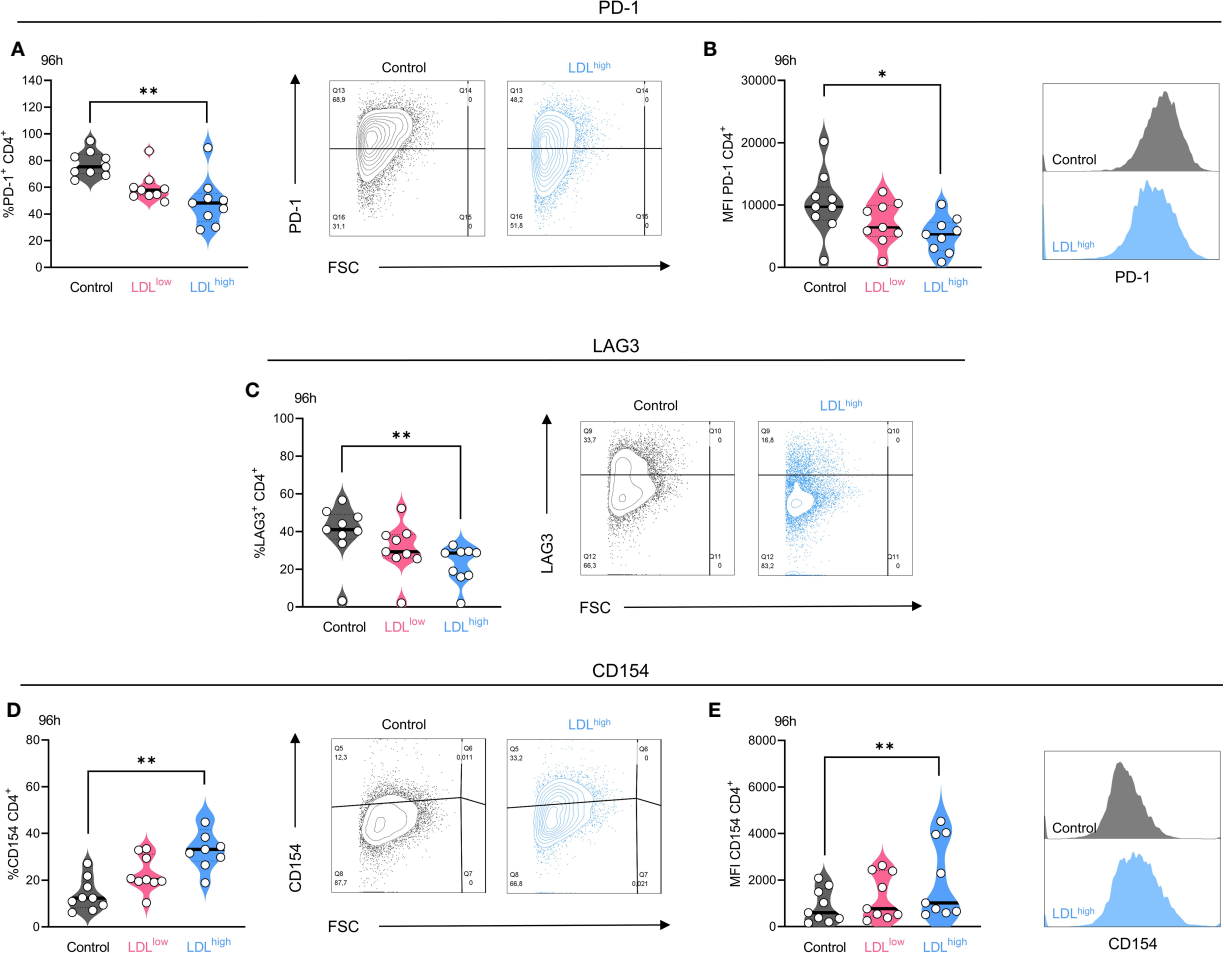
The exhaustion markers PD-1 and LAG-3 were down-regulated in the presence of LDL^high^ (here shown for the CD4^+^ subset after 96 h) and the costimulatory marker CD154 (CD40L) was upregulated in the CD4^+^ T cell subset. T cells were freshly isolated and stimulated with anti-CD3/CD28 beads, IL-2 and treated with different LDL dosages (LDL^low^ = 50 µg/ml and LDL^high^ = 200 µg/ml) versus medium control. *PD-1:* LDL^high^ significantly reduced the fraction **(A)** of PD-1^+^ cells and the expression **(B)** of PD-1 on the CD4^+^ T cell subset after 96 h. Representative FACS plots and histograms are presented (A,B). *LAG-3*: LDL^high^ significantly reduced the fraction of LAG-3^+^ cells in the CD4^+^ T cell subset after 96 h. Representative FACS plots are presented **(C)**. *CD154*: The fraction of CD154 (CD40L) positive cells **(D)** and the expression **(E)** of CD154 was significantly enhanced in the presence of LDL^high^ in the CD4^+^ subset. Representative FACS plots and histograms are presented (D,E). Depending on normal or non-normal distribution, RM one-way ANOVA with Geisser-Greenhouse correction and Dunnett`s multiple comparison test or Friedman test with Dunn`s multiple comparison test was performed. Significance was indicated as p < 0.05 *, p < 0.01 ** referring to control. All data were corrected for multiple testing according to Benjamini and Hochberg.

In the group of checkpoint markers, LDL^high^ induced a significant down-regulation of the fraction (%) of PD-1 positive cells in both T cell subsets after 48 h and in the CD4^+^ subset also after 96 h (**Figure 3A**).

The expression (MFI) of PD-1 was significantly reduced in the CD4^+^ subset after 48 h and in both subsets after 96 h (**Figure 3B**). The fraction (%) of LAG-3^+^ T cells was significantly reduced in both T cell subsets after 96 h (**Figure 3C**), the expression (MFI) of LAG-3 was reduced temporarily in the CD4^+^ subset after 48h. High expression of PD-1 and LAG-3 have been shown to be associated with a loss of T cell function (55–58). Downregulation of these exhaustion and suppression markers, especially LAG-3, may enhance the efficacy of PD-1 blockade (59–61).

Intriguingly, in the presence of LDL^low^, but not LDL^high^, PD-L1 was significantly up-regulated in the CD4^+^ subset after 48 h and 96 h. Although the expression of PD-L1 on CD4^+^ T cells was associated with an improved PFS in NSCLC patients (62), PD-L1 signaling on human memory CD4^+^ T cells induced a regulatory phenotype (63). The expression of all other checkpoint markers was not significantly affected by LDL (**Supplemental Data S1**–**5**).

In the group of co-stimulatory markers the fraction and expression of CD154 (CD40L) was significantly up-regulated in the CD4^+^ T cell subset after 96 h (**Figures 3D, E**). Interaction of CD154 with CD40 has been demonstrated to mediate anti-tumoral immune responses by enhancing the immunogenic cell death of tumor cells, activation of antigen presenting cells, production of proinflammatory factors, co-stimulation of CD4^+^ and CD8^+^ T cells, and the tumor cell susceptibility to T cell lysis (64, 65).

After 48 h, OX40^+^ T cells were temporarily reduced in the CD4^+^ subset under both conditions containing LDL, however no differences were seen after 96 h. The expression of all other co-stimulatory markers was not significantly affected by LDL. Furthermore, in the group of activation markers and other markers, expression of CD25 and the LDL receptor were temporarily impaired and CD95 temporarily up-regulated under both conditions containing LDL referring to control after 48 h. No differences were seen after 96 h (**Supplemental Data S1**–**5**).

As the T cells exhibited a less exhausted phenotype, we were also intrigued to investigate cytokine secretion. In monoculture, we could not reveal any significant differences for IFNg, TNFa, granzyme B, IL-17 and IL-4, however the production of perforin was enhanced in both T cell subsets by trend (**Supplemental Data S3**, **6**, **7**).

To complete our understanding of the phenotypical und functional alterations induced by LDL, we investigated in a further step functional properties in a spheroid model.

### 3.4 LDL^high^ augments checkpoint blockade in a tumor spheroid co-culture model

**Figure 4:**
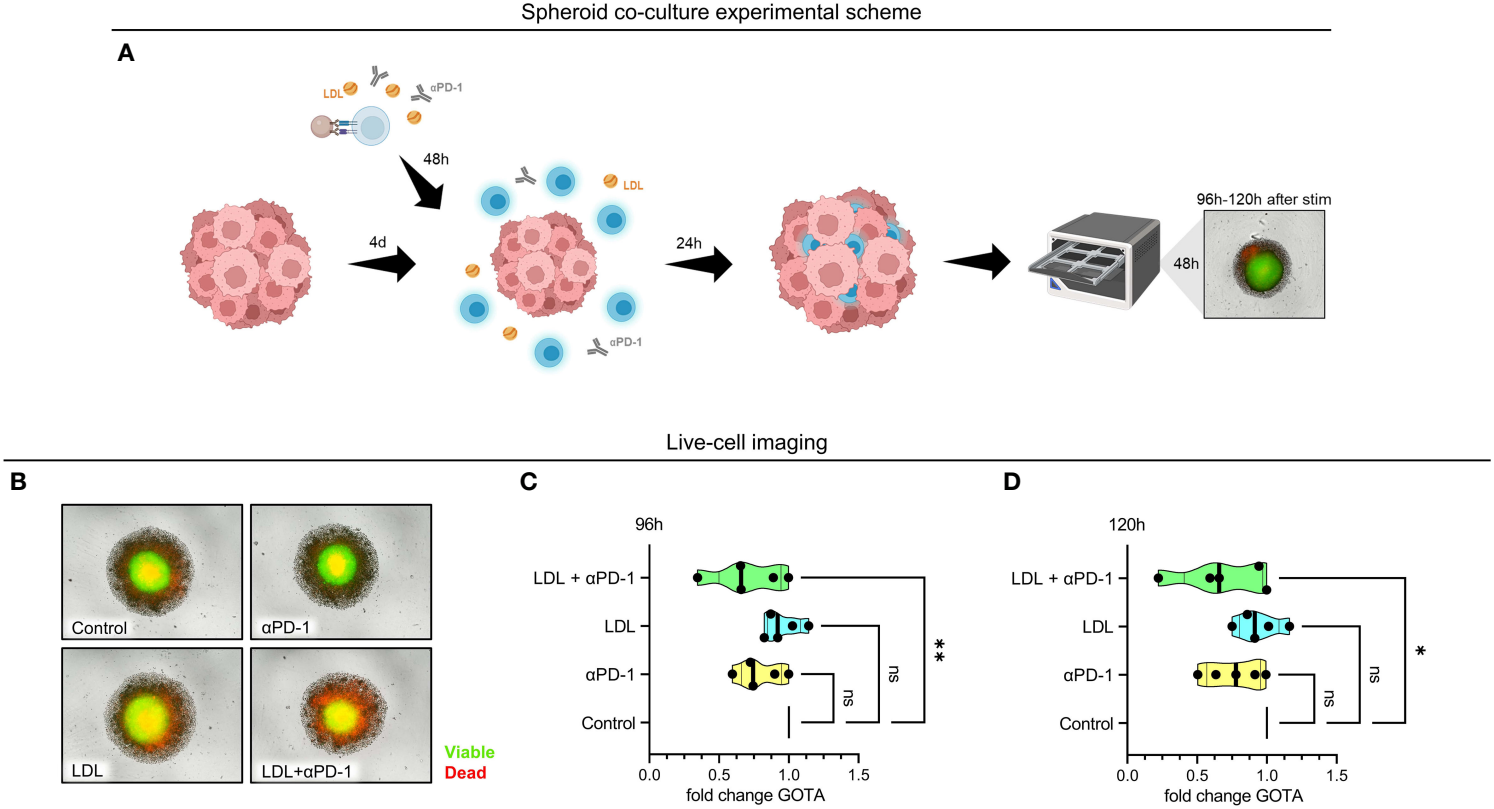
Referring to control, viable cells were significantly reduced in the HCT116 spheroid tumor model in the presence of LDL^high^ in combination with an anti-PD-1 antibody. Tumor spheroids were generated for 4 days with HCT116 colon carcinoma cells. T cells were freshly isolated and stimulated with anti-CD3/CD28 beads, IL-2 and treated with anti-PD-1, LDL^high^ or LDL^high^ + anti-PD-1 versus medium control. After 48 h, T cells were added to the tumor spheroid and co-cultured for further 24 h. For Incucyte life cell imaging system, spheroids were washed, stained with a viability dye and fluorescence was then monitored under culture conditions for further 24 h – 48 h (cumulative T cell stimulation time 96 h – 120 h). **(A)** Spheroid co-culture experimental scheme. Created with BioRender.com. **(B)** Representative picture of spheroids after 24 h live cell imaging. Red = dead, green = viable. **(C, D)** Quantification of the green object total area (GOTA), which determines viable cells after 24 h **(C)** and 48 h live cell imaging **(D)**. Depending on normal or non-normal distribution, RM one-way ANOVA with Geisser-Greenhouse correction and Dunnett`s multiple comparison test or Friedman test with Dunn`s multiple comparison test was performed. Significance was indicated as p < 0.05 *, p < 0.01 ** referring to control. ns, not significant.

Spheroids were generated for 4 days. In parallel, T cells were pre-activated with anti-CD3/CD28 beads and IL-2. After 2 days of stimulation with anti-PD-1, LDL^high^ or LDL^high^ + anti-PD-1 versus medium control, T cells were added to the spheroids and allowed to infiltrate for 24 h. Subsequently, the co-cultured spheroids were stained with a viability dye (red = dead, green = viable). Fluorescence was monitored for further 48 h (**Figure 4**, **Supplemental Data S10**, **Video S1**).

We did not find any significant differences concerning viability subsequently to the sole addition of an anti-PD-1 antibody or LDL^high^ in comparison to medium control. However, combination of LDL200µg/ml with an anti-PD-1 antibody induced a significant reduction of the normalized spheroid green object total area (GOTA, representing viable cells) compared to medium control after 24 h and 48 h (**Figures 4C, D**; n = 5).

## 4 Discussion

Recently, a debate has been launched on the impact of cholesterol in anti-PD-1 immunotherapy. As the tumor environment is mostly acidic, hypoxic, and glucose-deficient, lipids remain as an important source of energy for tumor cells and immune cells. However, lipid metabolism is exhibiting contradictory roles in tumor immune response and besides other lipids, cholesterol emerges as a double-edged sword in tumor immunity (66). In the context of cholesterol and immunotherapy, an association with response (3–6) to therapy versus treatment failure (7, 8) was delineated. Other authors interpreted the chain of causation differently and discussed chronic inflammation in first place, inducing T cell exhaustion, thus leading to cancer and hypercholesteremia as part of the metabolic syndrome, the latter again enhancing T cell exhaustion in the sense of a vicious circle (67).

So, being confronted with very paradox findings in complex environments, we aimed to straightforwardly investigate the effects of LDL on T cell subsets. We focused on LDL, since LDL emerged as the most significant serum lipid associated with response to immunotherapy in our and another preceding study (6, 33).

The LDL dosages for treatment referring to medium control were chosen based on LDL serum levels and their estimated tissue levels in responders (LDL^high^) and non-responders (LDL^low^) to anti-PD-1 checkpoint therapy (6, 34). *In-vitro*, we observed the enhancement of a central memory phenotype, downregulation of IL-10 secretion and up-regulation of CD40L in the CD4^+^ T cell subset. A balanced metabolism, indicated by lowered ROS levels, a preserved glycolytic flux, and a less exhausted phenotype under T cell activation were acknowledged in both T cell subsets, however a significant downregulation of the fraction of both, PD-1 and LAG-3 after 96 h, was only observed in the CD4^+^ subset. All potentially beneficial effects were merely significant (or more pronounced) in presence of LDL^high^. T cell transfer into a colorectal cancer HCT116 spheroid model revealed a significant reduction of the spheroid viability in presence of LDL^high^ plus anti-PD-1.

Zuzao et al. have shown that functioning CD4 immunity is essential for response to anti-PD-1 checkpoint therapy. Patients with a high proportion of CD4^+^ T cells with a central memory phenotype and a low PD-1/LAG-3 co-expression, were responsive to immunotherapy and moreover, a functional CD4 immunity supported the recovery of CD8 immunity, by, amongst others, secreting IFNg and priming dendritic cells *via* CD40L (59, 68). These findings have been confirmed by further studies, also considering tumor infiltrating T_CM_ and T_CM_ related genes (41–43). The phenotype described by Zuzao et al. is nearly identical to the effects we have seen, however under our conditions (presence of LDL and stimulation) the T cells were mostly positive for CD27 and CD28 as also described by Liu et al. (40). Furthermore, Zuzao et al. did not describe the expression of CCR7 or CD44.

Besides IL-7 and IL-21, IL-15 is one of the commonly known memory inducing cytokines. Interestingly, during CAR T cell development, addition of merely IL-15 enhanced similar beneficial effects, amongst others reduction of exhaustion, the preservation of a less differentiated memory cell phenotype and a superior anti-tumor response *in-vivo* (69). Analysis of memory inducing cytokines was negative in our experimental setting, however further investigation of the LDL induced signaling cascade might be expedient and moreover, the strongly impaired secretion of IL-10 may enable memory phenotype formation.

Moderate levels of ROS, generated from mitochondria and NADPH oxidases were shown to be crucial for T cell signaling, however excess amounts of ROS resulted in mutation and cell damage and were furthermore associated with T cell exhaustion and immunosuppression in the tumor milieu. Cellular anti-oxidants have been reported to be essential for maintaining anti-tumor immunity. T_CM_ express higher anti-oxidant levels than T_EM_, enabling an enhanced control of tumor growth (70–72). In presence of LDL^high^ we observed significantly reduced ROS levels, however maybe also due to the moderated oxygen consumption and presumably decreased oxidative phosphorylation (OXPHOS). A more resilient, less exhausted phenotype and cytotoxic capacity of T cells have been shown to be determined by balancing ROS (39).

However, observing further metabolic features of central memory induction, we were not able to detect a significantly enhanced mitochondrial mass or a lower mitochondrial membrane potential in the treated T cell populations, maybe due to incubation time or alternatively to culture conditions. Merely subtyping CD8+ T cells for a CM-like phenotype revealed a significantly reduced mitochondrial membrane potential after 96 h. Further research should be conducted, to define the spare respiratory capacity and the role of OXPHOS and fatty acid oxidation (FAO) in LDL treated T cells (37, 73). Nevertheless, the state of the LDL^high^ treated T cells induced a superior anti-tumor effect in the HCT116 spheroid model.

Mechanisms, how CD4^+^ T cells can contribute to anti-tumor immunity have been described. Growth arrest of cancer cells can be achieved by inducing senescence through cytokines like IFNg. Furthermore, CD4^+^ T cells can induce direct cytotoxicity in MHC II expressing tumor cells (74). Similarly, CD40L can develop cytotoxic effects *via* CD40 (75). Cytotoxicity *via* CD40 could be an imaginable mechanism in this model, as HCT116 has been shown to express CD40 (76) and the CD4^+^ subset significantly up-regulated CD40L.

Upregulation of CD154 has already been acknowledged on platelets in hypercholesteremia (77). Familiar functions associated with CD154 are of anti-tumorigenic nature, ranging from stimulation of antigen presenting cells, activation of immune effector cells, favorable modulation of the tumor environment, enhancement of the immunogenicity of malignant cells, besides the already mentioned direct action against tumor cells by inducing their apoptosis. Furthermore, the CD40-CD40 ligand pathway plays a critical role during rescue of exhausted CD8 T cells (78). Stimulation of this pathway is under consideration for immunotherapy (79). However, as a ligand to newly identified integrins, CD154 may also play a role in cancer pathogenesis, which may be one of the reasons for paradox effects seen with high cholesterol levels in immunotherapy (80).

Furthermore, concerning the paradox effects of cholesterol in literature, LDL also induces potentially inhibitory markers on the T cell subsets and depending upon ligand or receptor expression in the tumor milieu they may have an immunosuppressive effect, or be of no consequence. A significant up-regulation of PD-L1 was identified on the CD4^+^ subset under the LDL^low^ condition, however also by trend in the presence of LDL^high^. As already mentioned, PD-L1 signaling on CD4^+^ memory cells by cross-linking was demonstrated to evoke highly suppressive cells (63), however the expression of PD-L1 on immune cells can on the other hand be predictive of response in some tumor entities (81). For instance, patients with a higher proportion of PD-L1^+^ T cells at baseline had an improved objective response to PD-1 inhibitor therapy in melanoma and lung cancer (82).

Also, TIGIT was up-regulated in both T cell subsets in the presence of LDL^high^ by trend. Although the upregulation of TIGIT can exert immunosuppressive features in tumor immunity (83, 84), literature revealed TIGIT^+^ CD8^+^ subsets with cytotoxic properties (85).

## 5 Conclusions

In this study we showed that LDL skewed human CD4^+^ T cells into a memory phenotype, balanced T cell metabolism and reduced exhaustion marker expression in both subsets besides inducing the up-regulation of the co-stimulatory marker CD40L in the CD4^+^ subset. The changes resulted in an enhanced anti-tumor response in a HCT116 spheroid model under combination therapy with LDL^high^ and an anti-PD-1 antibody.

Further research should be conducted to achieve more understanding regarding changes in T cell metabolism and cell signaling by LDL. Moreover, also the effect of LDL on other lymphocyte populations and myeloid cells needs to be unraveled, in order to sufficiently optimize immunotherapy and adoptive cell transfer. Finally, also the effect of HDL on T cell function and metabolism in immunotherapy has not been understood and needs to be investigated.

## Institutional Review Board Statement

Buffy coats from healthy donors were obtained from the Department of Transfusion Medicine (University Hospital Regensburg) in form of remnants from routine platelet donations. The donations were approved by the Institutional Ethics Committee of the University of Regensburg (vote number 13-101-0240; 13-101-0238) and are in accordance with the Declaration of Helsinki.

## Data availability statement

The original contributions presented in the study are included in the article/**Supplementary Material**. Further inquiries can be directed to the corresponding author.

## Author contributions

Conceptualization: AS and MK. Formal analysis: AS, NB, JH, AM, RM, MR. Investigation: JH, NB, AS, CM, FV, PS, KR. Resources: MK, WH, PB. writing—original draft preparation: AS. writing—review and editing: all authors. All authors contributed to the article and approved the submitted version.

## Supplementary material

Supplementary Data Sheet 1 FACS gating MACS isolated CD4^+^ or CD8^+^ T cells

Supplementary Data Sheet 2 Original Surface Marker Statistics (not corrected)

Supplementary Data Sheet 3 Corrected Statistics Benjamini Hochberg

Supplementary Data Sheet 4 T cell Data Points 48h

Supplementary Data Sheet 5 T cell Data Points 96 h

Supplementary Data Sheet 6 ELISA graphs, Perforin, Granzym B, PRESENS Statistics and Metabolic Statistics (not corrected)

Supplementary Data Sheet 7 ELISA DATA

Supplementary Data Sheet 8 PRESENS Data Points

Supplementary Data Sheet 9 Original Viability and Proliferation Statistics (not corrected)

Supplementary Data Sheet 10 Spheroid Statistics Green Object Total Area

Supplementary Video 1 Incucyte Live Cell Imaging over 48 h.
