# Supplementary material for: Low-density lipoprotein balances T cell metabolism and enhances response to anti-PD-1 blockade in a HCT116 spheroid model
Source: Front Oncol. 2023 Jan 27;13:1107484. doi: 10.3389/fonc.2023.1107484 (PMC9911890; doi:10.3389/fonc.2023.1107484)
Supplement: Supplementary Data Sheet 1 — FACS gating MACS isolated CD4+ or CD8+ T cells [file DataSheet_1.zip › Supplement 1/Supplemental Data S1 FACS gating MACS isolated CD4+ or CD8+ T cells.PPTX]

## Slide 1
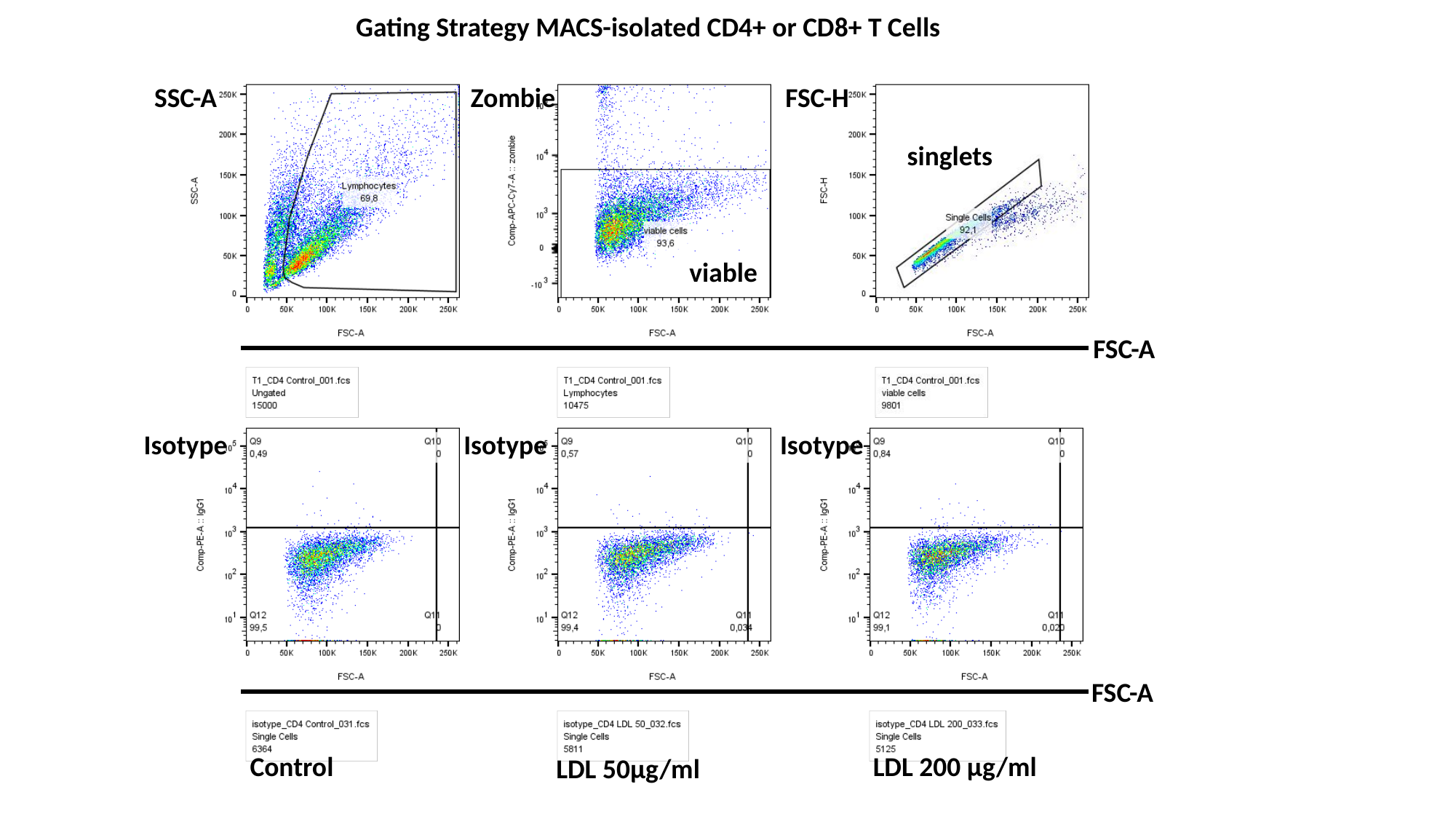

Gating Strategy MACS-isolated CD4+ or CD8+ T Cells
SSC-A
Zombie
FSC-H
singlets
viable
FSC-A
Isotype
Isotype
Isotype
FSC-A
LDL 200 µg/ml
Control
LDL 50µg/ml

## Slide 2
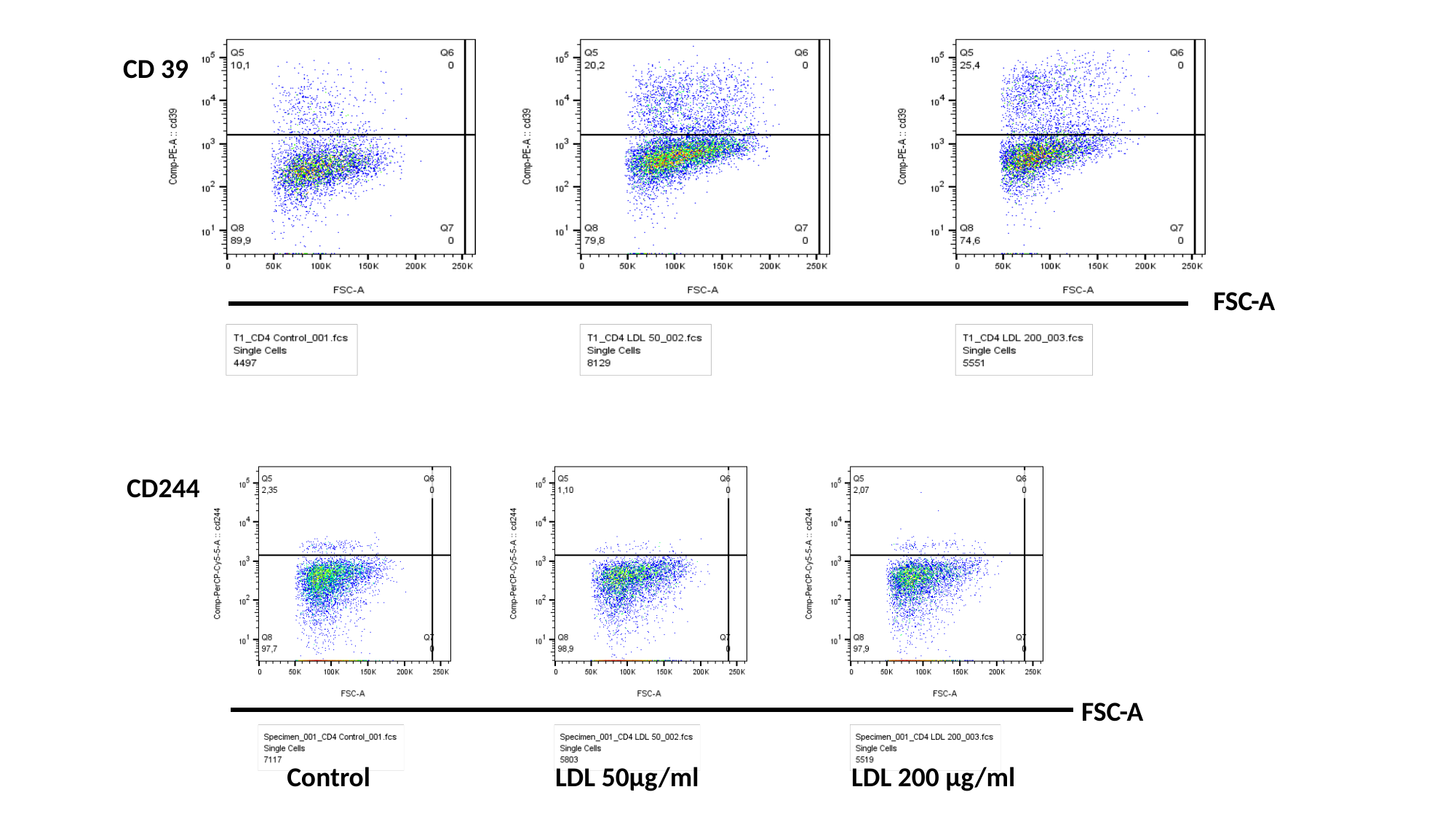

CD 39
FSC-A
CD244
FSC-A
Control
LDL 50µg/ml
LDL 200 µg/ml

## Slide 3
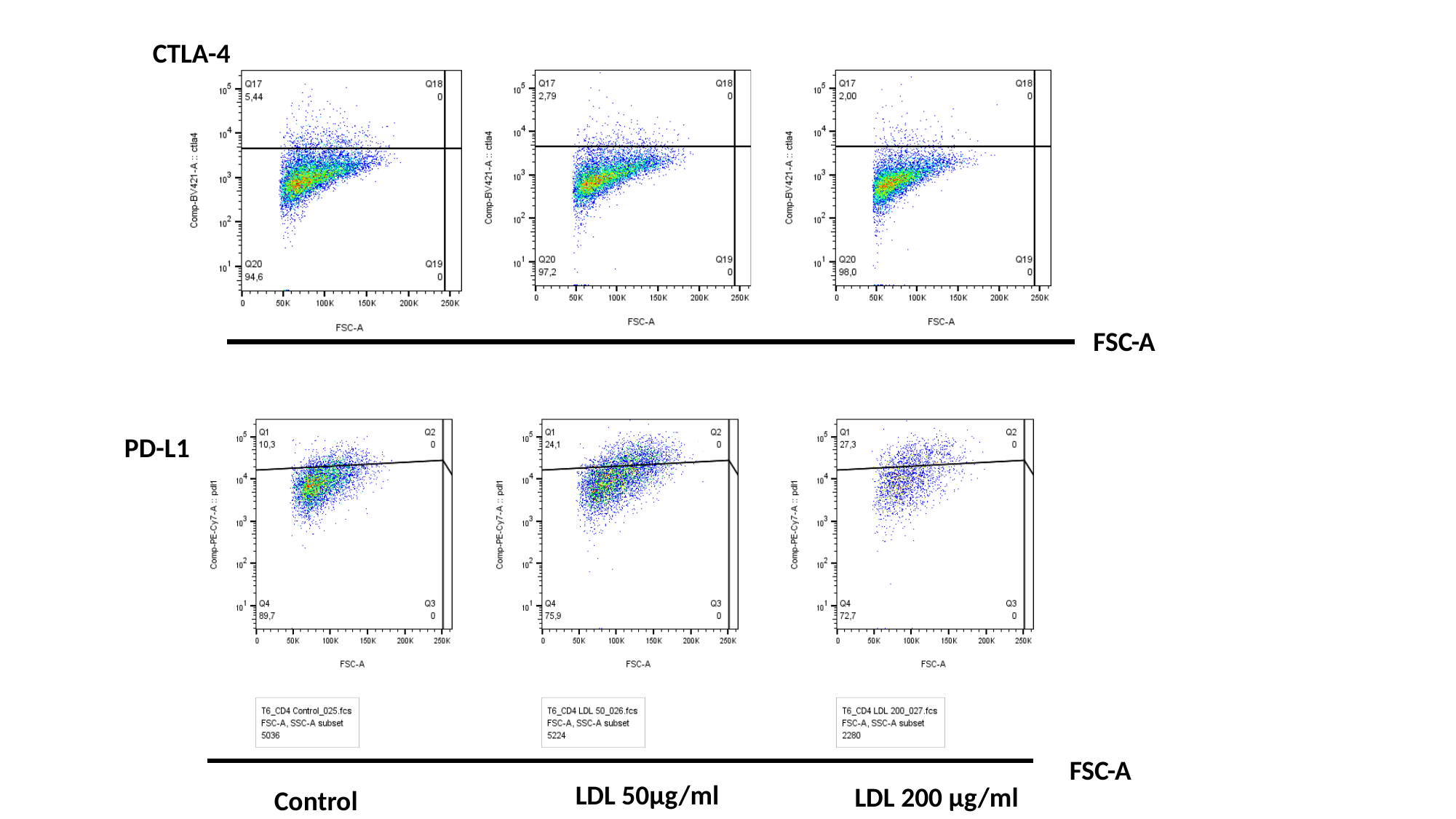

CTLA-4
FSC-A
PD-L1
FSC-A
LDL 50µg/ml
LDL 200 µg/ml
Control

## Slide 4
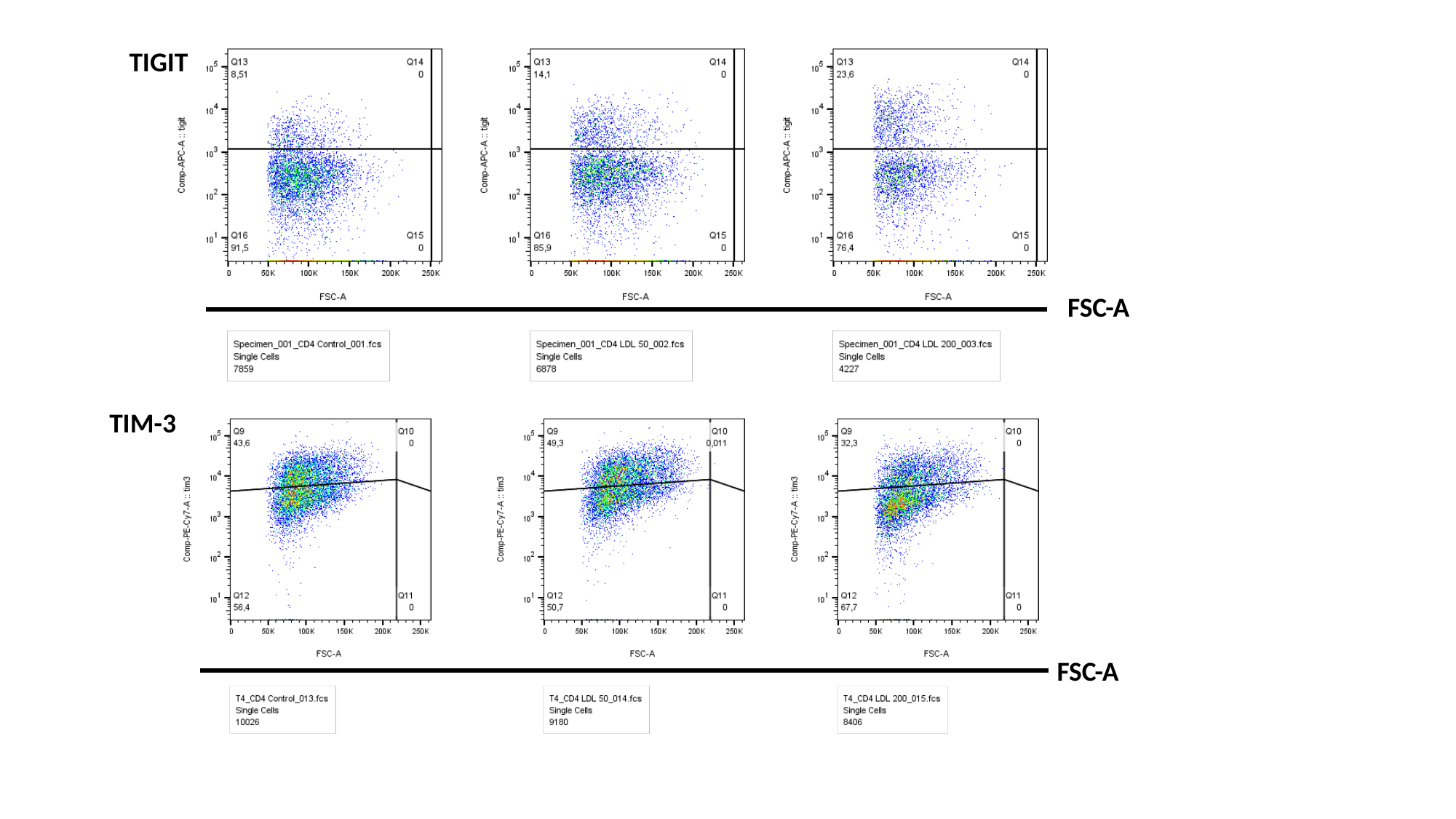

TIGIT
FSC-A
TIM-3
FSC-A

## Slide 5
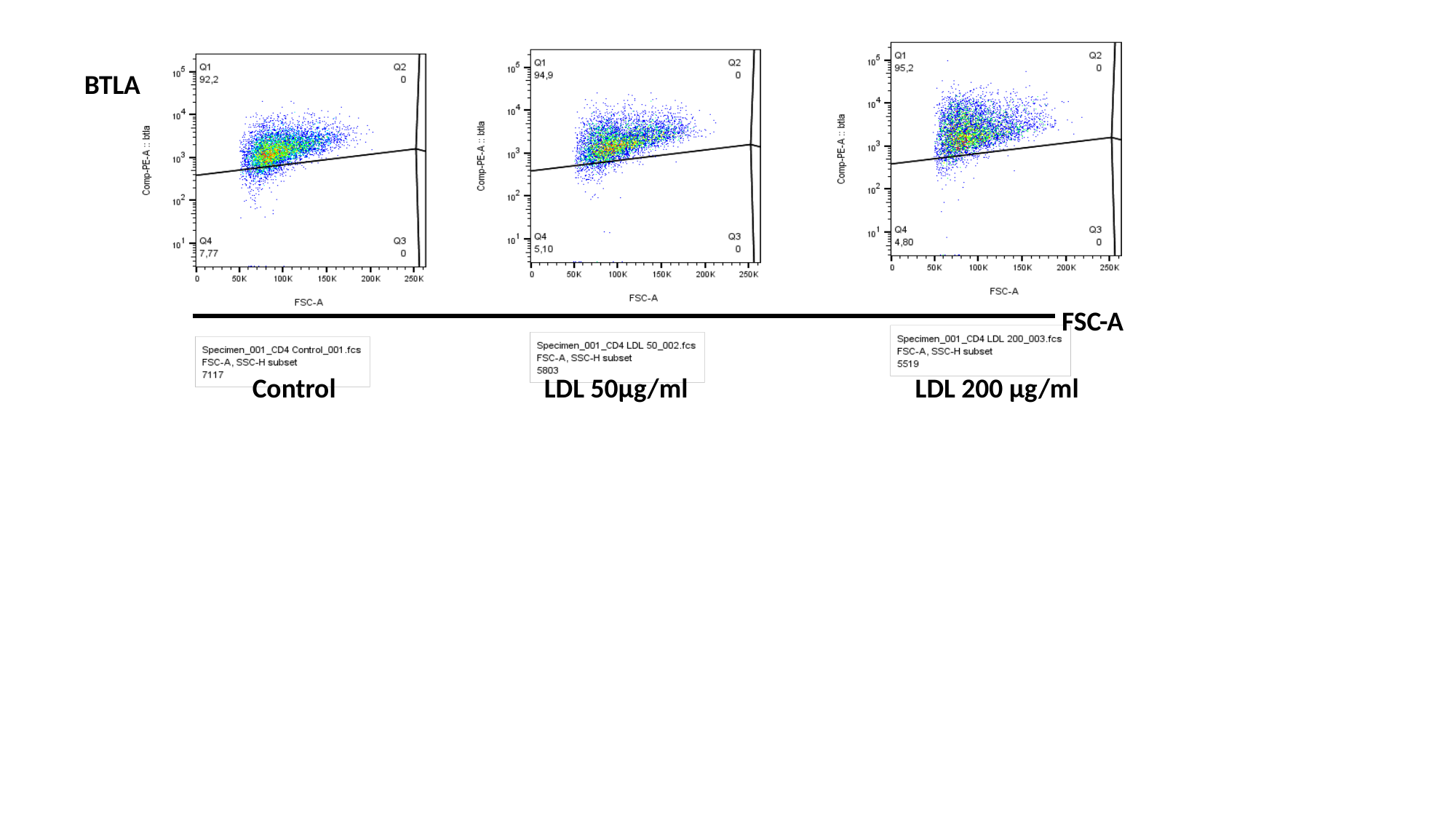

BTLA
FSC-A
Control
LDL 50µg/ml
LDL 200 µg/ml

## Slide 6
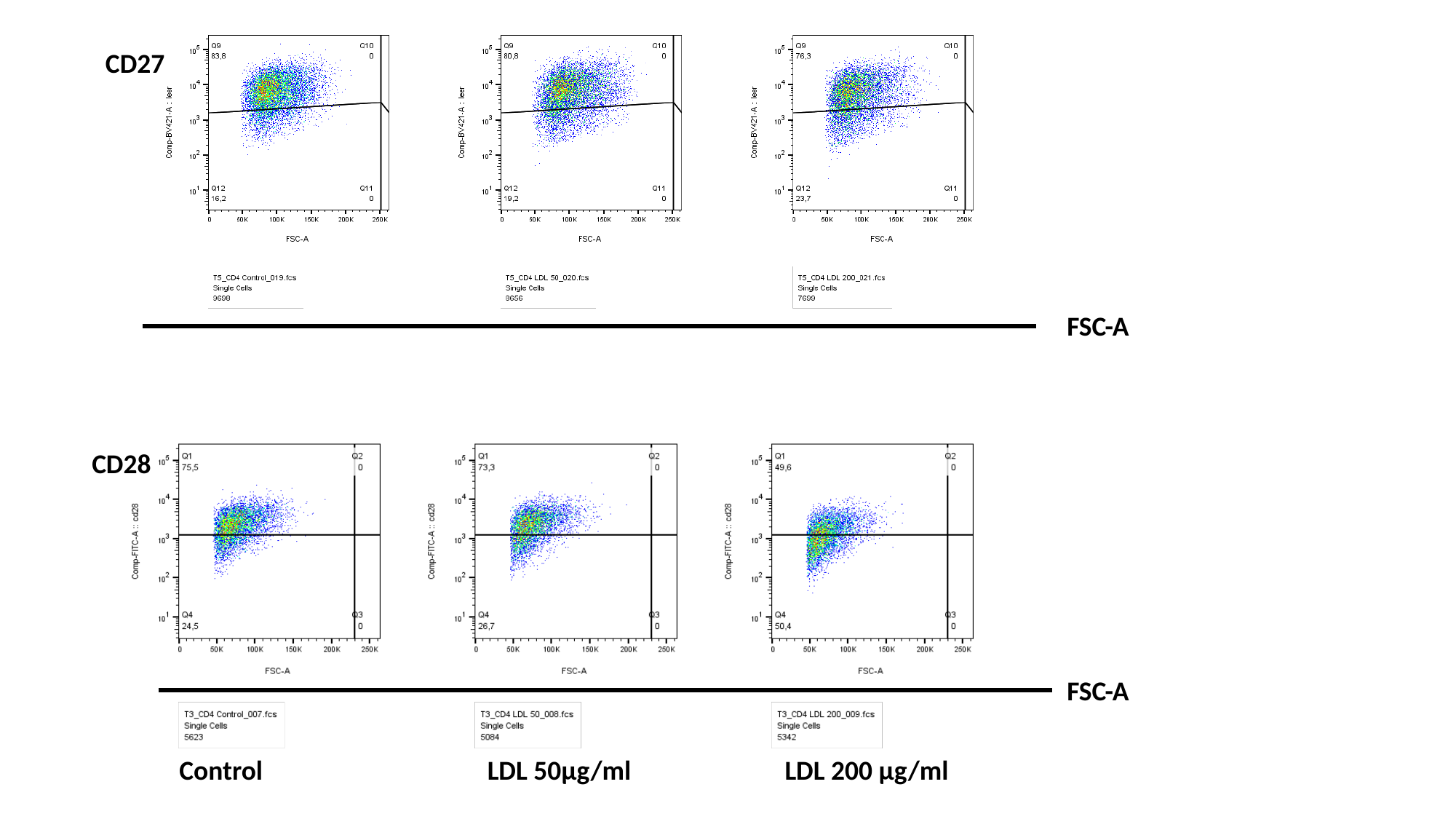

CD27
FSC-A
CD28
FSC-A
Control
LDL 50µg/ml
LDL 200 µg/ml

## Slide 7
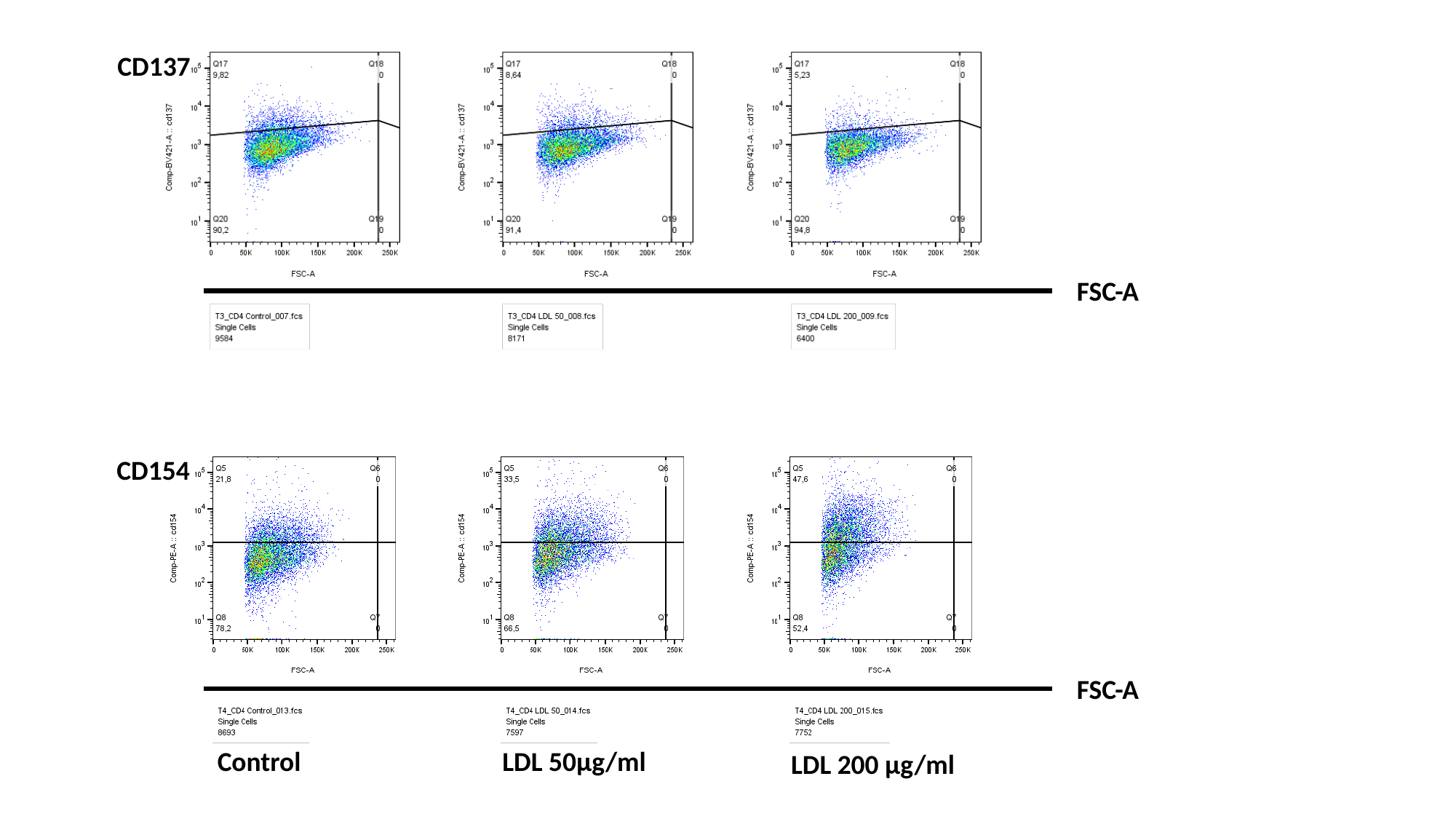

CD137
FSC-A
CD154
FSC-A
Control
LDL 50µg/ml
LDL 200 µg/ml

## Slide 8
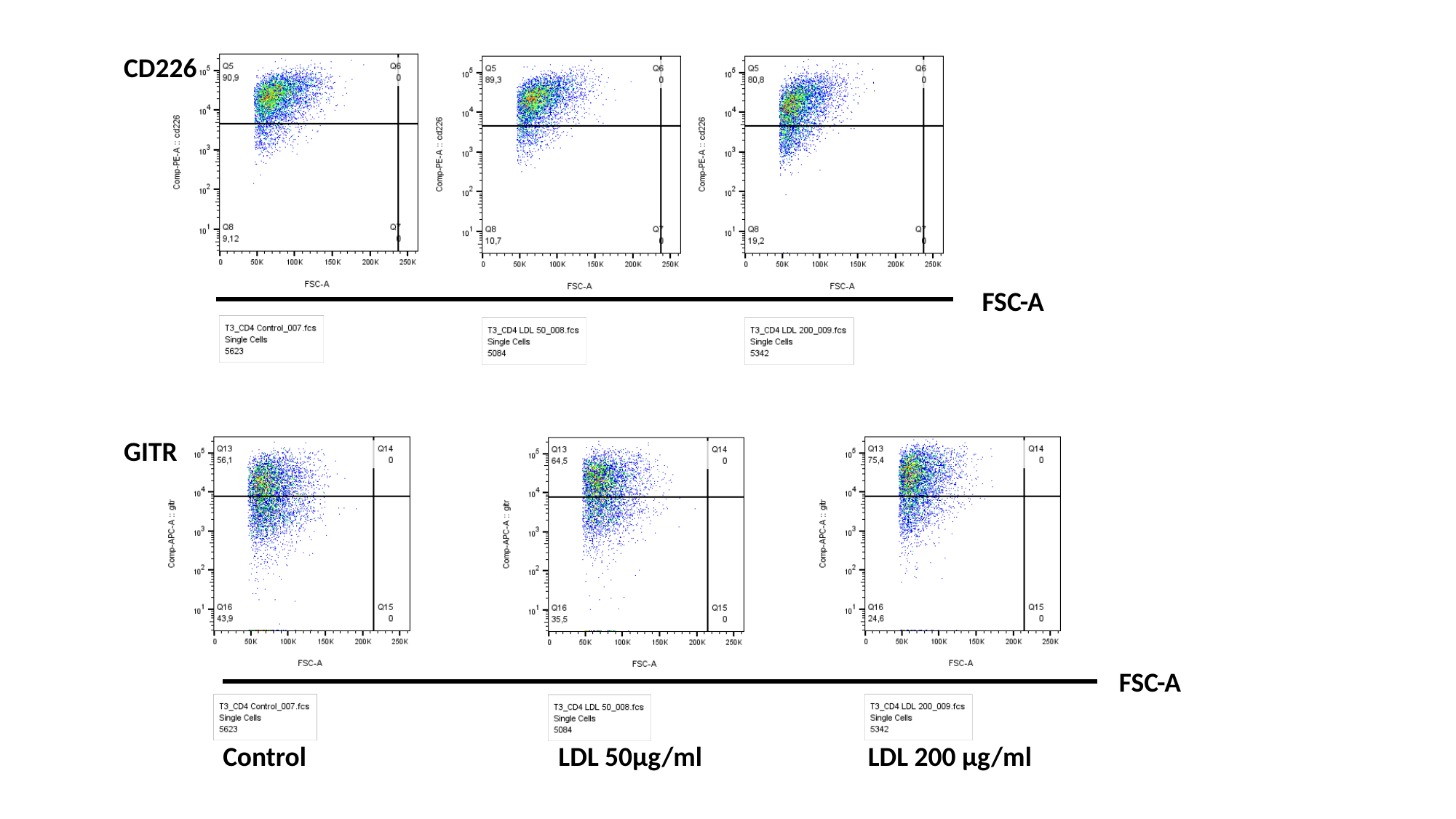

CD226
FSC-A
GITR
FSC-A
Control
LDL 50µg/ml
LDL 200 µg/ml

## Slide 9
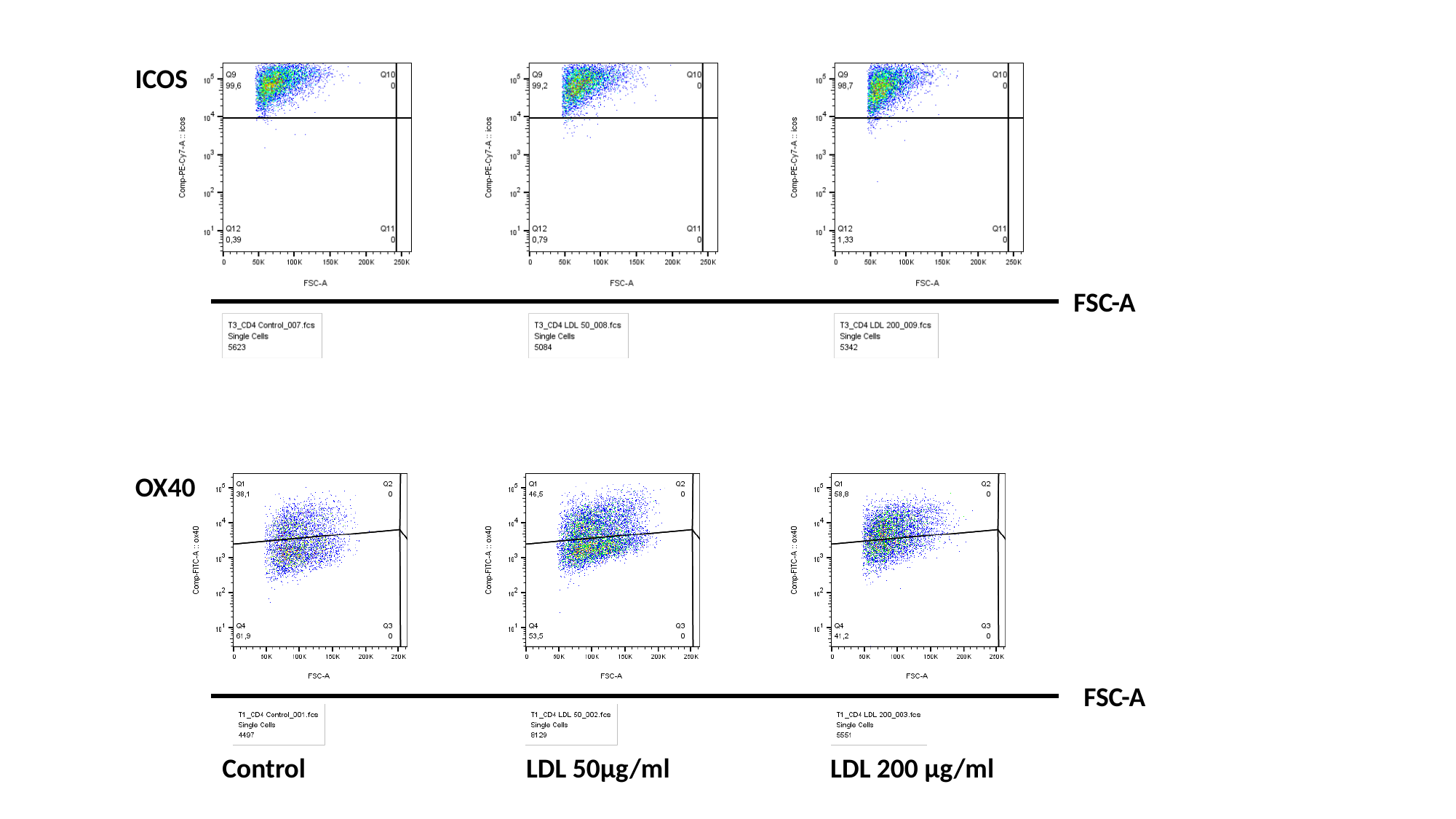

ICOS
FSC-A
OX40
FSC-A
Control
LDL 50µg/ml
LDL 200 µg/ml

## Slide 10
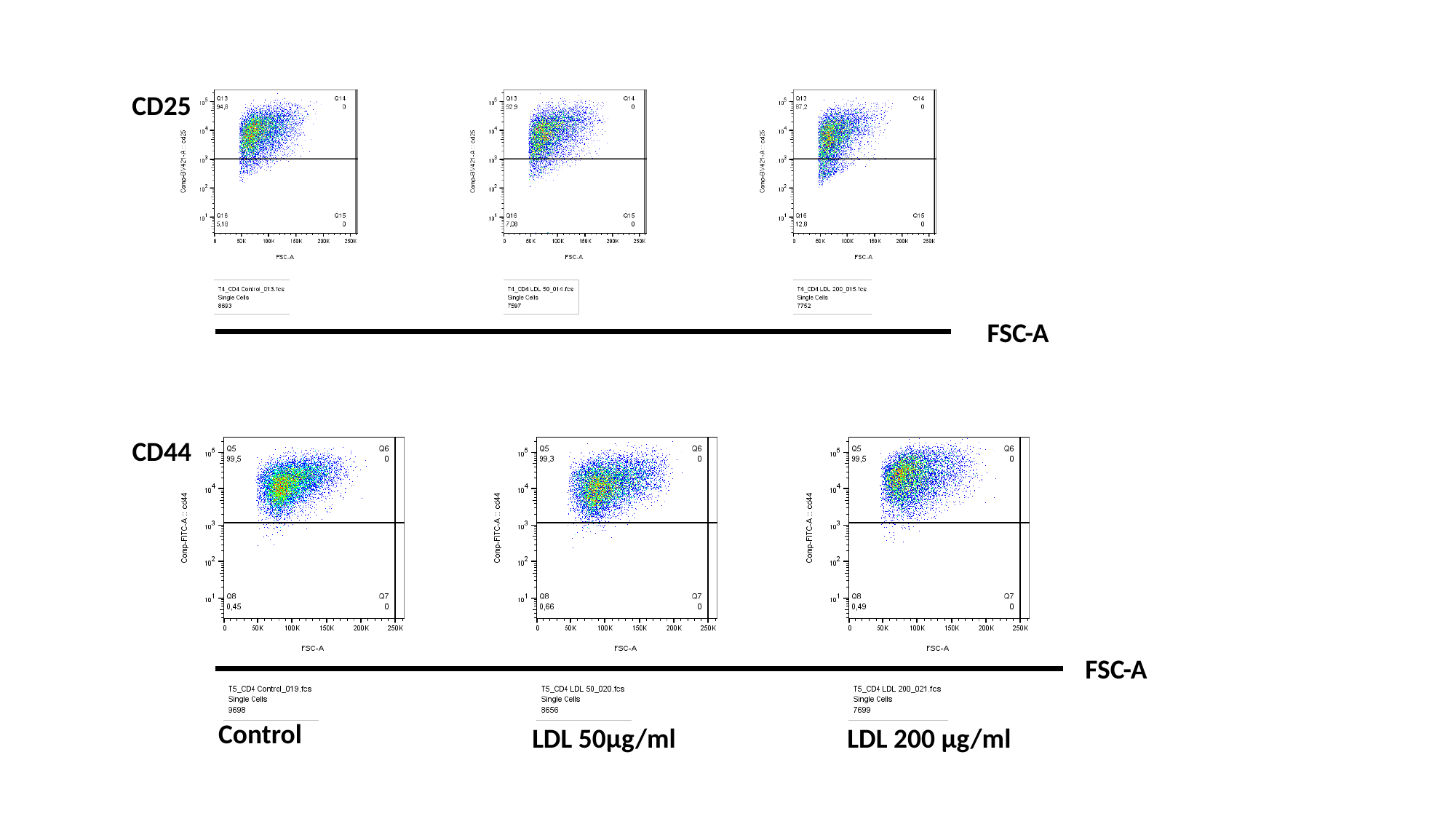

CD25
FSC-A
CD44
FSC-A
Control
LDL 50µg/ml
LDL 200 µg/ml

## Slide 11
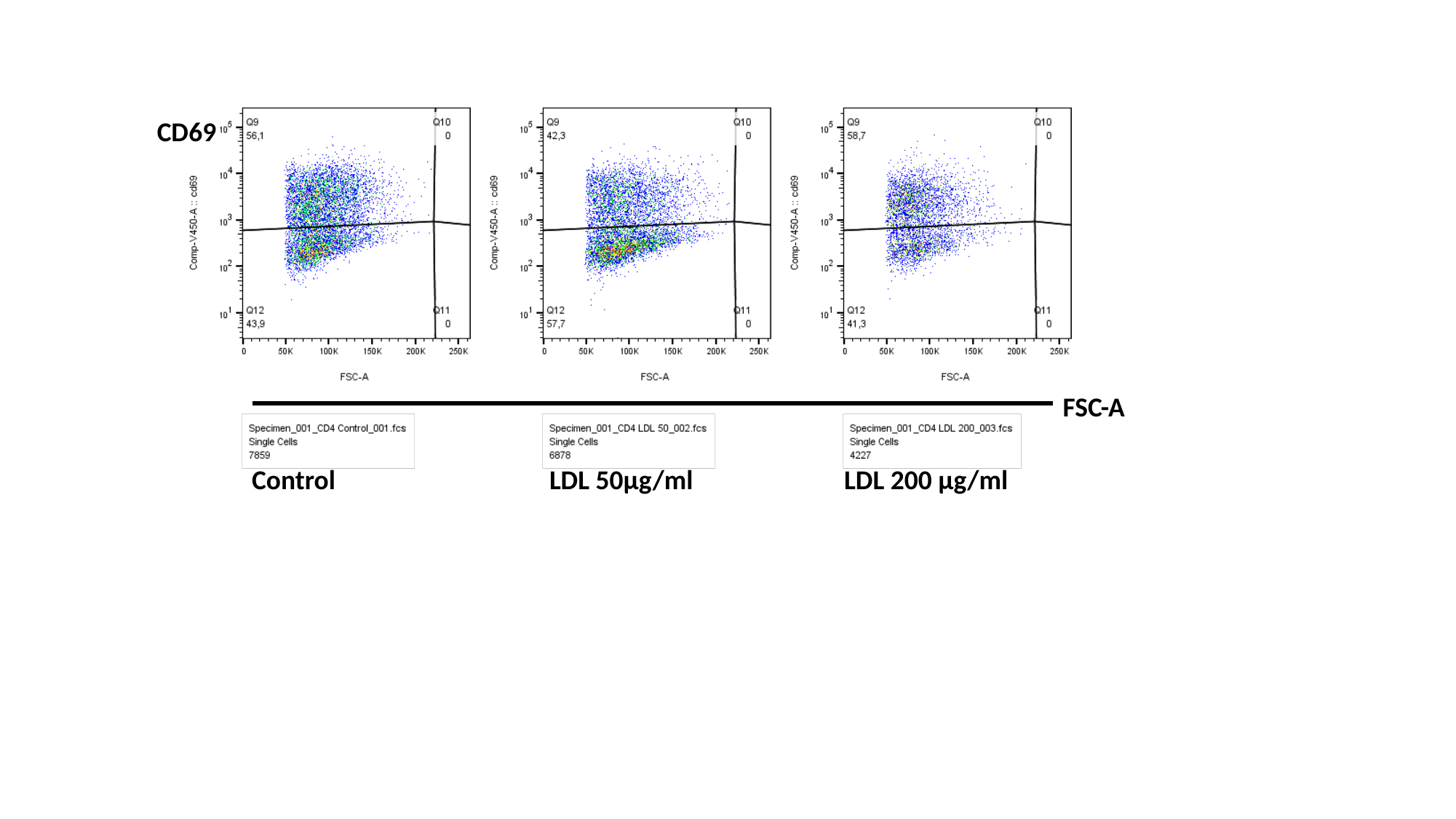

CD69
FSC-A
Control
LDL 50µg/ml
LDL 200 µg/ml

## Slide 12
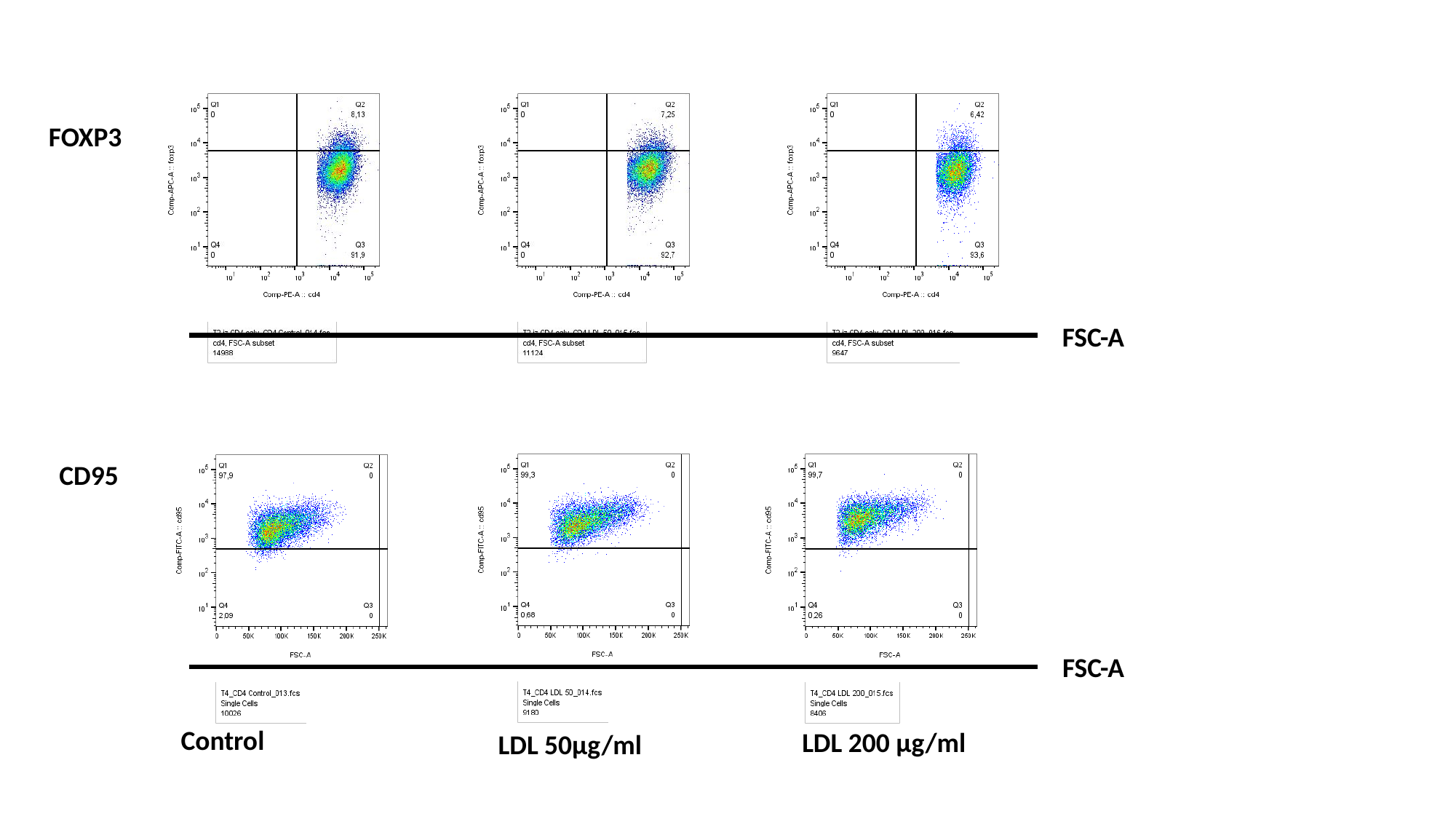

FOXP3
FSC-A
CD95
FSC-A
Control
LDL 200 µg/ml
LDL 50µg/ml

## Slide 13
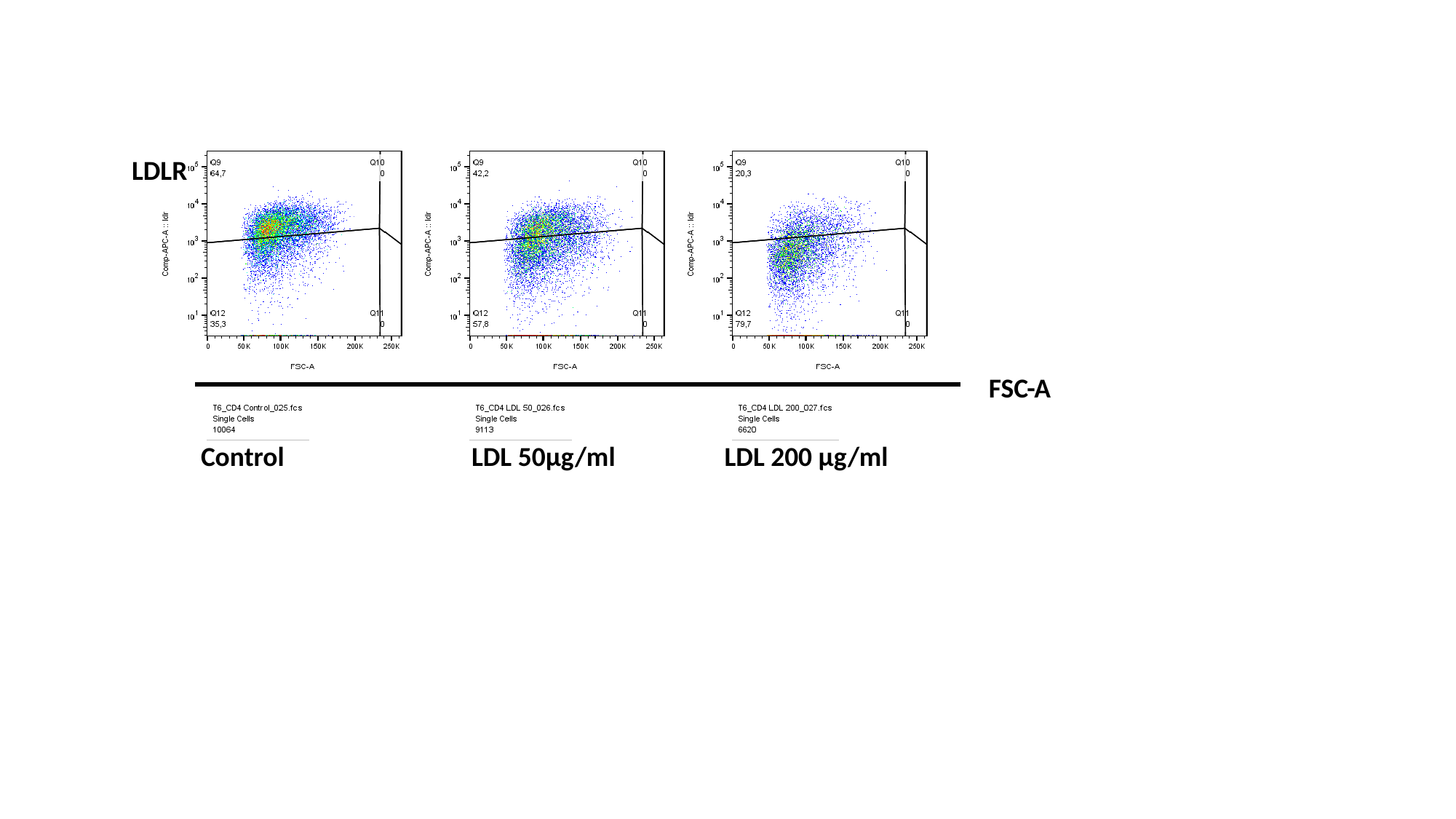

LDLR
FSC-A
Control
LDL 50µg/ml
LDL 200 µg/ml
