# Supplementary material for: Low-density lipoprotein balances T cell metabolism and enhances response to anti-PD-1 blockade in a HCT116 spheroid model
Source: Front Oncol. 2023 Jan 27;13:1107484. doi: 10.3389/fonc.2023.1107484 (PMC9911890; doi:10.3389/fonc.2023.1107484)
Supplement: Supplementary Data Sheet 1 — FACS gating MACS isolated CD4+ or CD8+ T cells [file DataSheet_1.zip › Supplement 1/Supplemental Data S10 Spheroid Statistics Green Object Total Area.PPTX]

## Slide 1
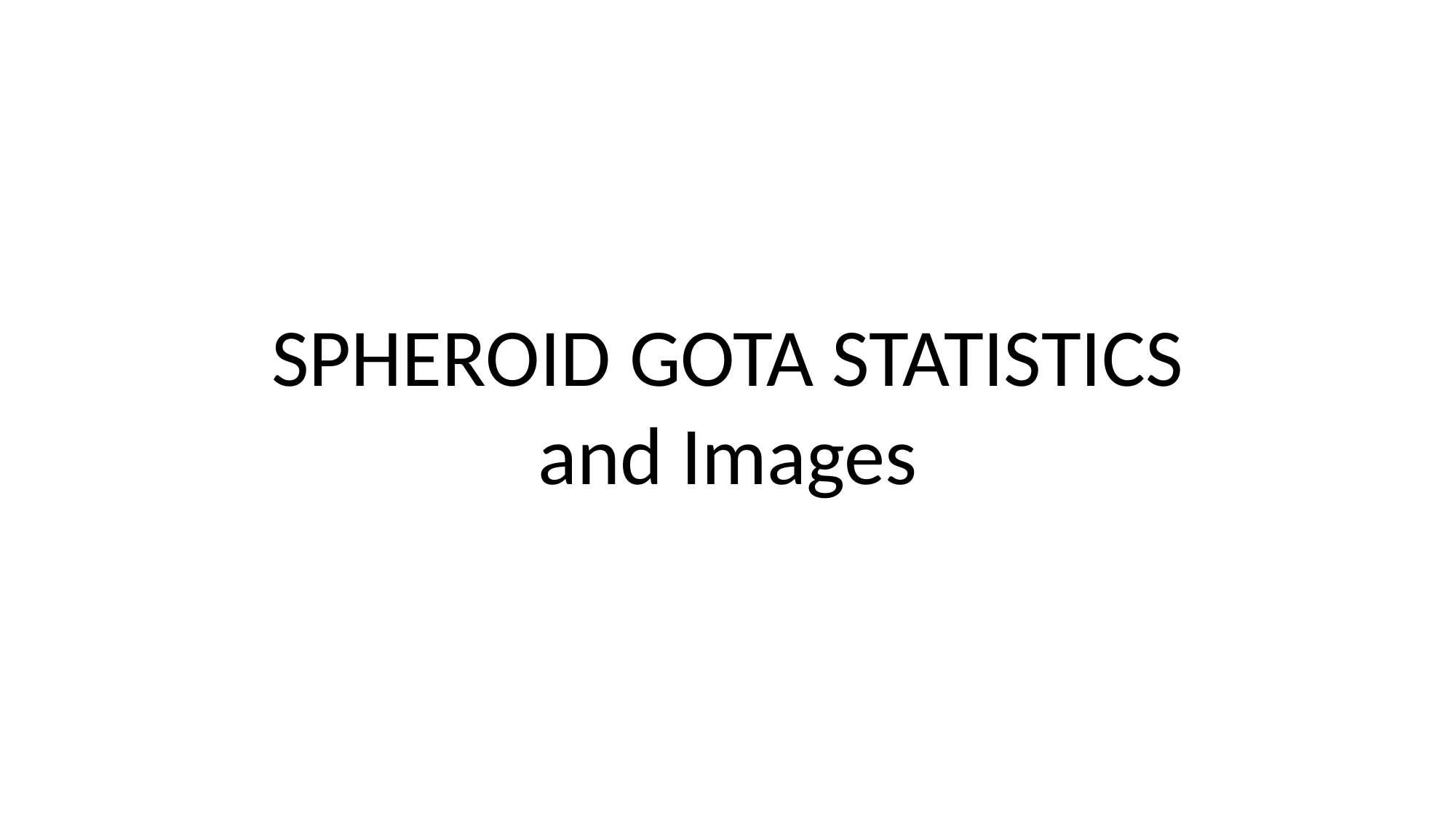

SPHEROID GOTA STATISTICS
and Images

## Slide 2
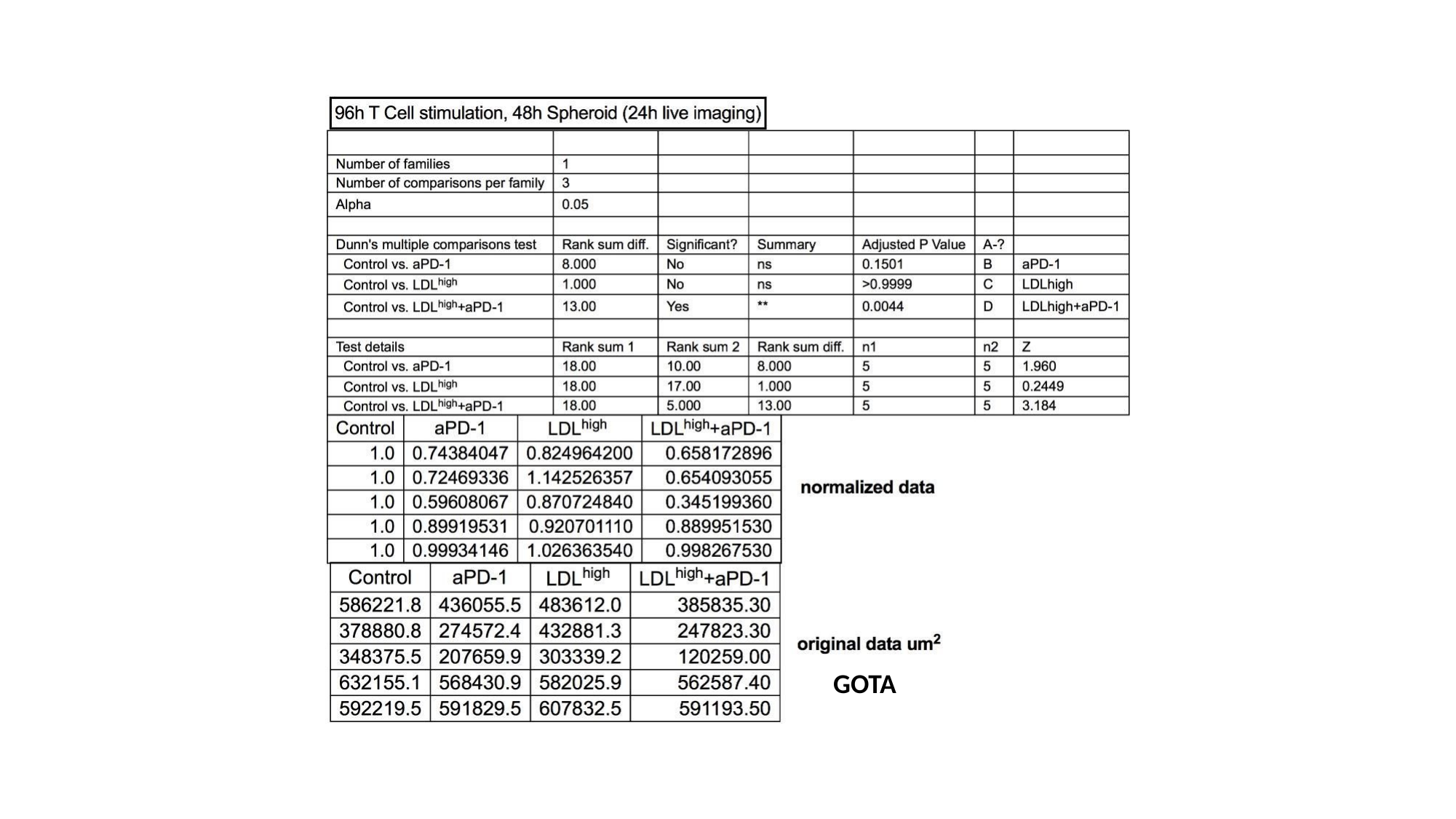

GOTA

## Slide 3
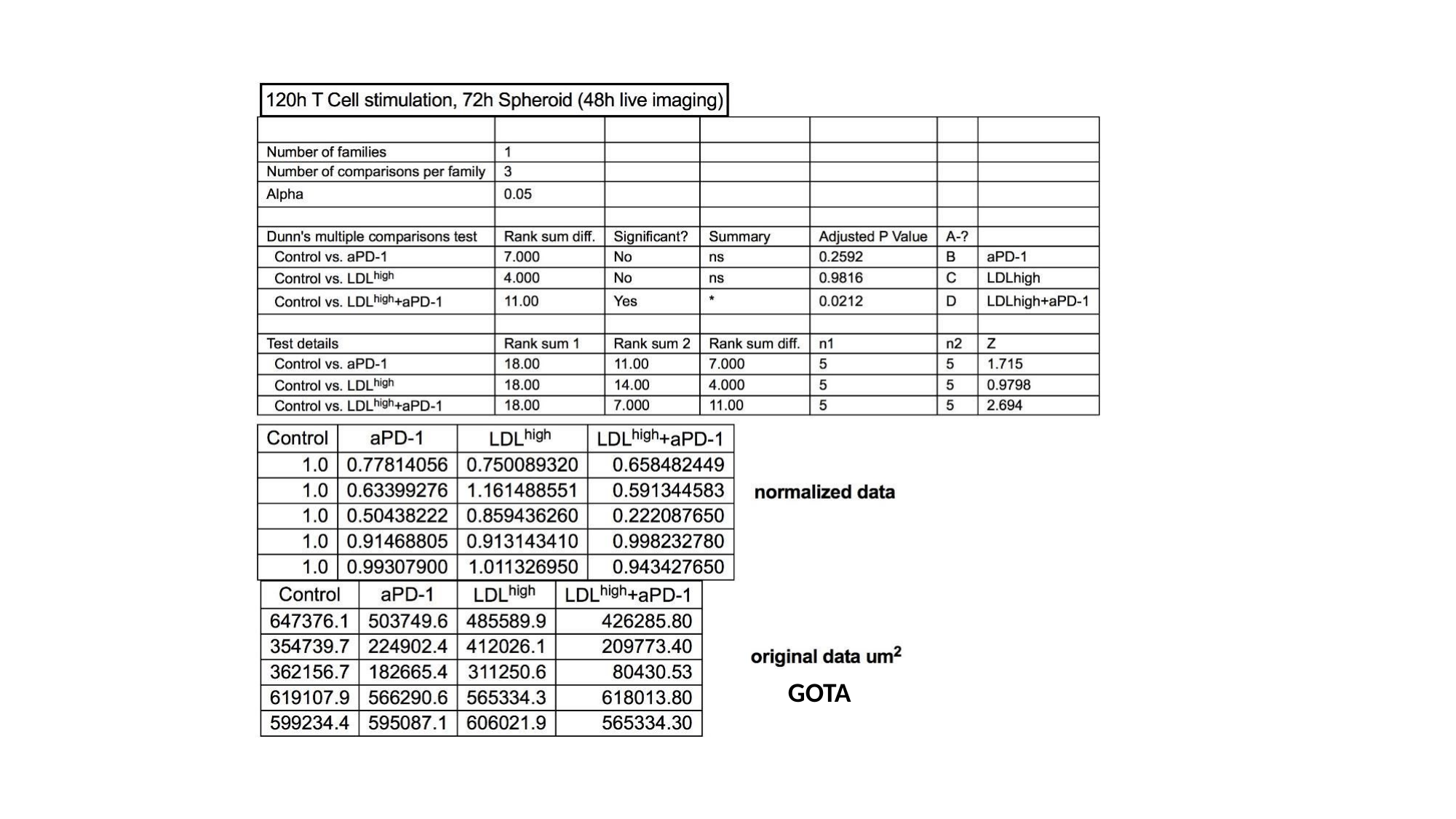

GOTA

## Slide 4
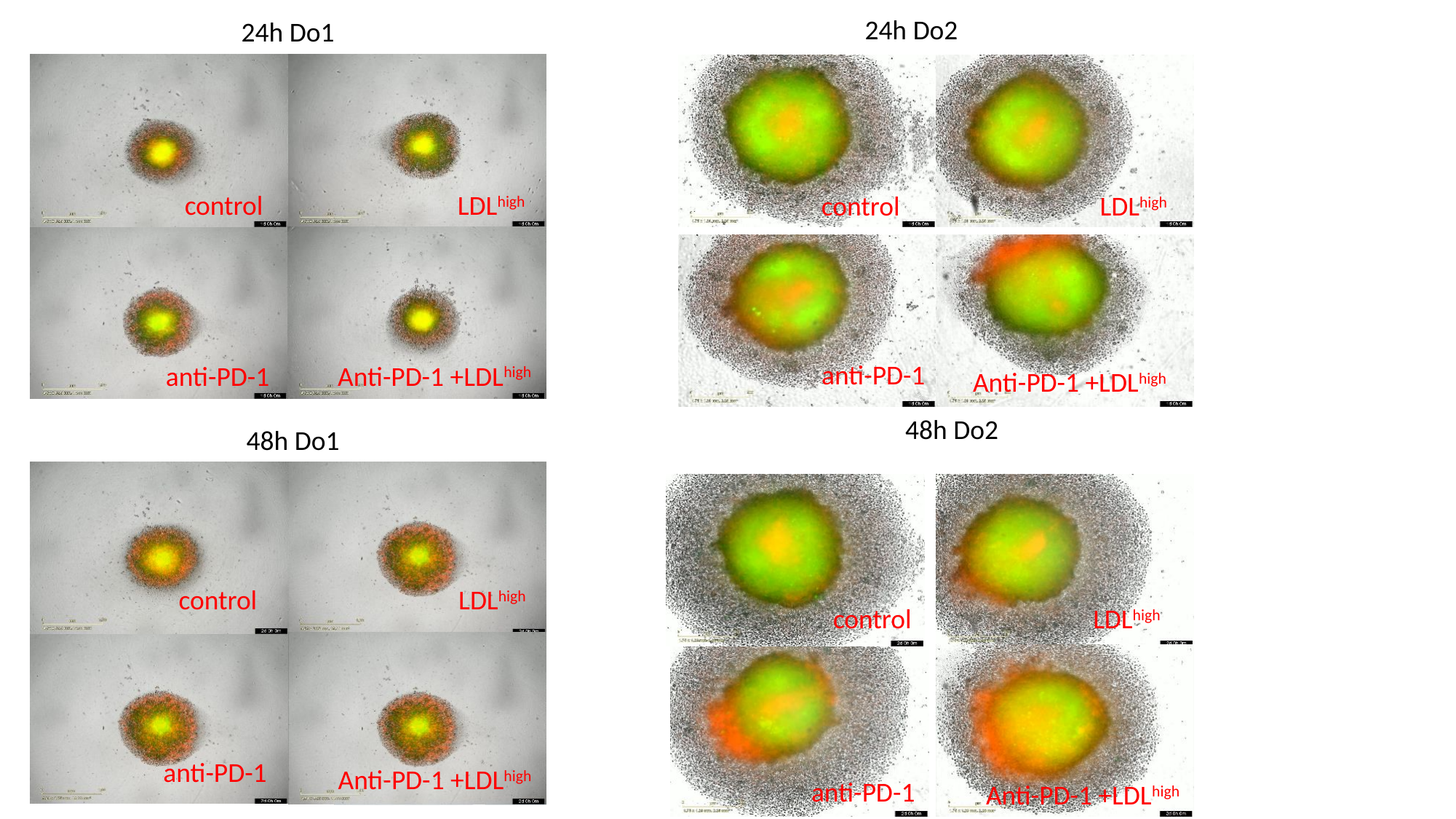

24h Do2
control
LDLhigh
anti-PD-1
Anti-PD-1 +LDLhigh
24h Do1
control
LDLhigh
anti-PD-1
Anti-PD-1 +LDLhigh
48h Do2
control
LDLhigh
anti-PD-1
Anti-PD-1 +LDLhigh
48h Do1
control
LDLhigh
anti-PD-1
Anti-PD-1 +LDLhigh

## Slide 5
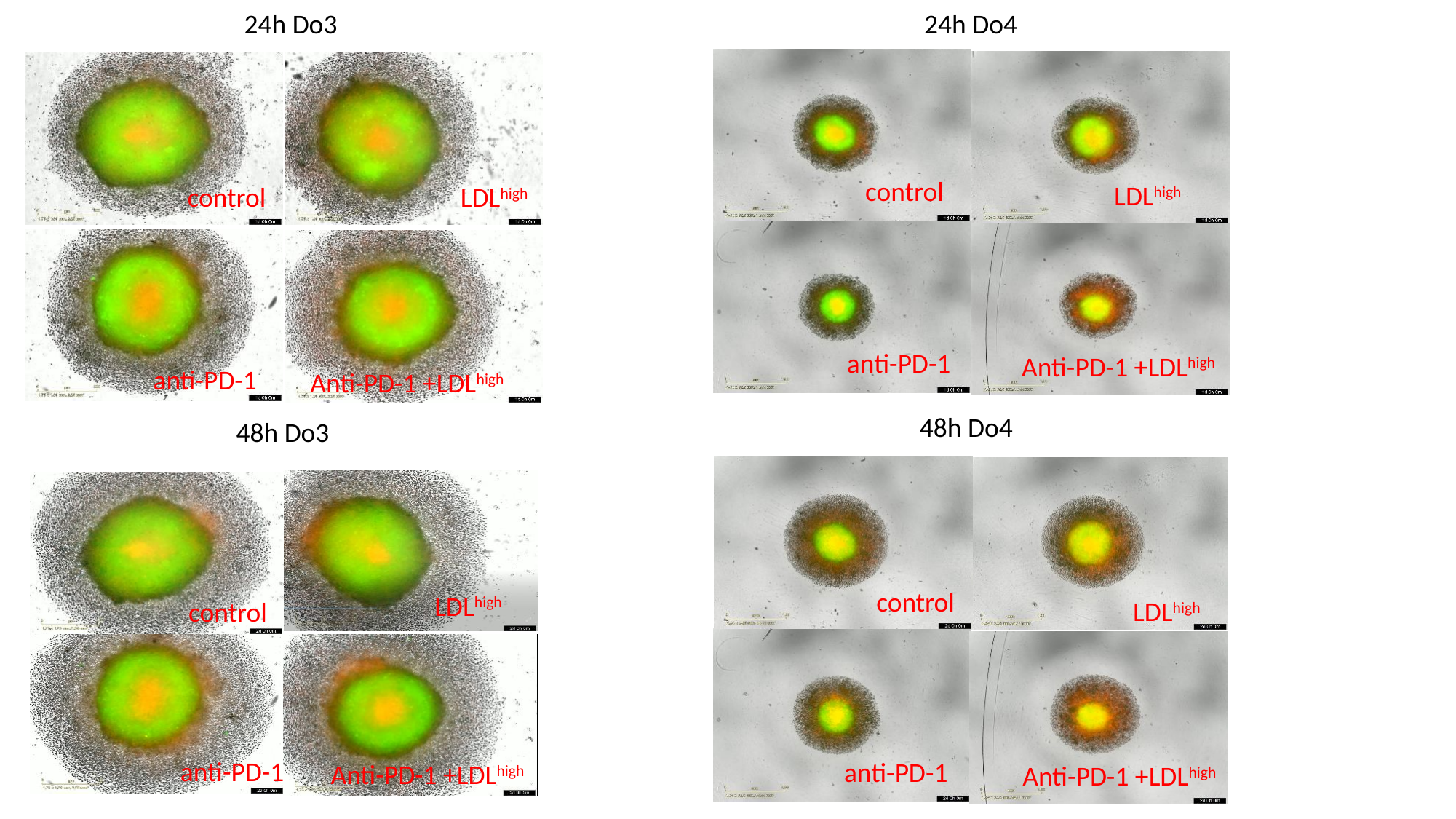

24h Do3
control
LDLhigh
anti-PD-1
Anti-PD-1 +LDLhigh
24h Do4
control
LDLhigh
anti-PD-1
Anti-PD-1 +LDLhigh
48h Do4
control
LDLhigh
anti-PD-1
Anti-PD-1 +LDLhigh
48h Do3
LDLhigh
control
anti-PD-1
Anti-PD-1 +LDLhigh

## Slide 6
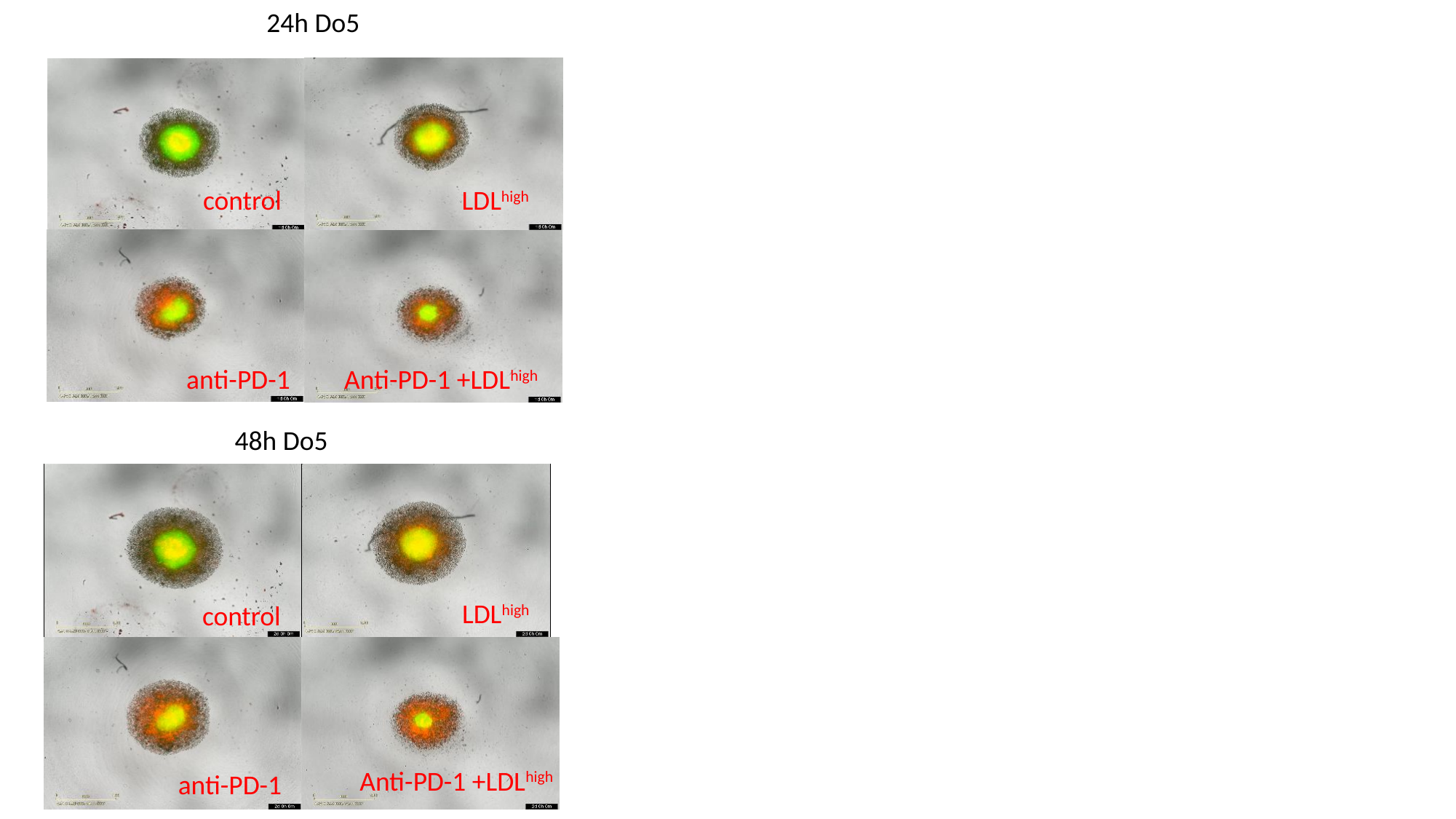

24h Do5
control
LDLhigh
anti-PD-1
Anti-PD-1 +LDLhigh
48h Do5
LDLhigh
control
Anti-PD-1 +LDLhigh
anti-PD-1
