# Supplementary material for: Low-density lipoprotein balances T cell metabolism and enhances response to anti-PD-1 blockade in a HCT116 spheroid model
Source: Front Oncol. 2023 Jan 27;13:1107484. doi: 10.3389/fonc.2023.1107484 (PMC9911890; doi:10.3389/fonc.2023.1107484)

## Slide 1
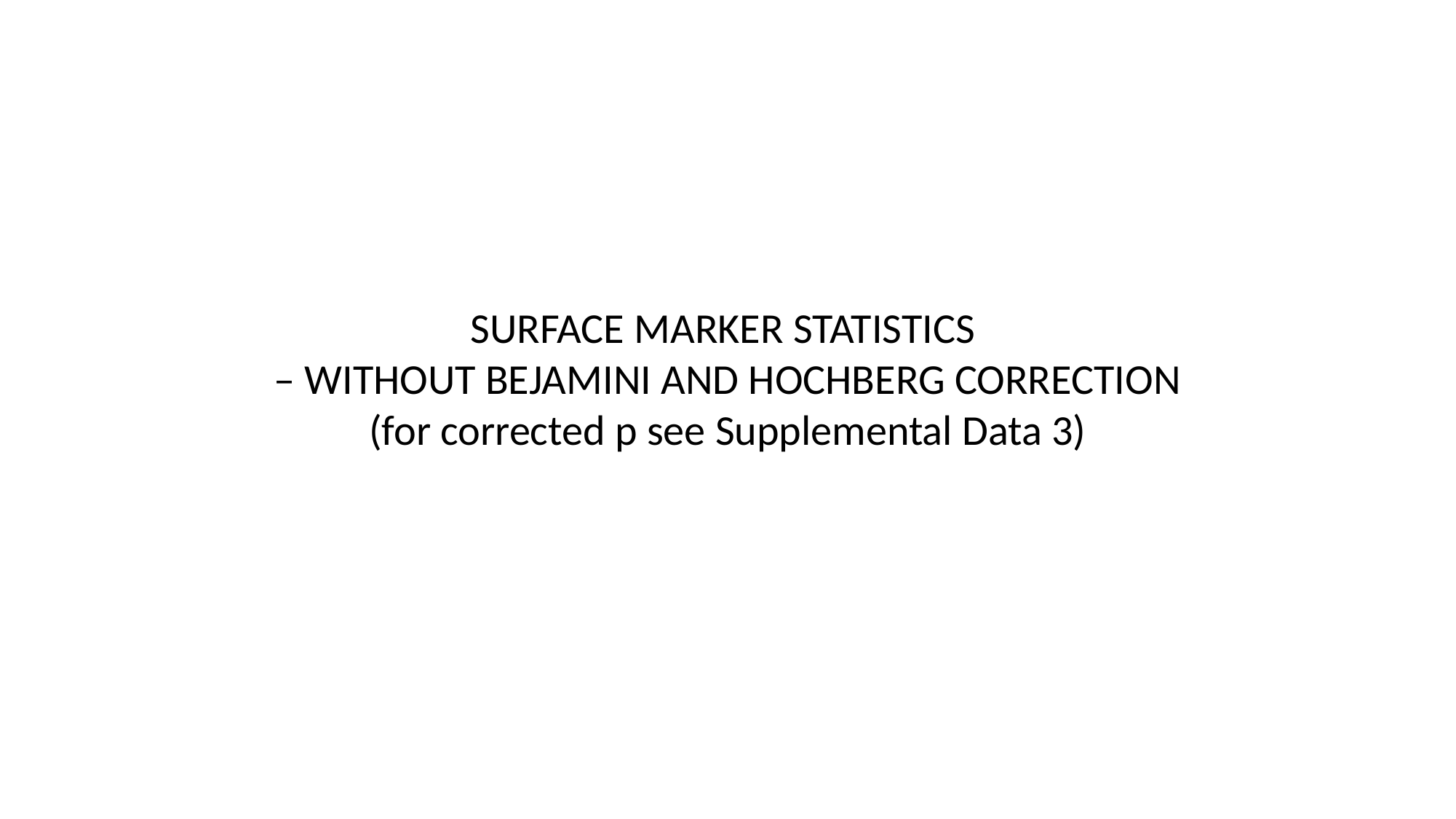

SURFACE MARKER STATISTICS
– WITHOUT BEJAMINI AND HOCHBERG CORRECTION
(for corrected p see Supplemental Data 3)

## Slide 2
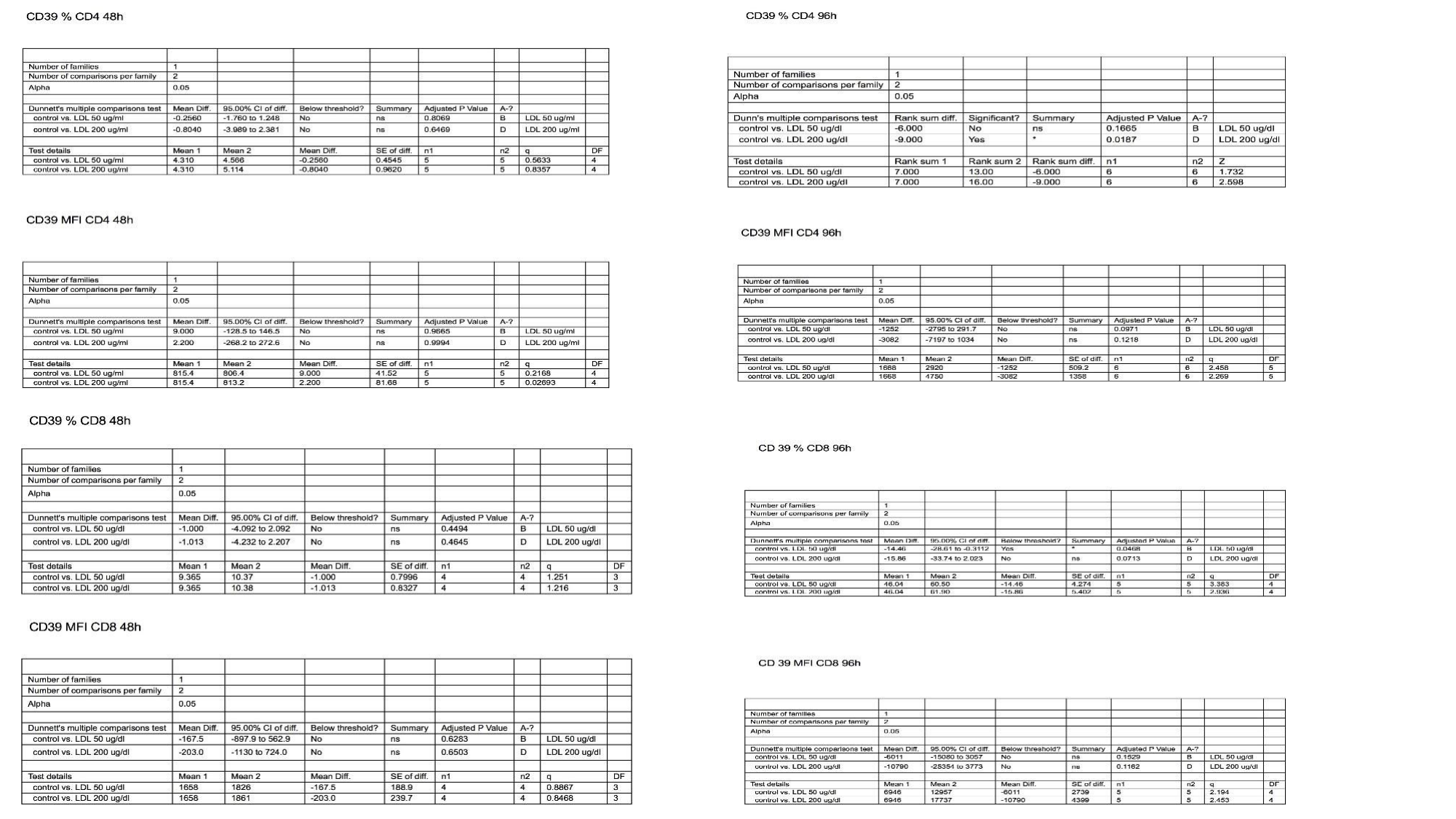

## Slide 3
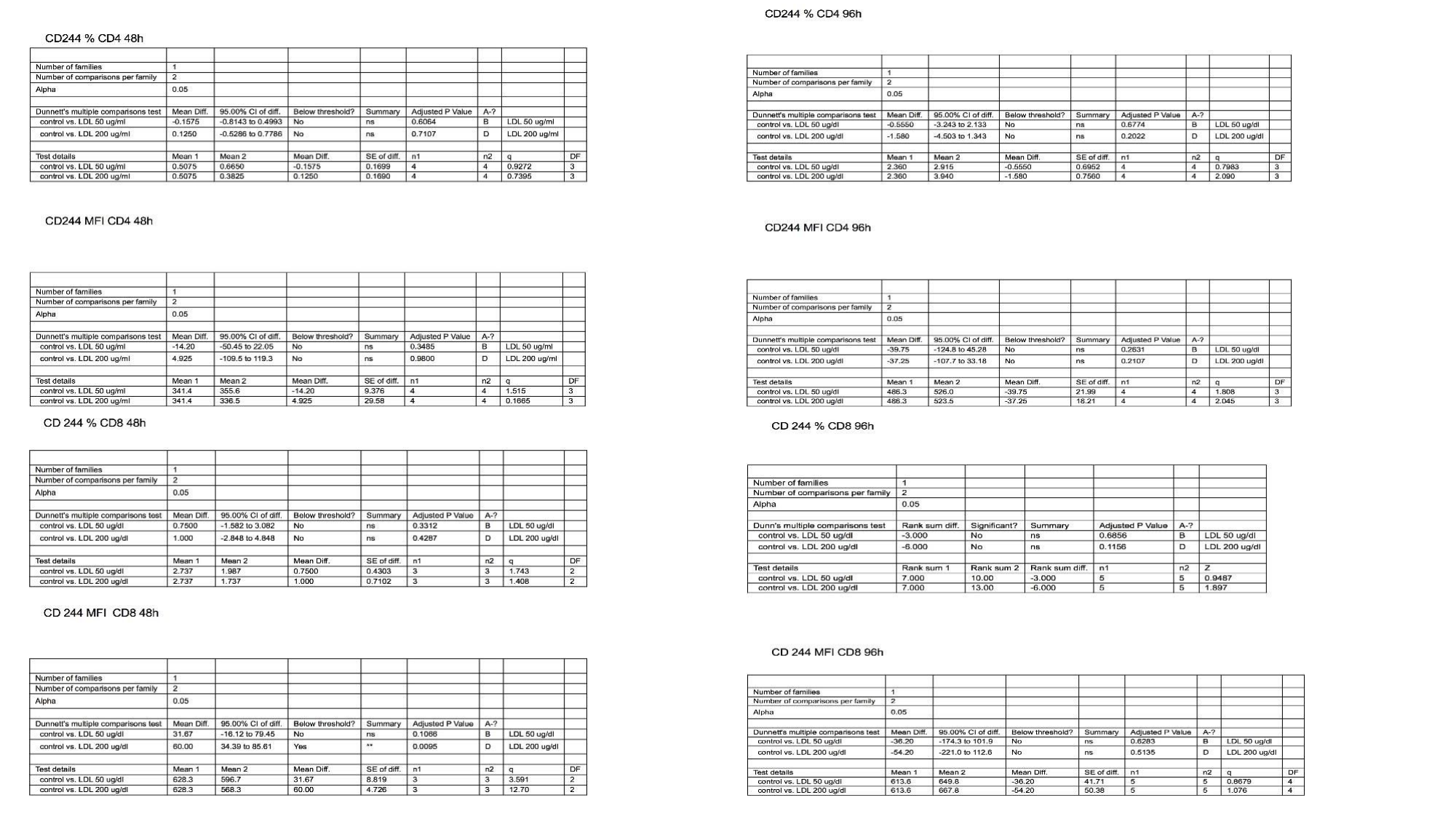

## Slide 4
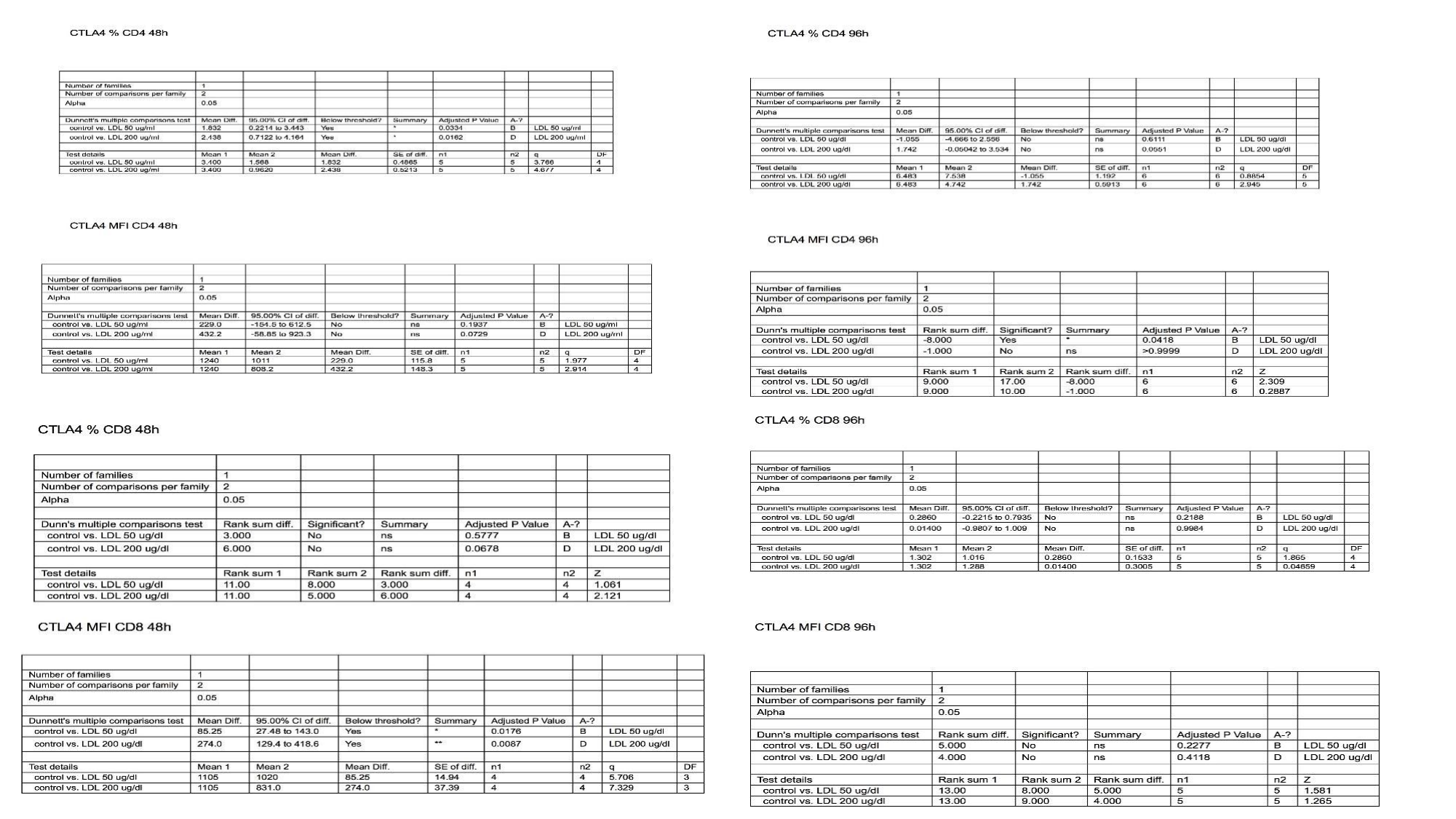

## Slide 5
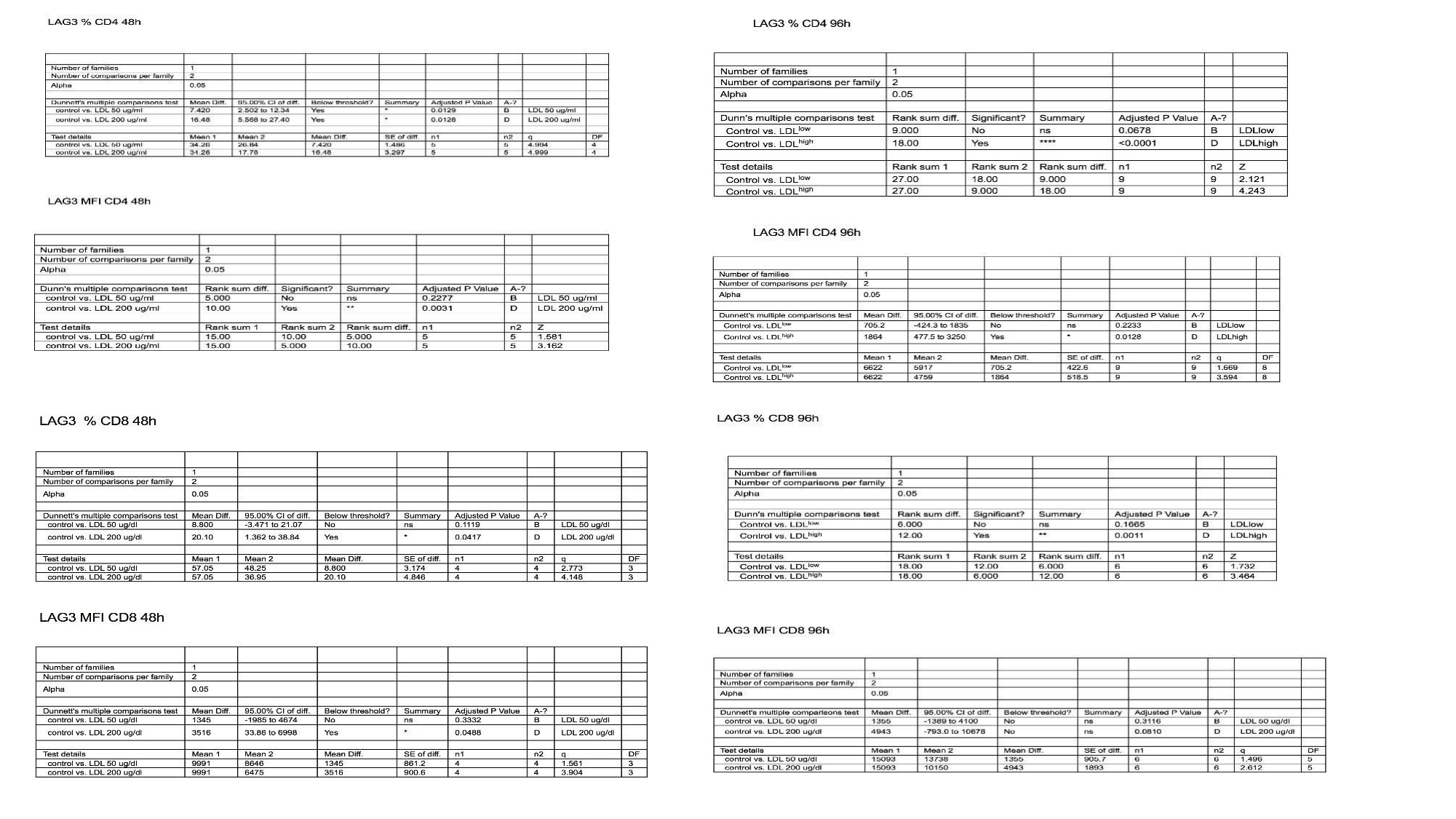

## Slide 6
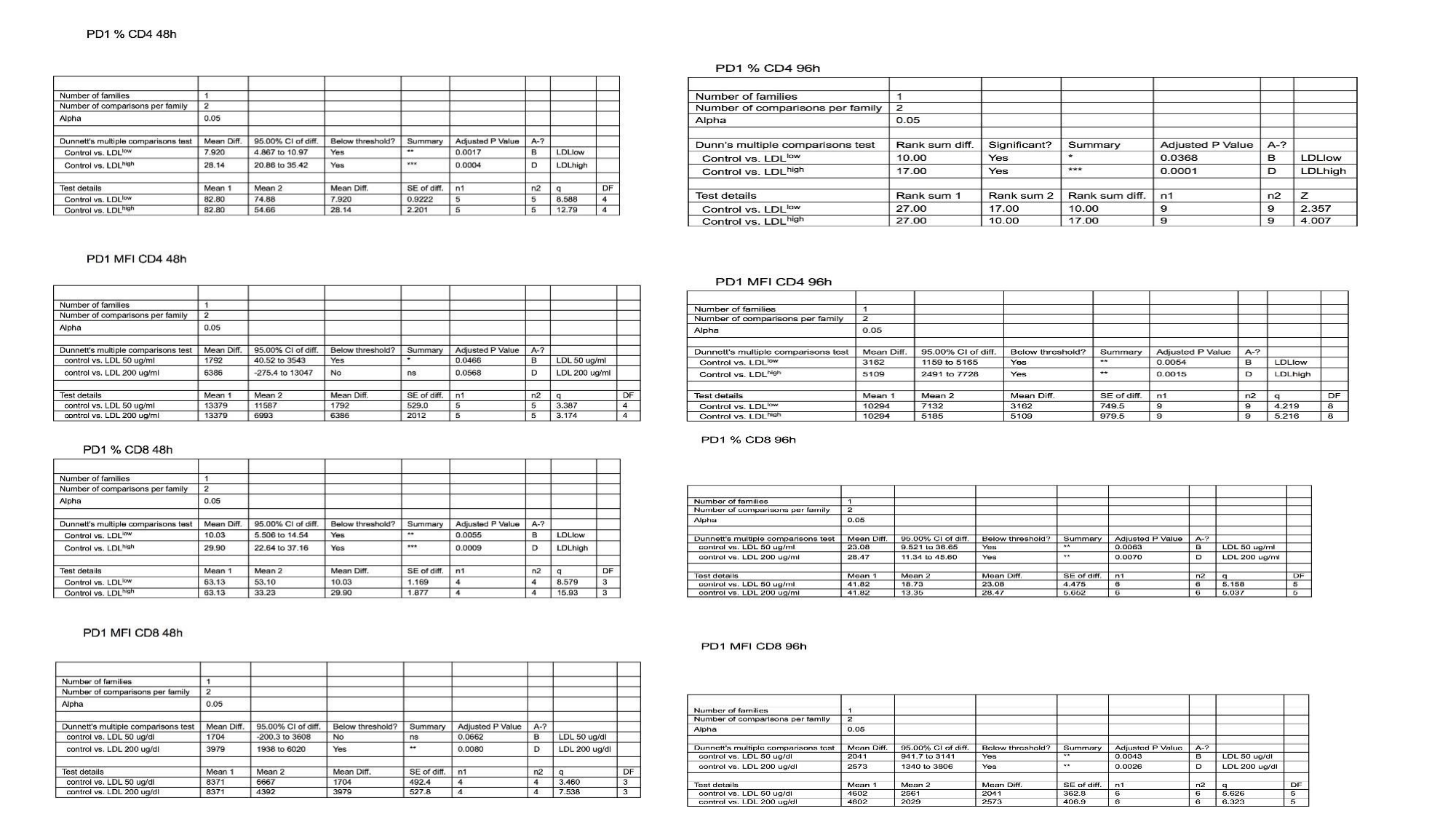

## Slide 7
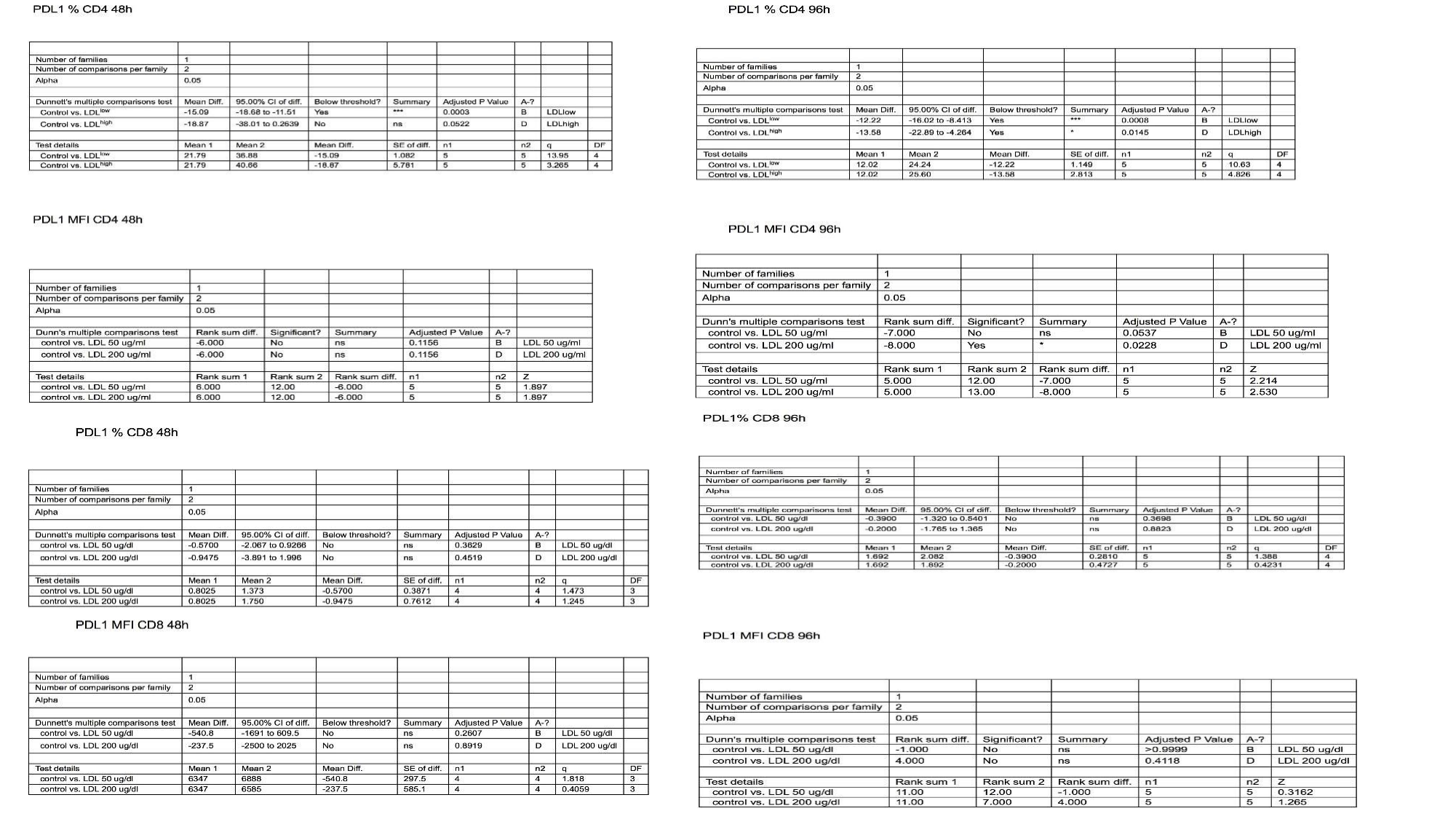

## Slide 8
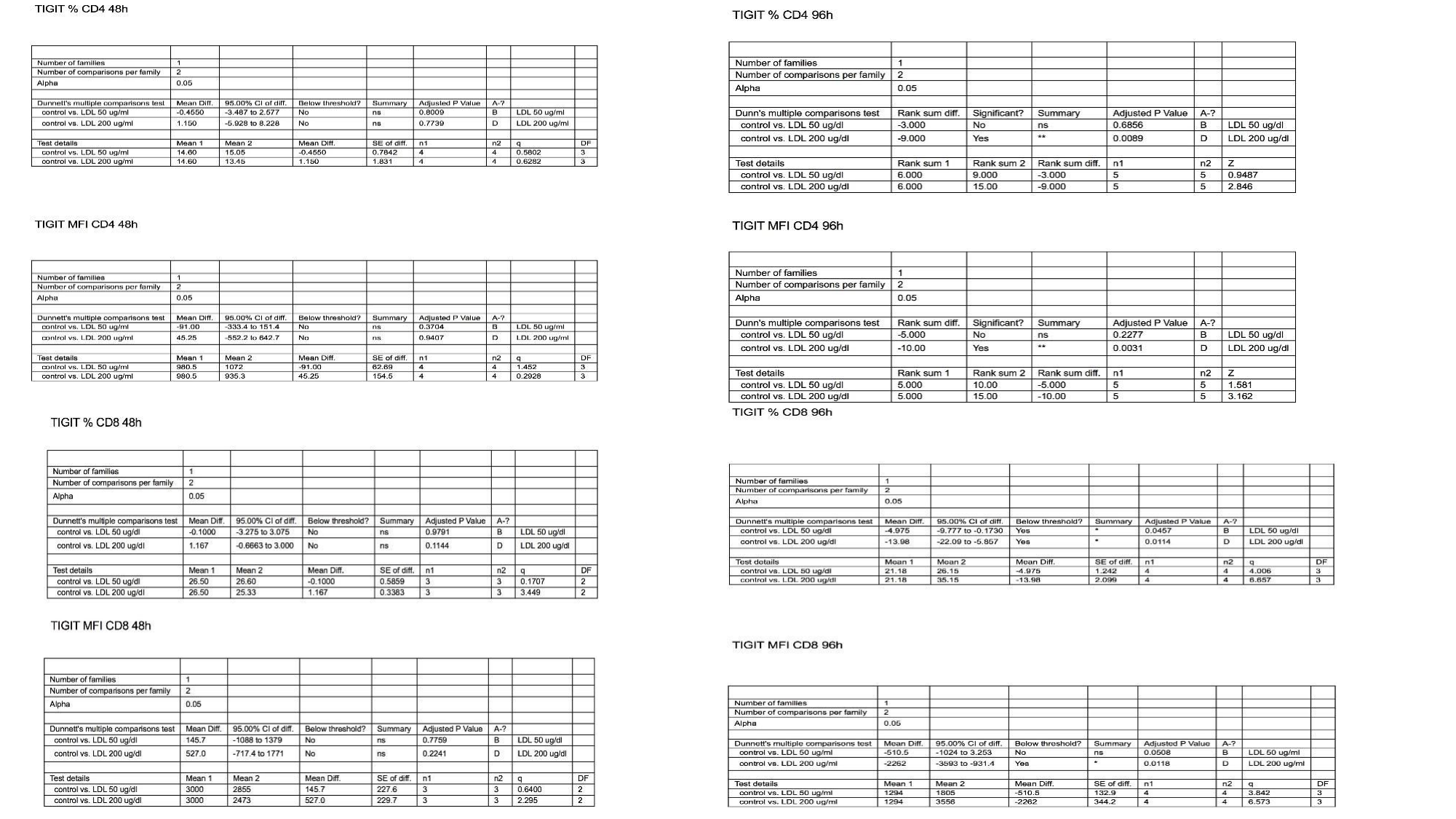

## Slide 9
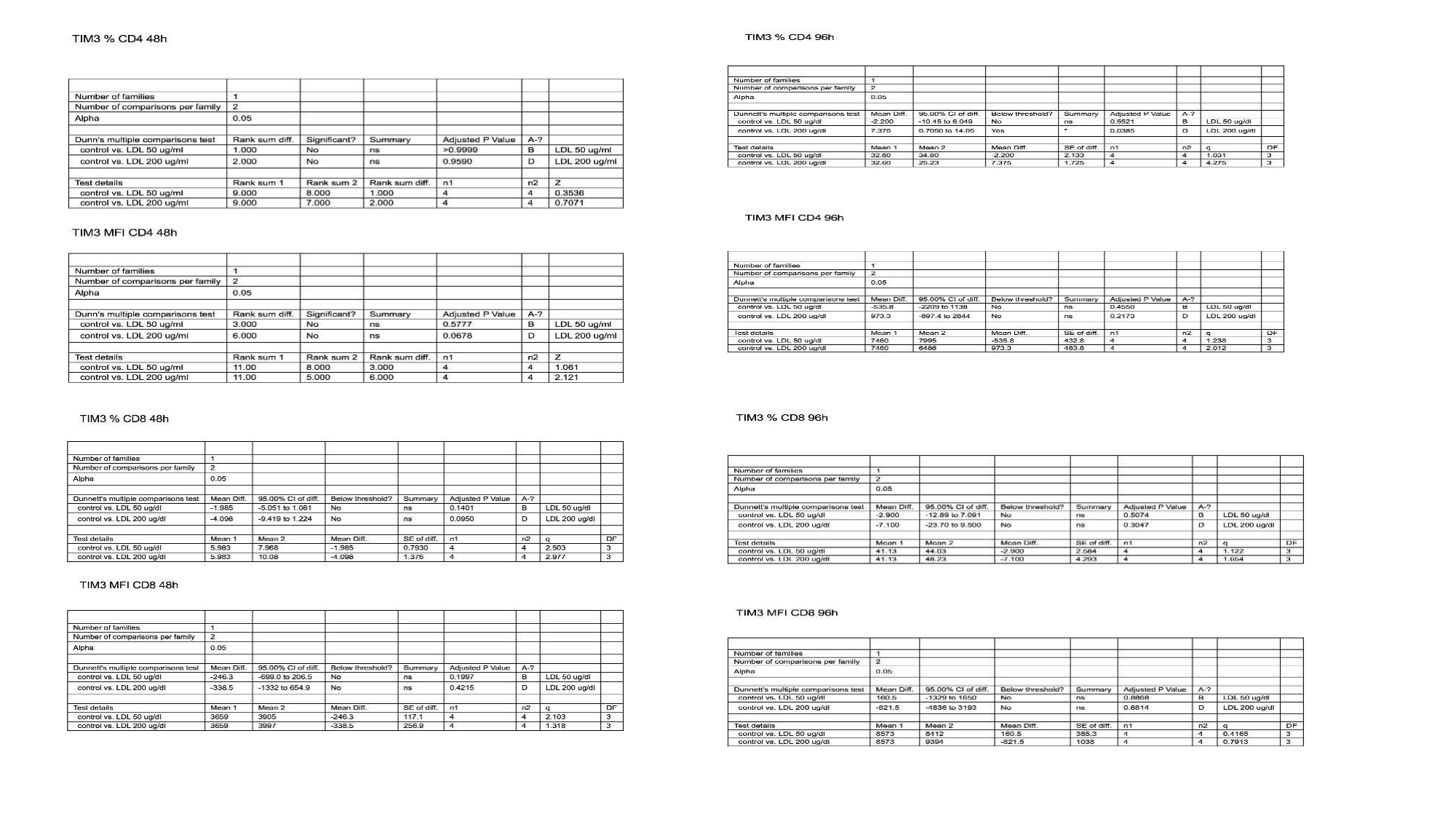

## Slide 10
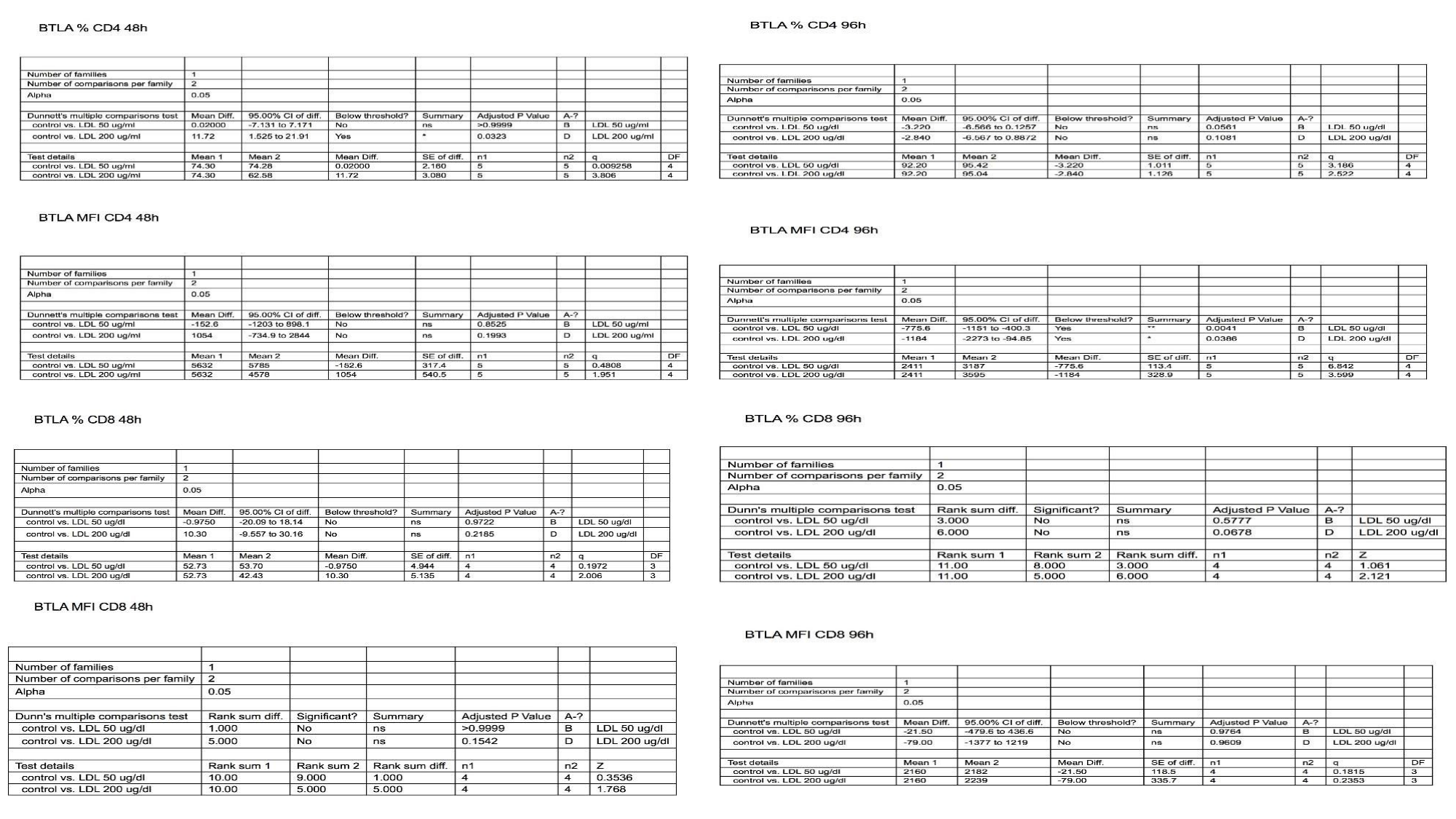

## Slide 11
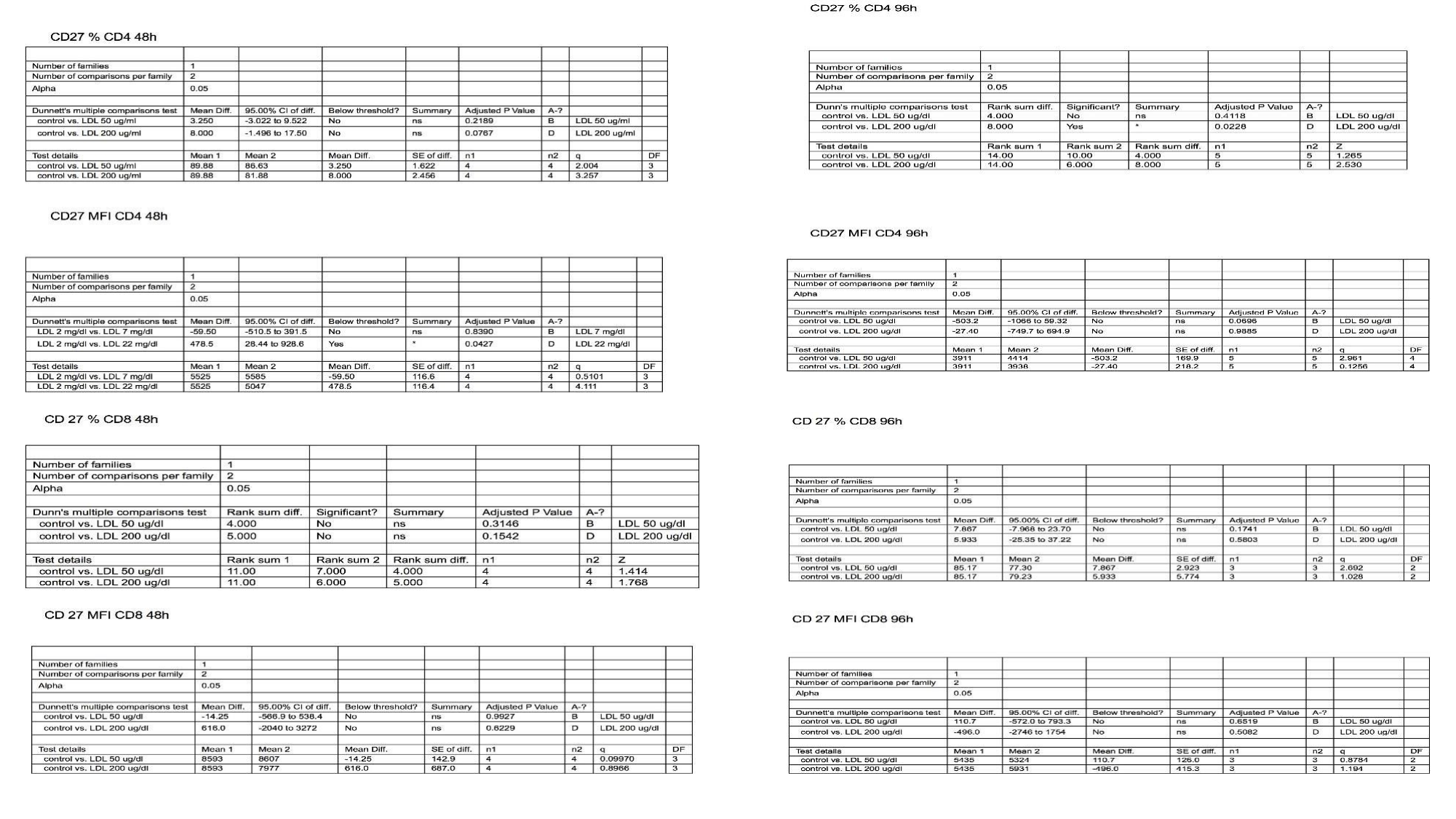

## Slide 12
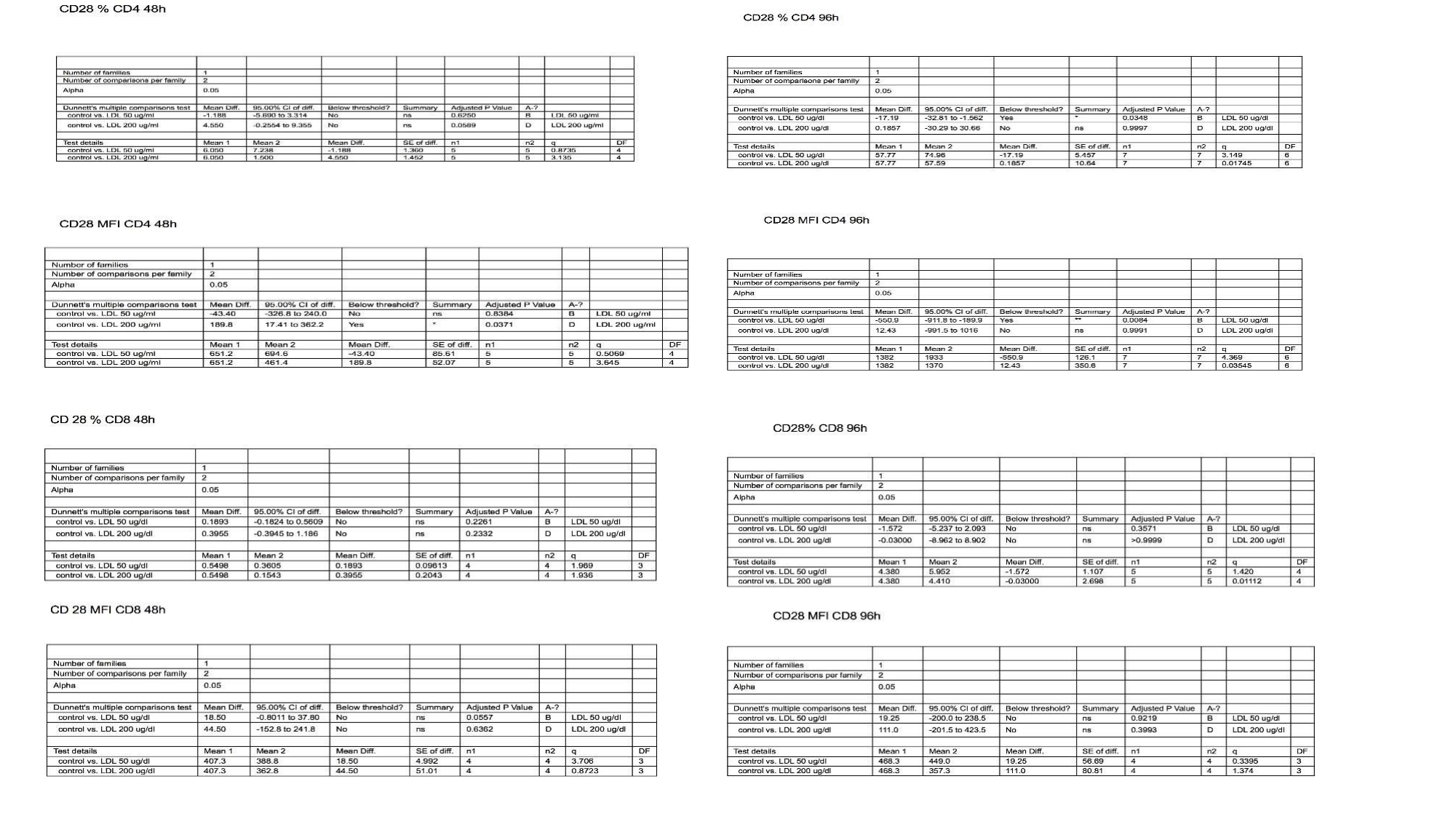

## Slide 13
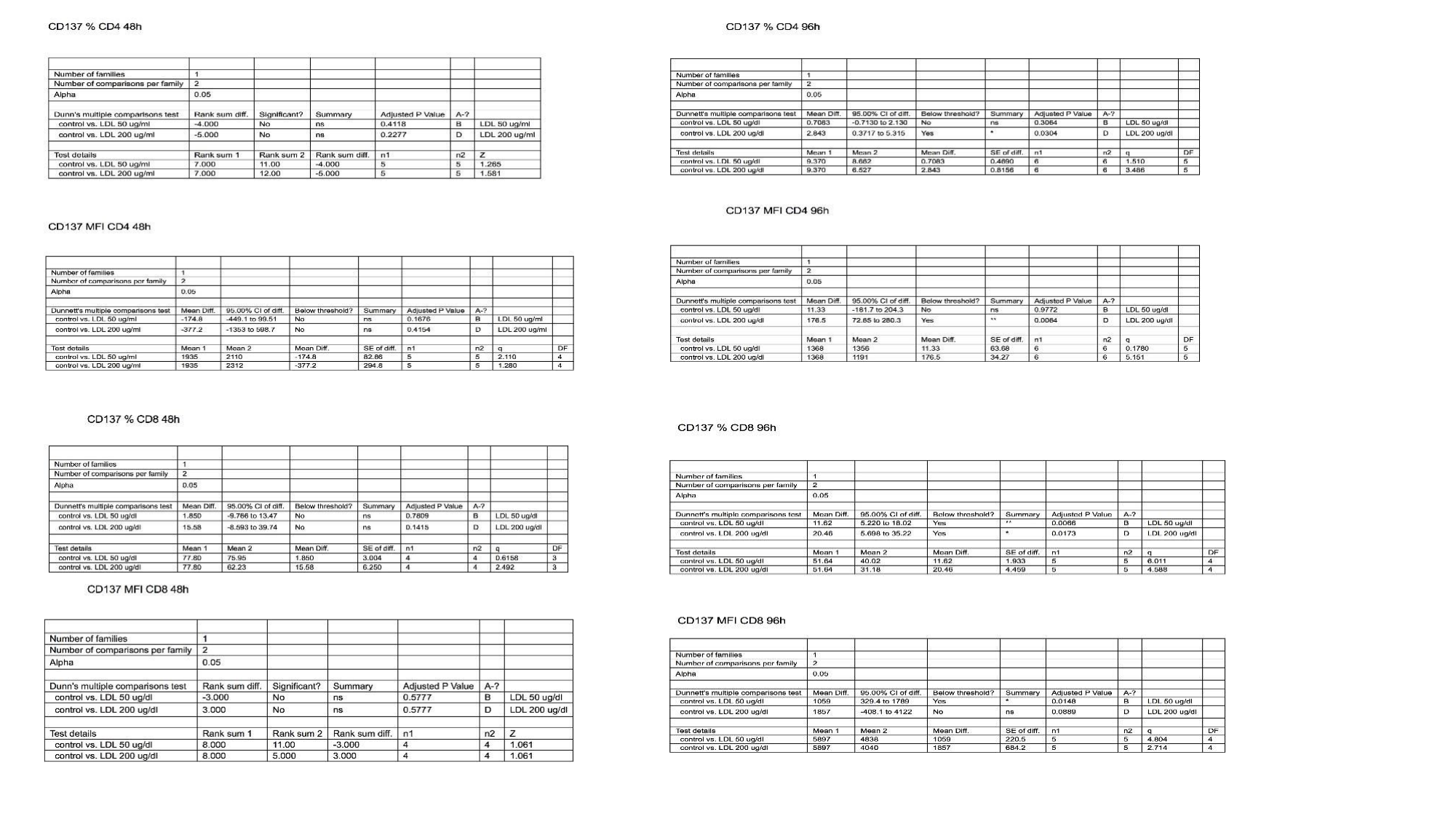

## Slide 14
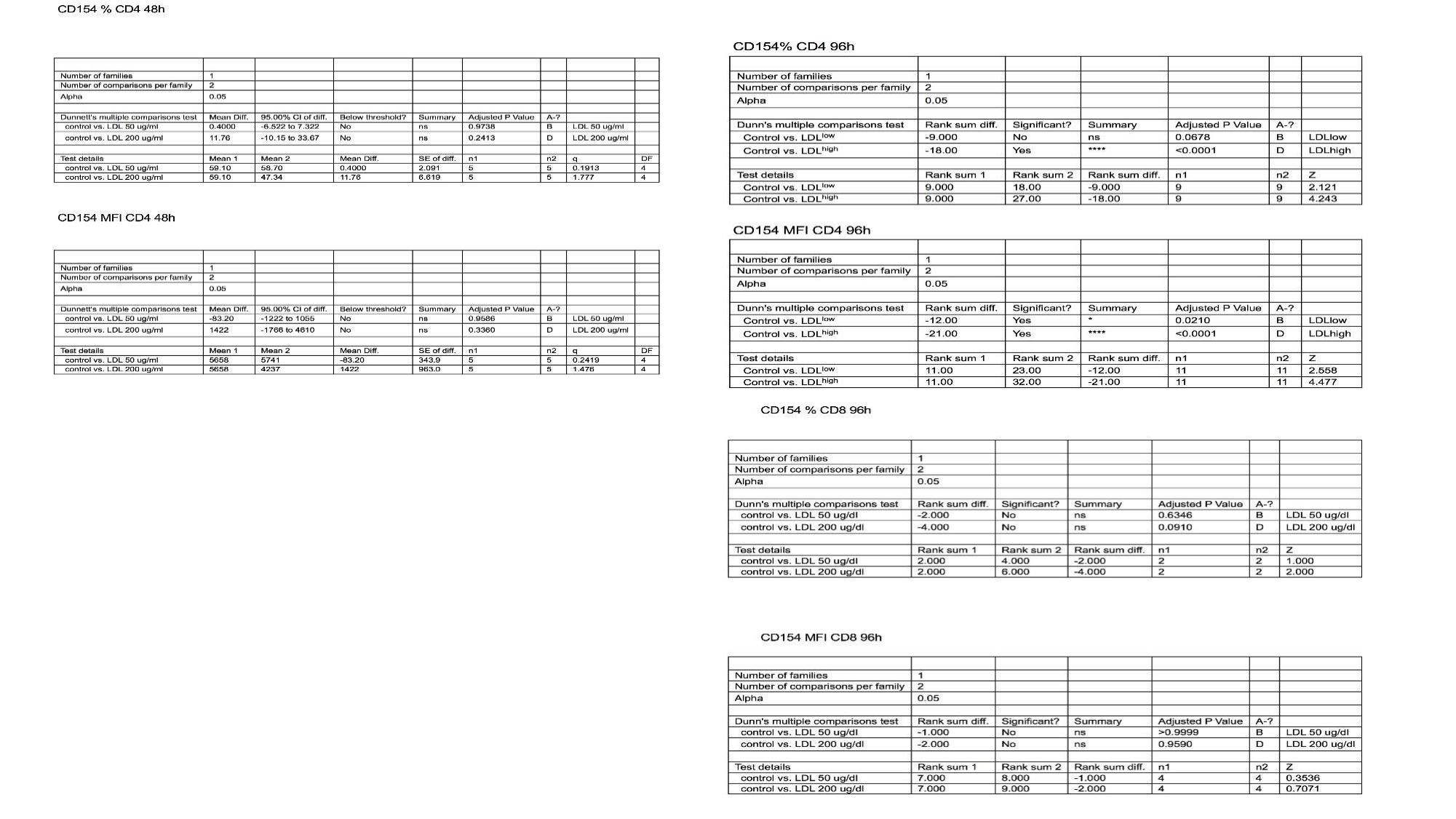

## Slide 15
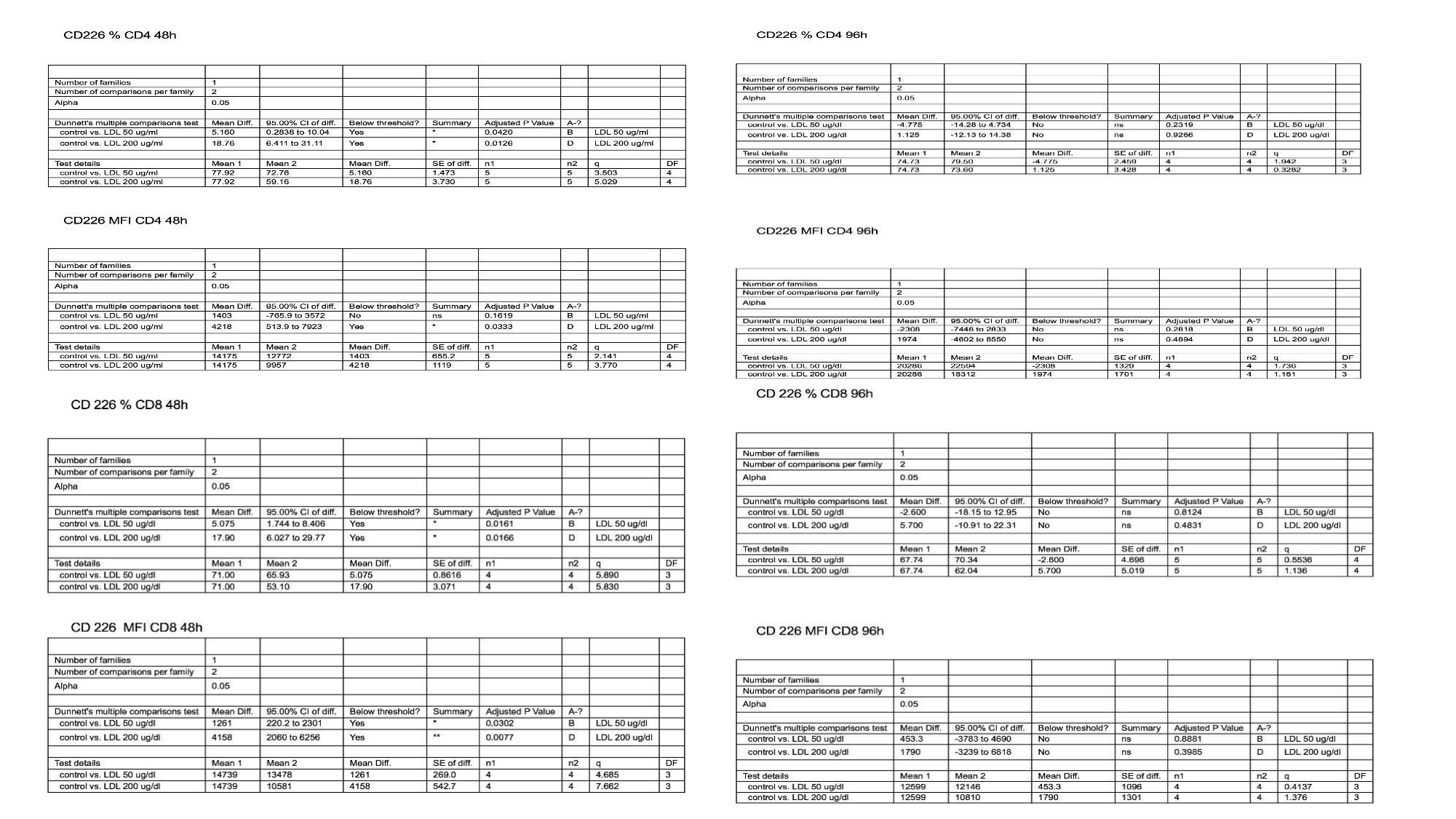

## Slide 16
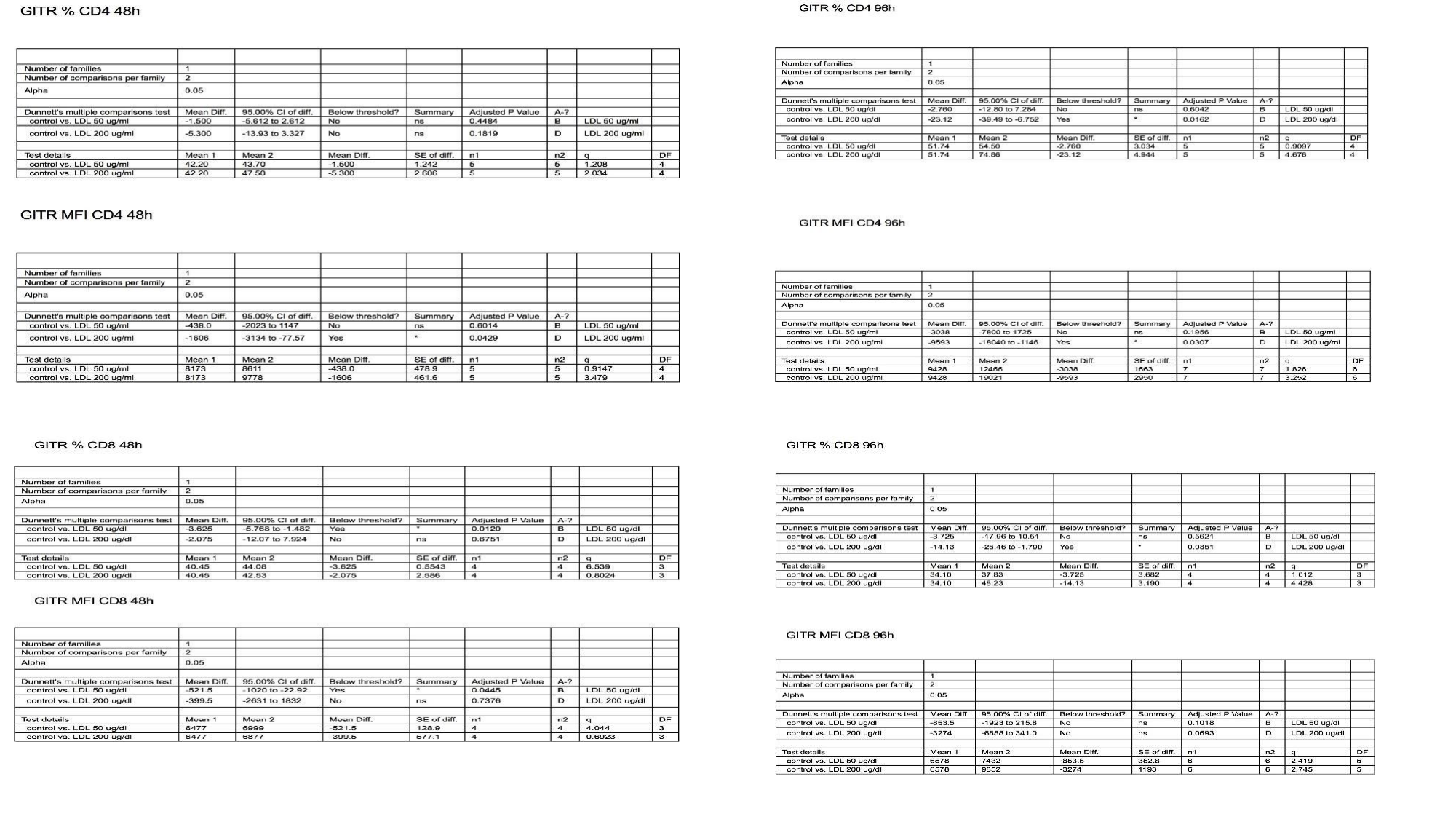

## Slide 17
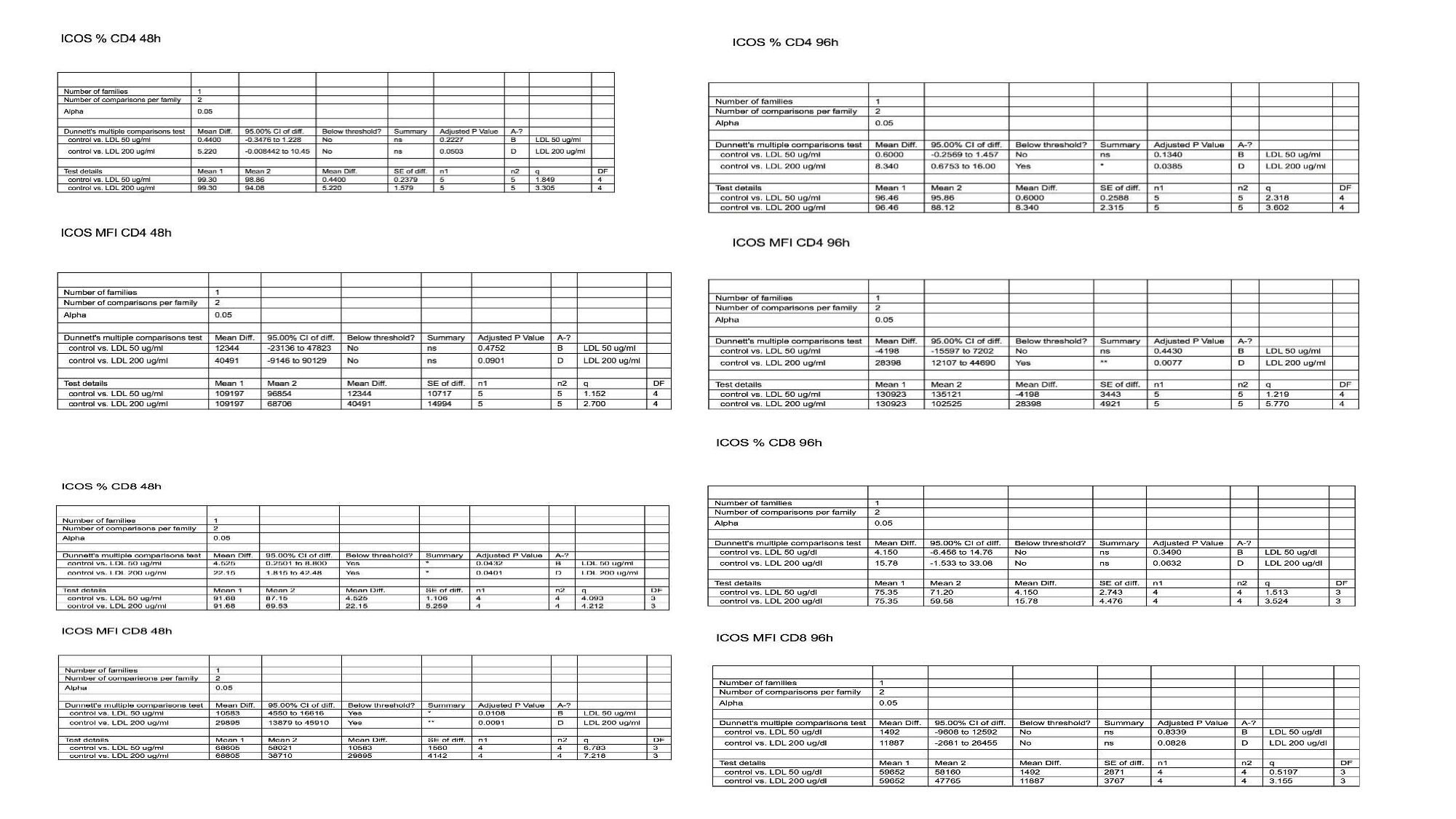

## Slide 18
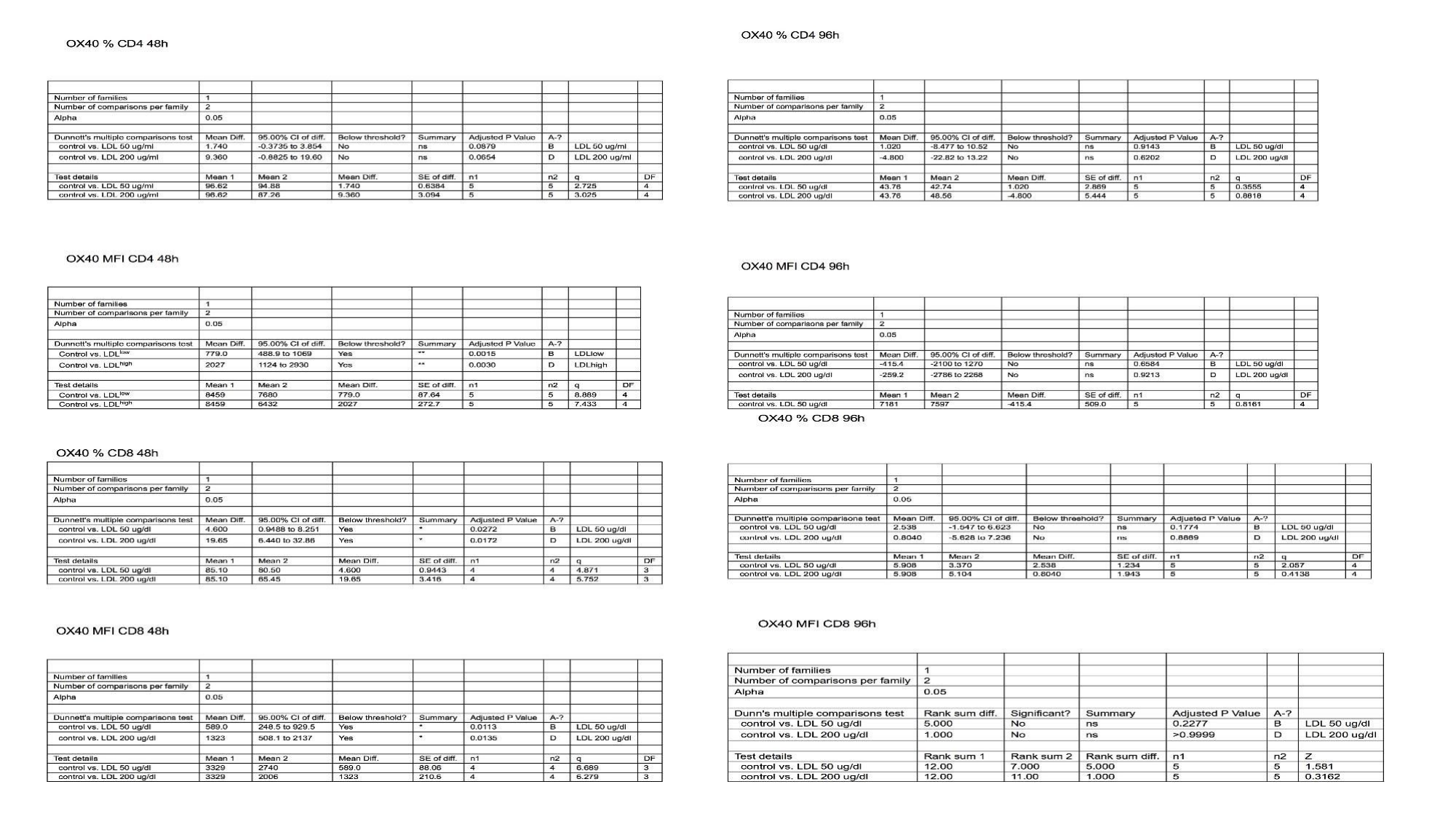

## Slide 19
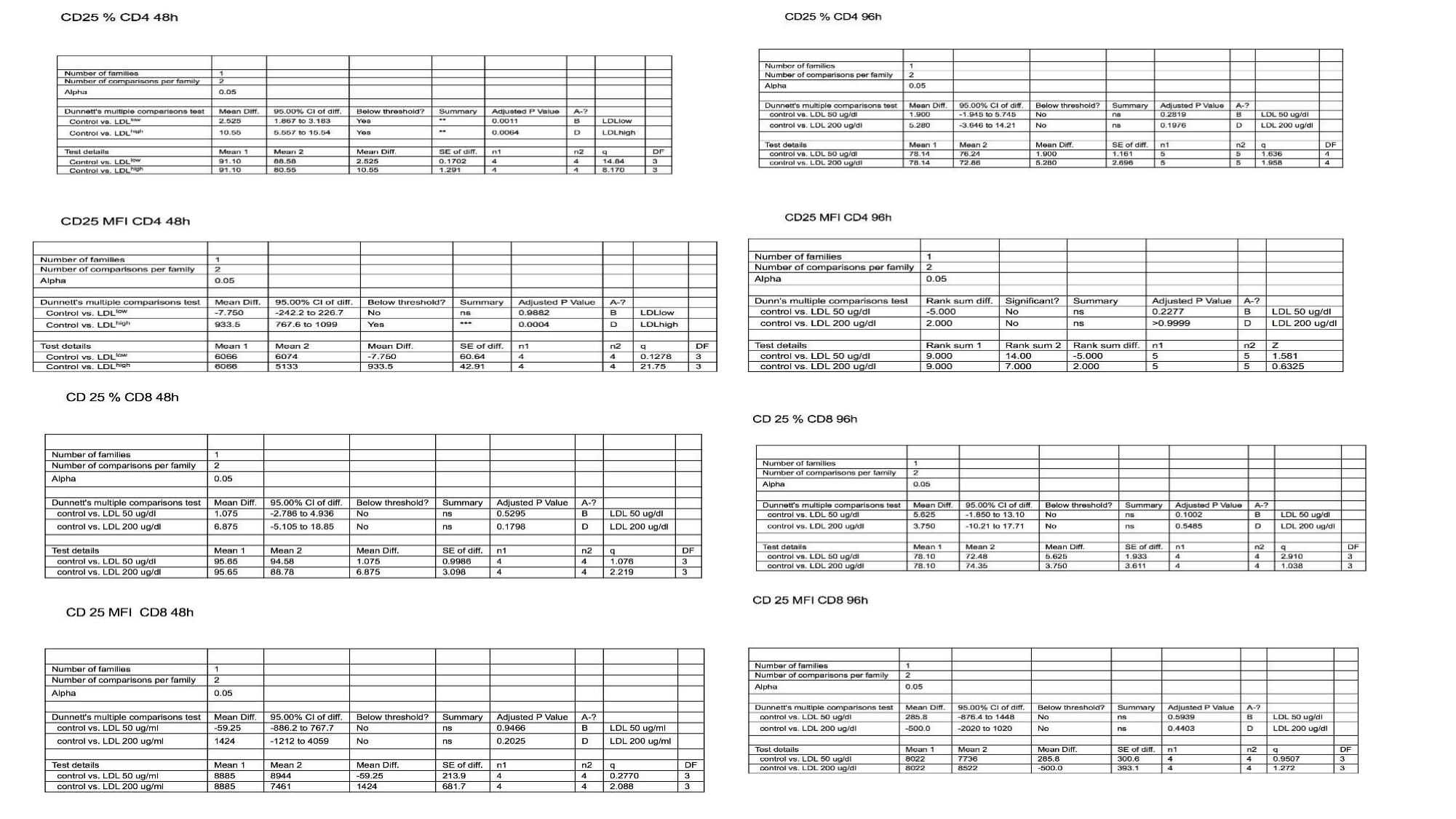

## Slide 20
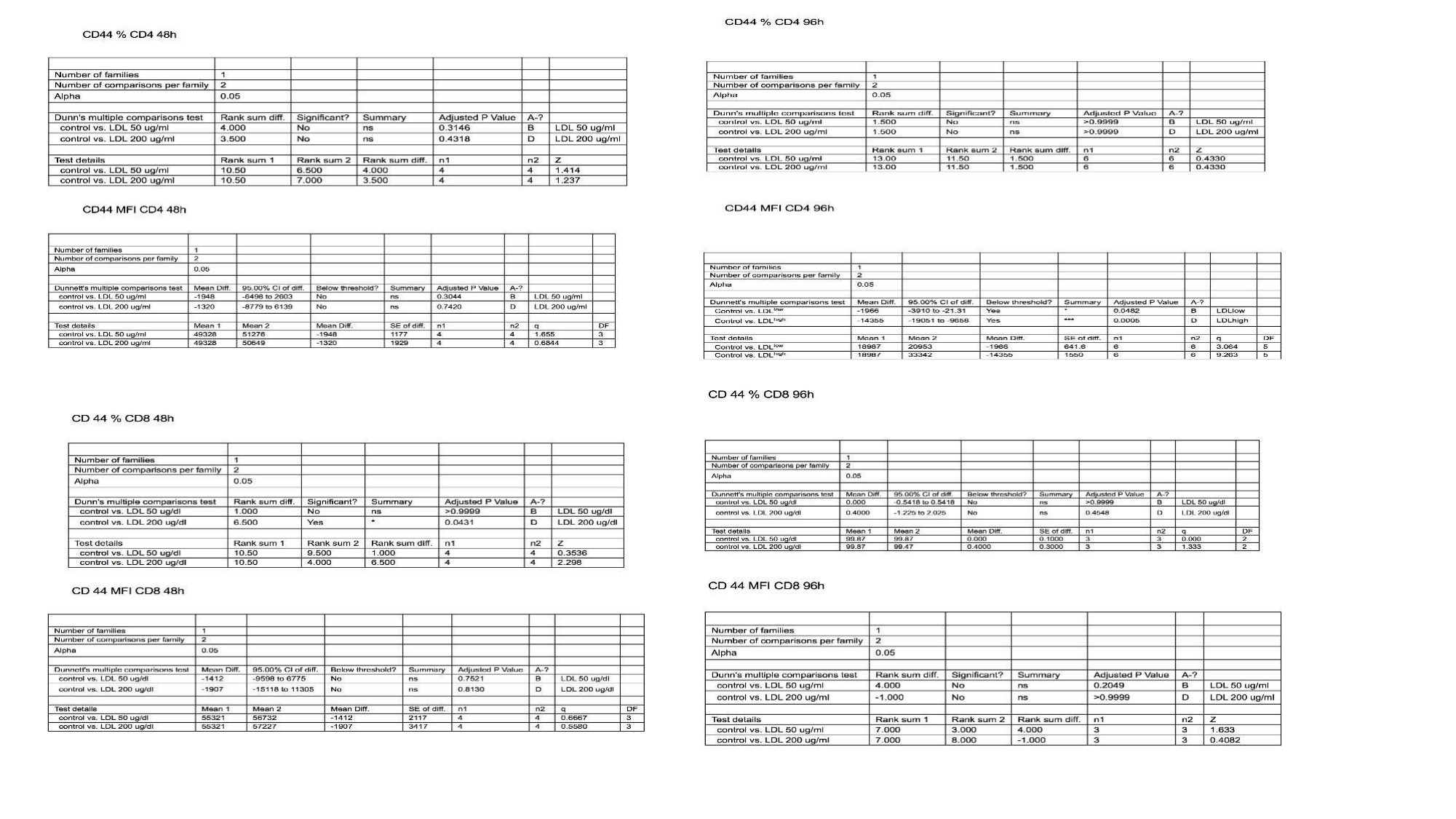

## Slide 21
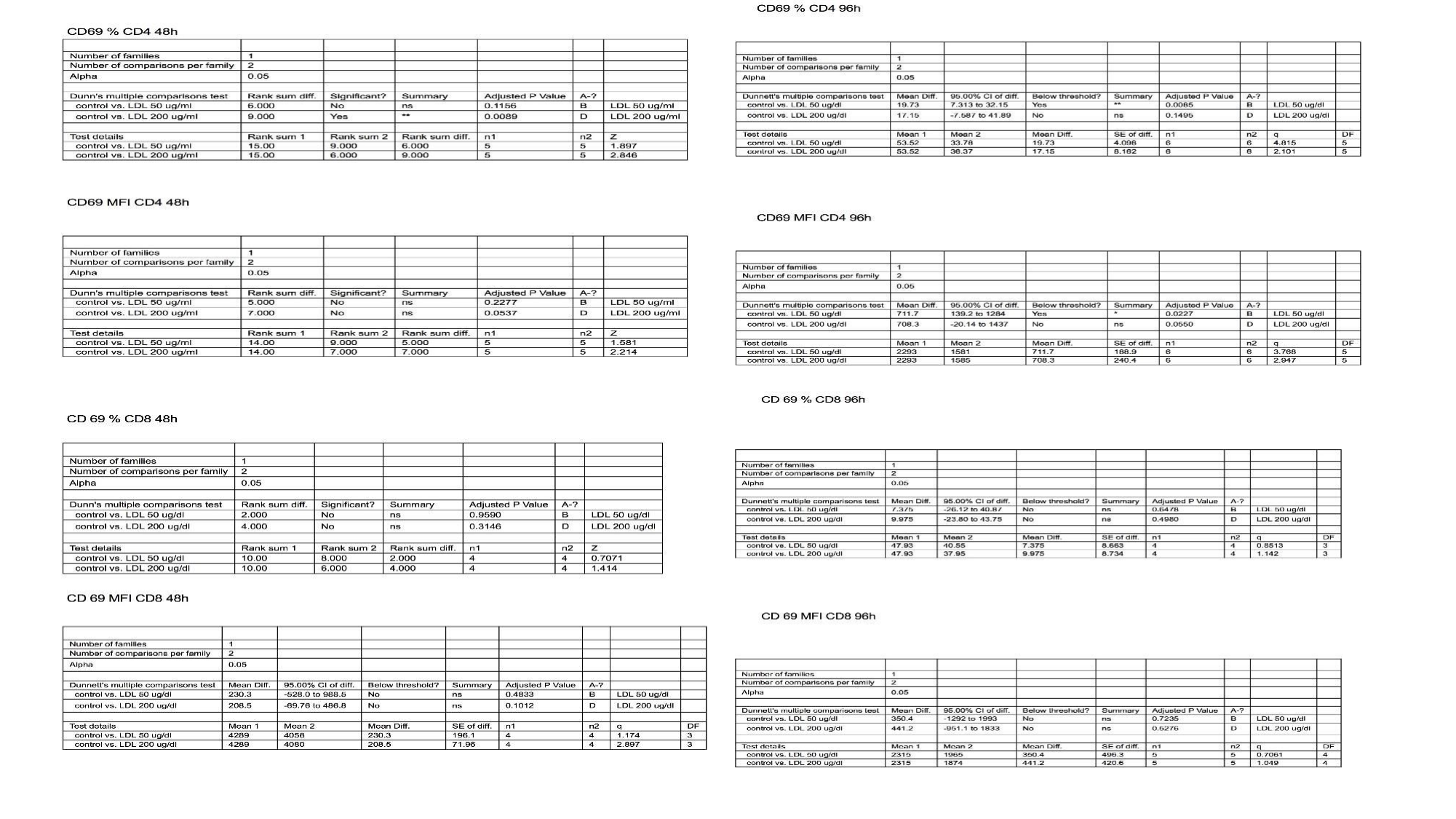

## Slide 22
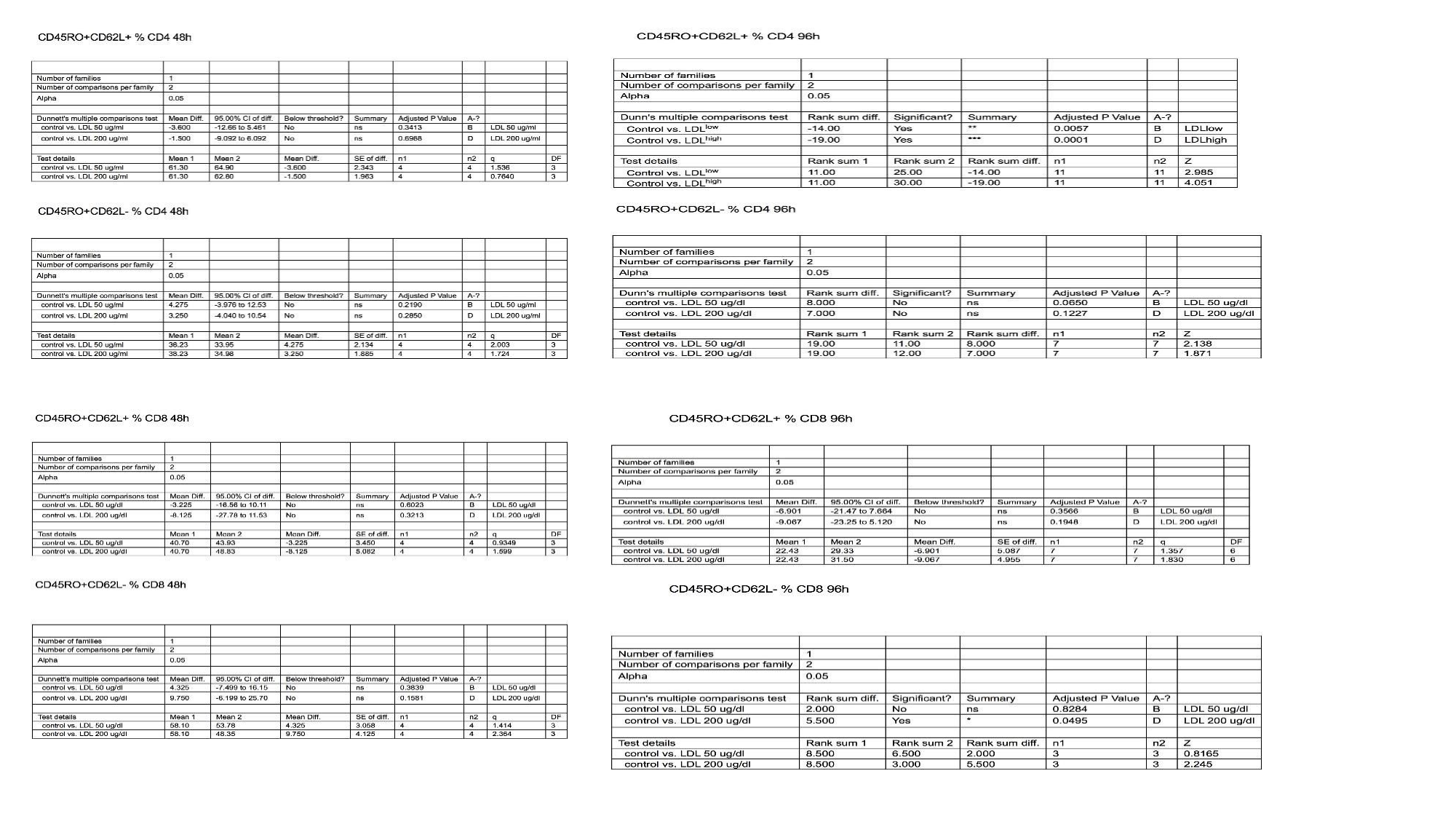

## Slide 23
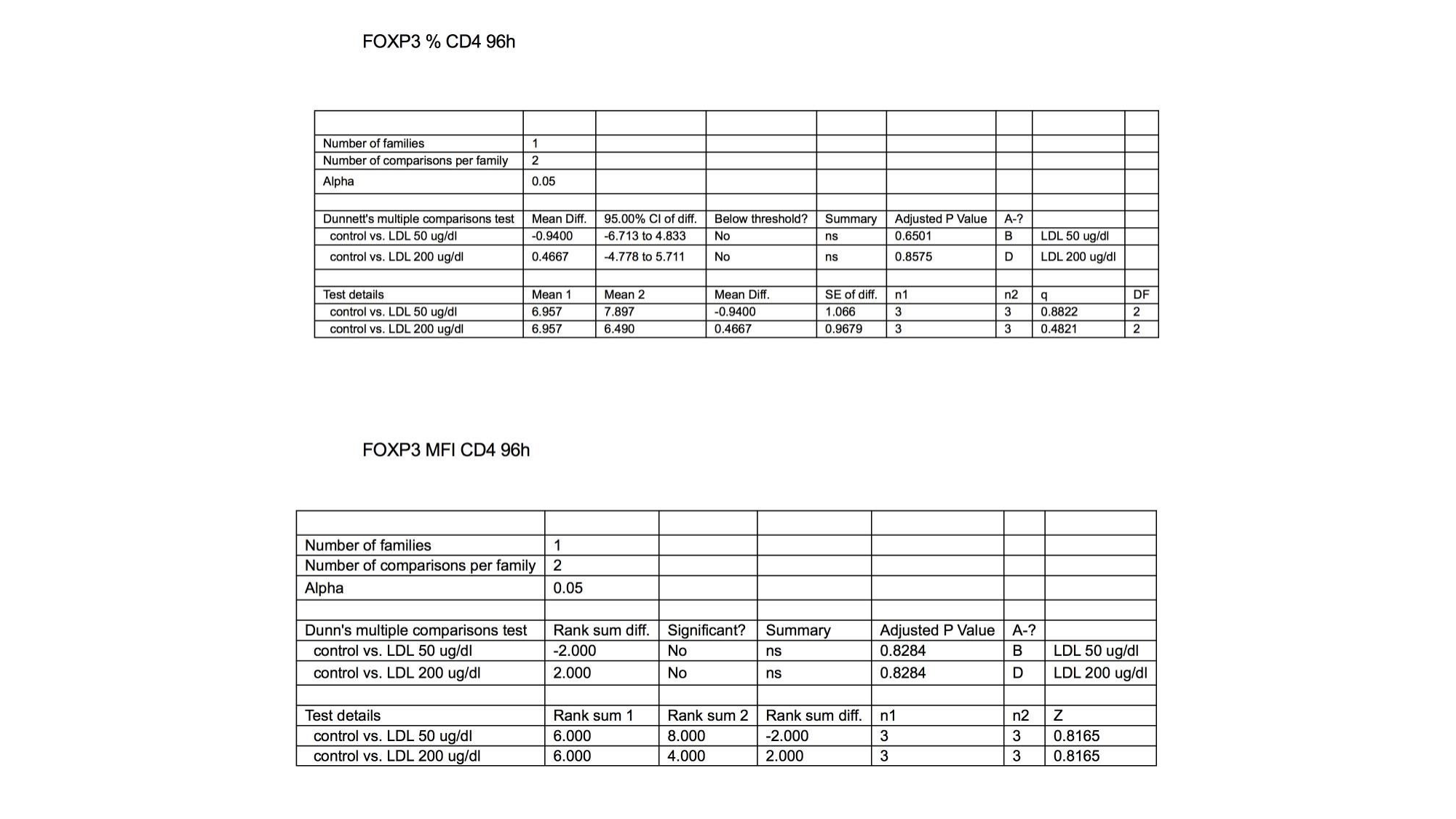

## Slide 24
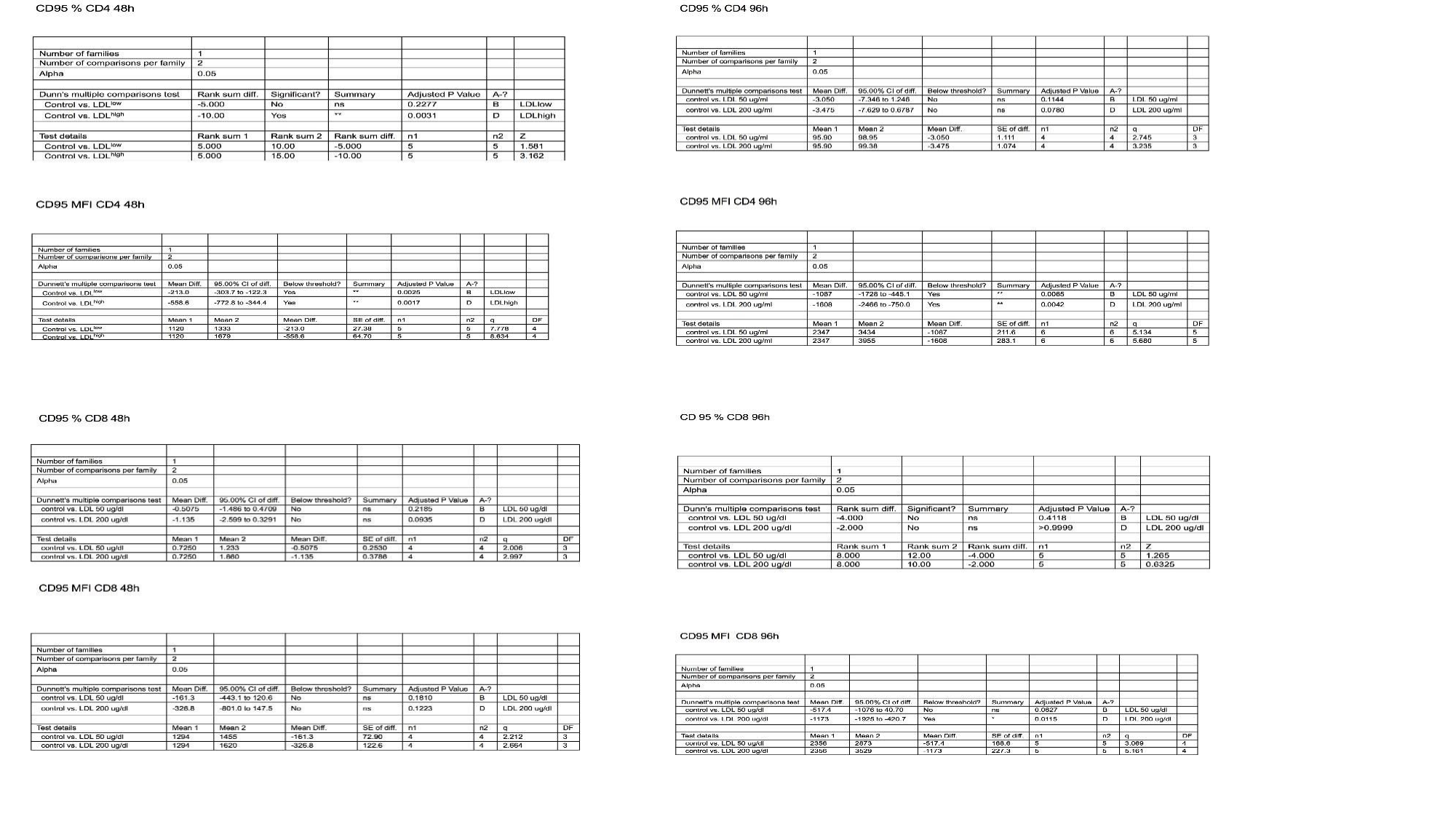

## Slide 25
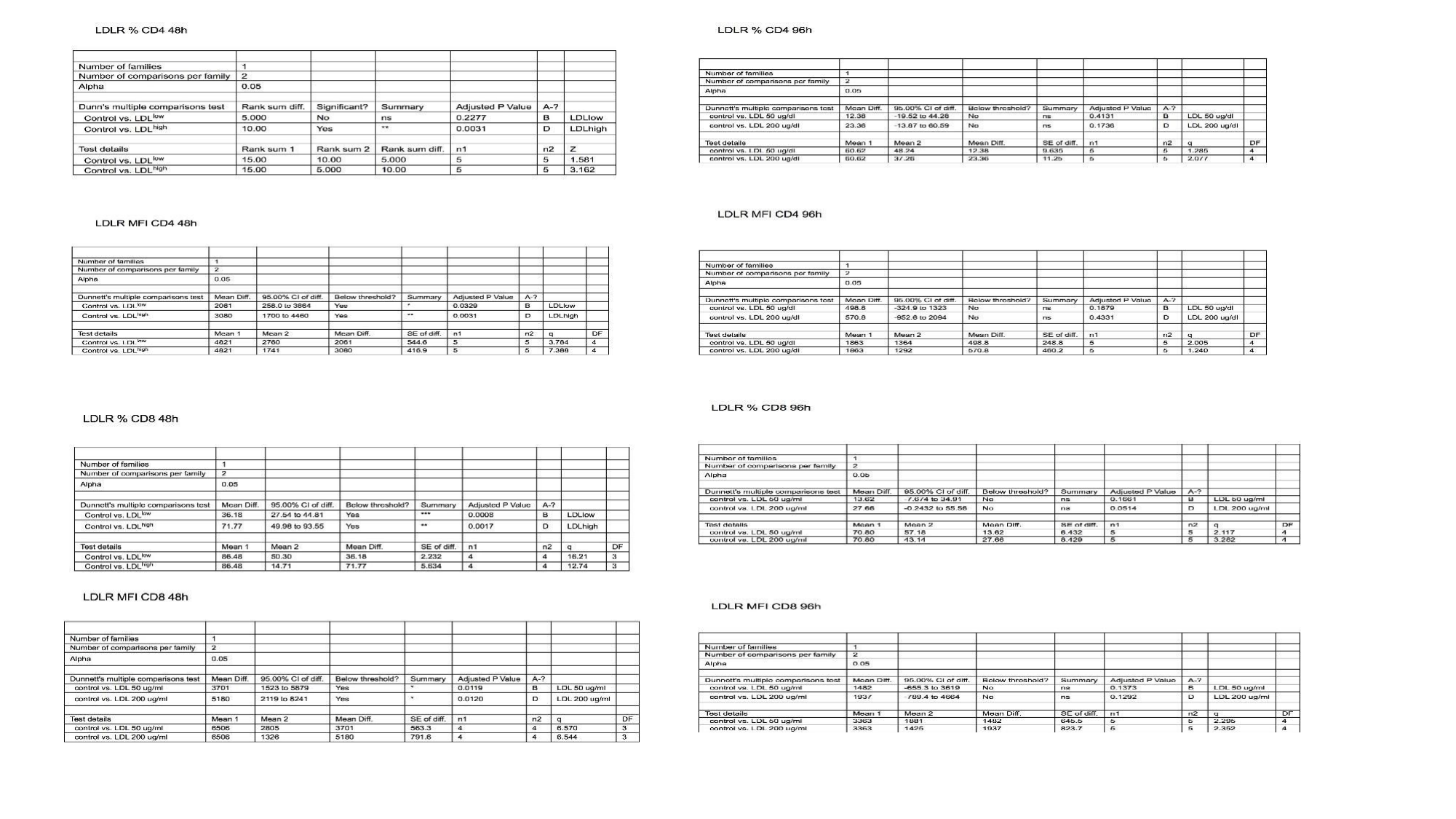

Supplement: Supplementary Data Sheet 1 — FACS gating MACS isolated CD4+ or CD8+ T cells [file DataSheet_1.zip › Supplement 1/Supplemental Data S2 Original Surface Marker Statistics (not corrected).PPTX]
