# Supplementary figures and images for: Low-density lipoprotein balances T cell metabolism and enhances response to anti-PD-1 blockade in a HCT116 spheroid model
Source: Front Oncol. 2023 Jan 27;13:1107484. doi: 10.3389/fonc.2023.1107484 (PMC9911890; doi:10.3389/fonc.2023.1107484)

## Slide 1
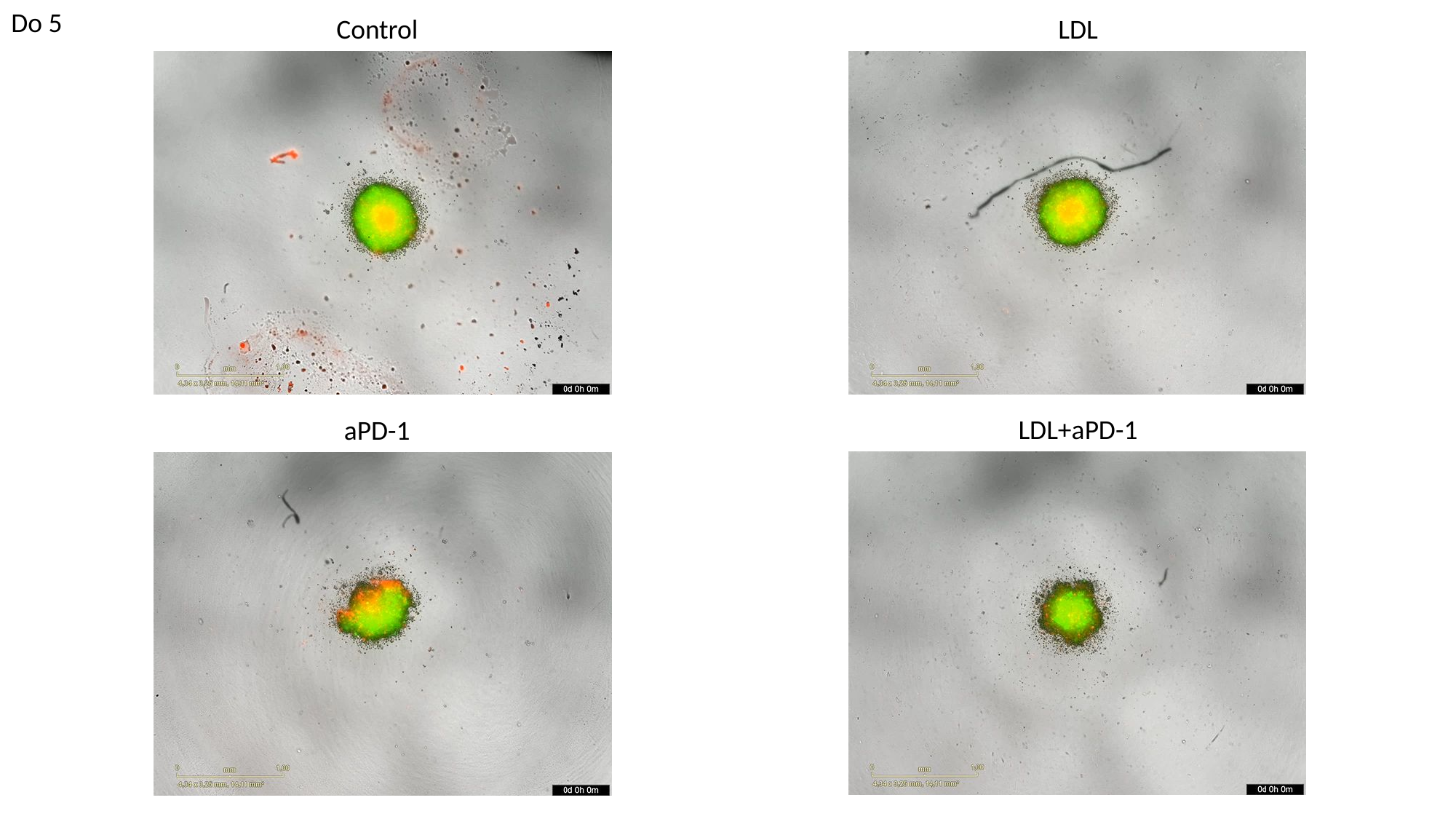

Do 5
Control
LDL
LDL+aPD-1
aPD-1

## Slide 2
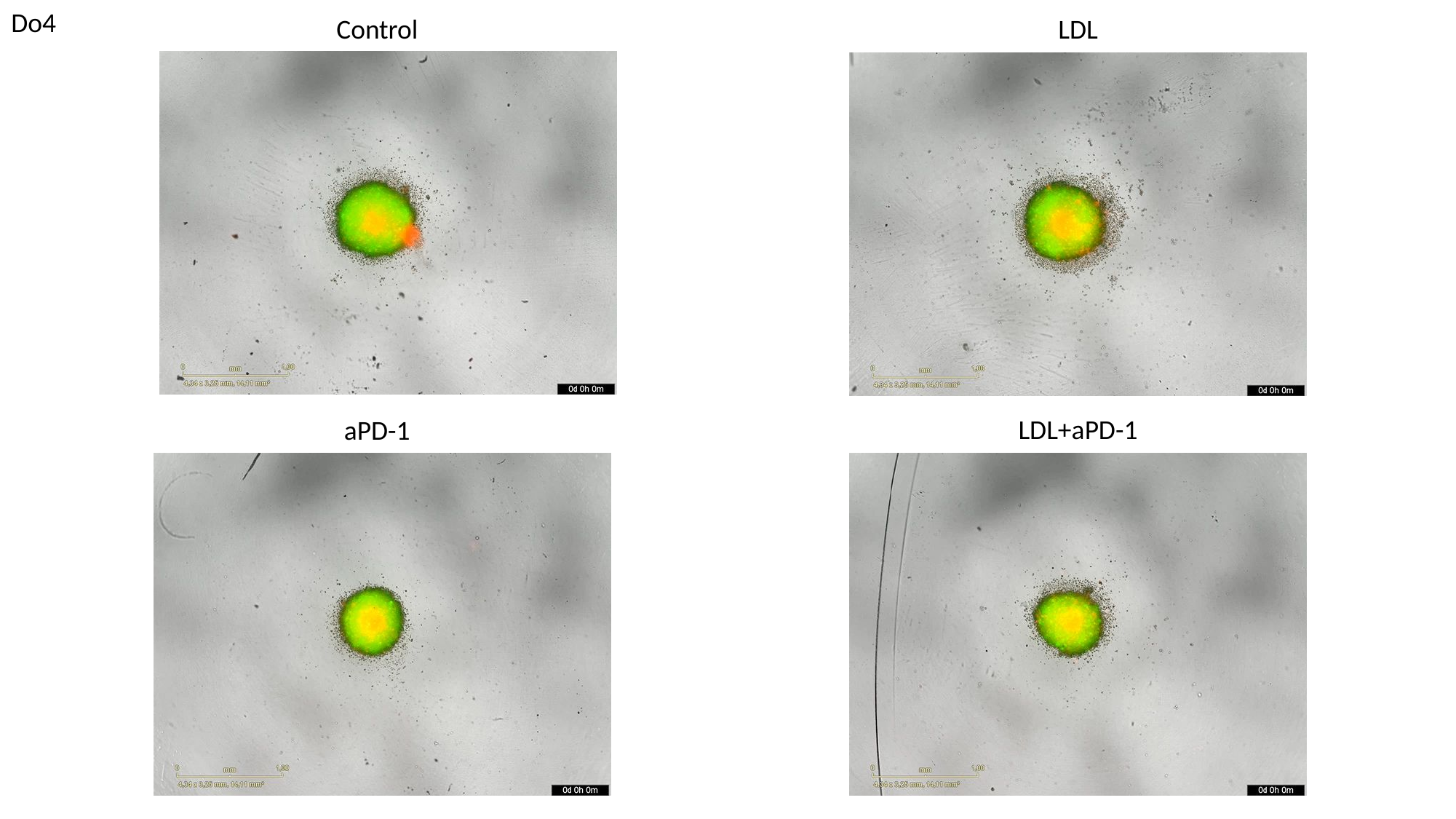

Do4
Control
LDL
LDL+aPD-1
aPD-1

## Slide 3
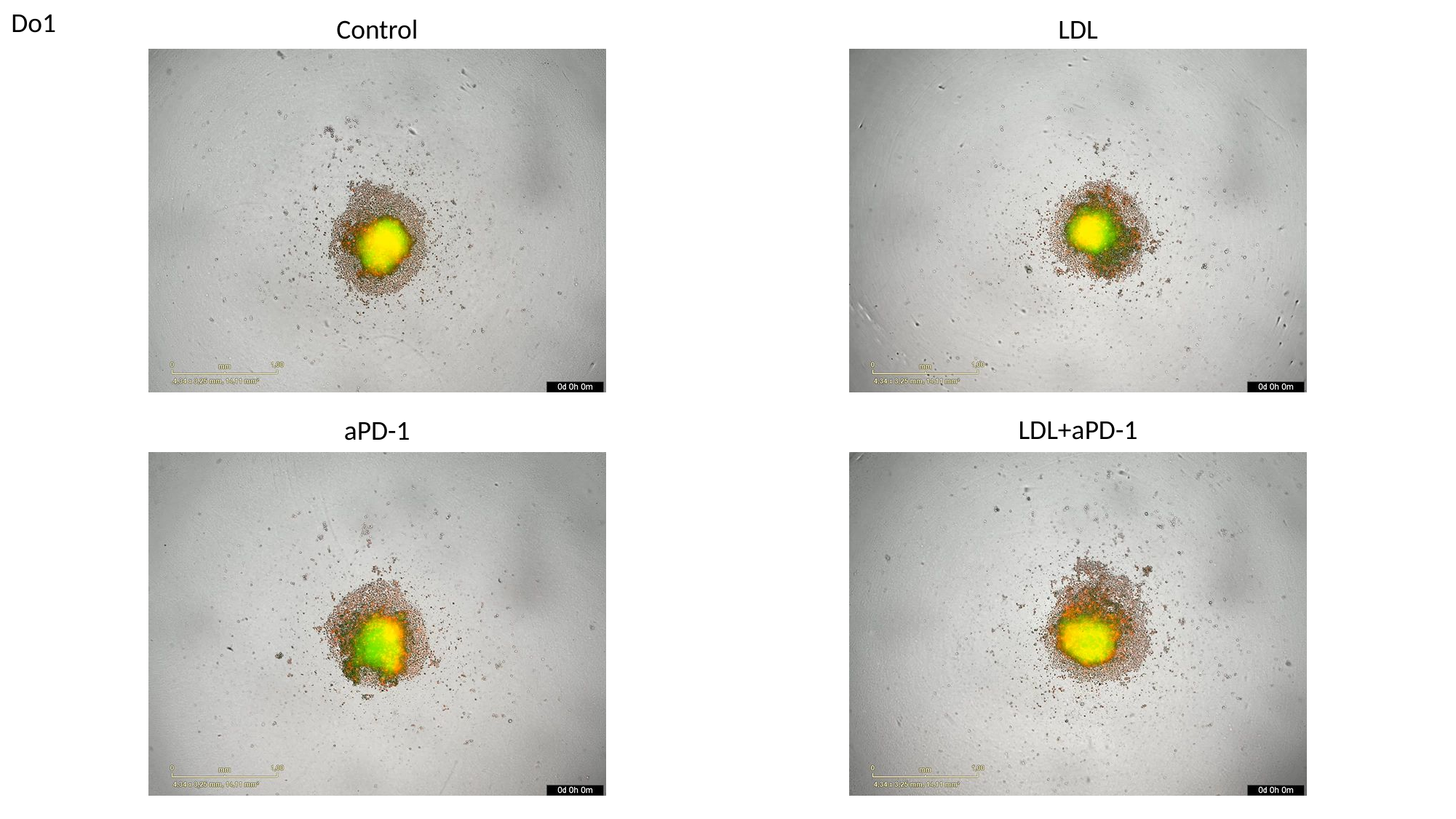

Do1
Control
LDL
LDL+aPD-1
aPD-1

Supplement: Supplementary Video 1 — Incucyte Live Cell Imaging over 48 h. [file DataSheet_2.zip › Supplement 2 Videos/Video S1 Incucyte Live Cell Imaging over 48 h Part1.PPTX]

## Slide 1
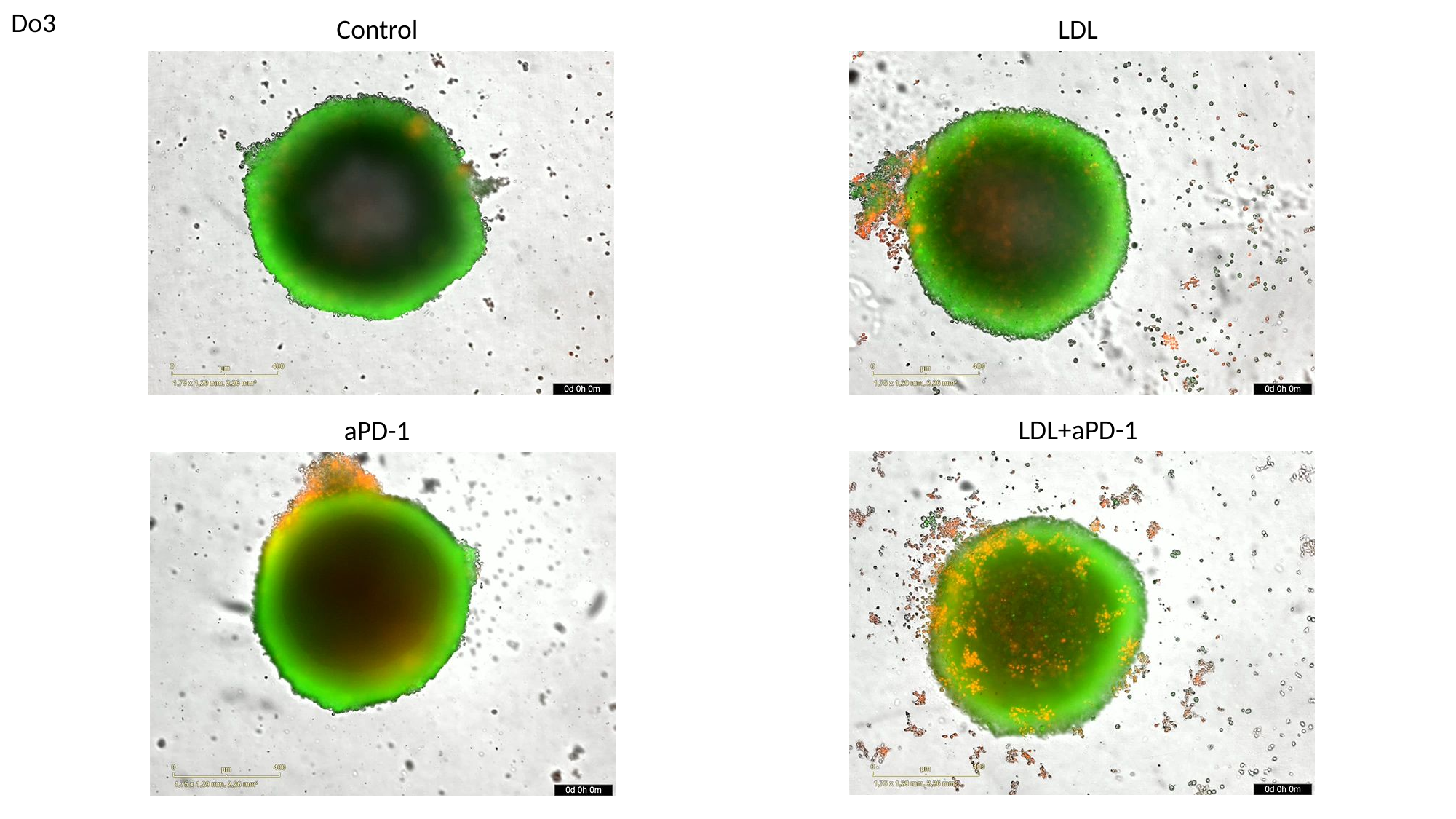

Do3
Control
LDL
LDL+aPD-1
aPD-1

## Slide 2
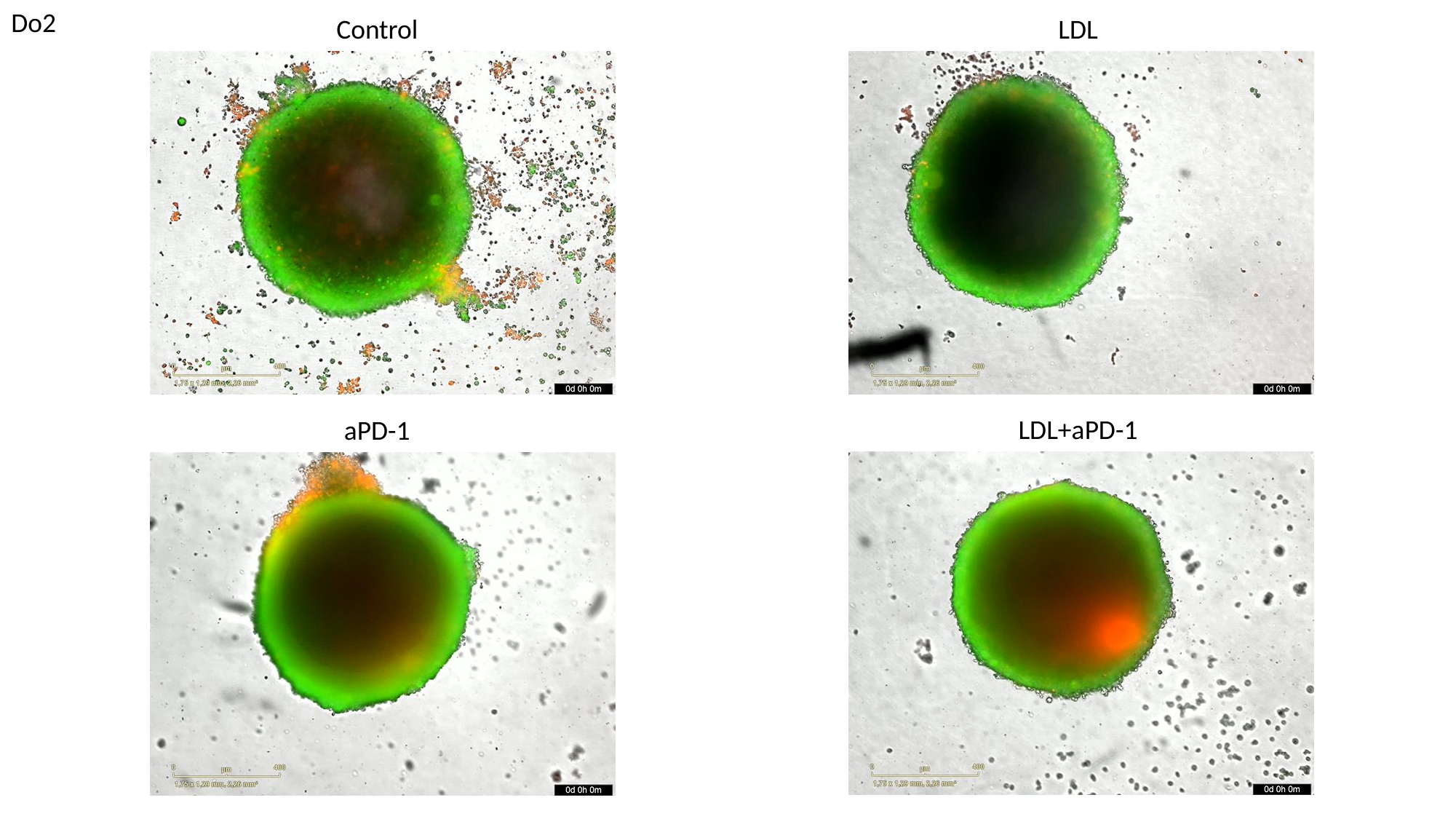

Do2
Control
LDL
LDL+aPD-1
aPD-1

Supplement: Supplementary Video 1 — Incucyte Live Cell Imaging over 48 h. [file DataSheet_2.zip › Supplement 2 Videos/Video S1 Incucyte Live Cell Imaging over 48 h Part2.PPTX]
